# Antisclerostin Effect on Osseointegration and Bone Remodeling

**DOI:** 10.3390/jcm12041294

**Published:** 2023-02-06

**Authors:** Bárbara Alexandra do Amaral Couto, Juliana Campos Hasse Fernandes, Mariana Saavedra-Silva, Hernan Roca, Rogério Moraes Castilho, Gustavo Vicentis de Oliveira Fernandes

**Affiliations:** 1 Faculty of Dental Medicine, Universidade Católica Portuguesa, 3505-606 Viseu, Portugal; 2 Private Practice, Ann Arbor, MI 48109, USA; 3 Departamento de Cirurgía (Área de Estomatología), Facultad de Medicina, Universidad de Salamanca, 37007 Salamanca, Spain; 4 McCauley-Roca Lab’s, University of Michigan School of Dentistry, Ann Arbor, MI 48109, USA; 5 Periodontics and Oral Medicine Department, University of Michigan School of Dentistry, Ann Arbor, MI 48109, USA

**Keywords:** antisclerostin, bone formation, bone remodeling, osseointegration, sclerostin antibody

## Abstract

**Objective**: This study reviewed the literature on local or systemic administration of antisclerostin, presenting results associated with osseointegration of dental/orthopedic implants and stimulation of bone remodeling. **Materials and Methods**: An extensive electronic search was conducted through MED-LINE/PubMed, PubMed Central, Web of Science databases and specific peer-reviewed journals to identify case reports, case series, randomized controlled trials, clinical trials and animal studies comparing either the systemic or local administration of antisclerostin and its effect in osseointegration and bone remodeling. Articles in English and with no restriction on period were included. **Results**: Twenty articles were selected for a full-text, and one was excluded. Finally, 19 articles were included in the study (16 animal studies and 3 randomized control trials). These studies were divided into two groups, which evaluated (i) osseointegration and (ii) bone remodeling potential. Initially 4560 humans and 1191 animals were identified. At least 1017 were excluded from the studies (981 humans and 36 animals), totaling 4724 subjects who completed (3579 humans and 1145 animals). (a) Osseointegration: 7 studies described this phenomenon; 4 reported bone-implant contact, which increased in all included studies. Similar results were found for bone mineral density, bone area/volume and bone thickness. (b) Bone remodeling: 13 studies were used for description. The studies reported an increase in BMD with sclerostin antibody treatment. A similar effect was found for bone mineral density/area/volume, trabecular bone and bone formation. Three biomarkers of bone formation were identified: bone-specific alkaline phosphatase (BSAP), osteocalcin and procollagen type 1 N-terminal Pro-peptide (P1NP); and markers for bone resorption were: serum C-telopeptide (sCTX), C-terminal telopeptides of type I collagen (CTX-1), β-isomer of C-terminal telopeptides of type I collagen (β-CTX) and tartrate-resistant acid phosphatase 5b (TRACP-5b). There were limitations: low number of human studies identified; high divergence in the model used (animal or human); the variance in the type of Scl-Ab and doses of administration; and the lack of reference quantitative values in the parameters analyzed by authors’ studies (many articles only reported qualitative information). **Conclusion**: Within the limitations of this review and carefully observing all data, due to the number of articles included and the heterogeneity existing, more studies must be carried out to better evaluate the action of the antisclerostin on the osseointegration of dental implants. Otherwise, these findings can accelerate and stimulate bone remodeling and neoformation.

## 1. Introduction

Sclerostin is a glycoprotein encoded in humans by the SOST gene [1,2]. It is located on chromosome 17q12-q21 [3], with a C-terminal cysteine knot-like (CTCK) domain. It has a similar sequence also to DAN (Differential screening-selected gene Aberrative in Neuroblastoma), an antagonist’s family of the bone morphogenetic protein (BMP). Sclerostin is primarily produced and secreted by osteocytes [4,5]. Moreover, it is a negative key regulator of osteoblastic functions [6]. It inhibits osteoblast differentiation and bone formation by inhibiting the Wnt signaling pathway after binding with LRP5 and 6 (Wnt-coreceptor) [7,8,9,10].

This canonical Wnt signaling (Wnt/β-catenin pathway) is essential in bone healing [11,12,13,14,15,16,17]. It promotes pre-osteoblast proliferation and osteo-induction, enhances survival of all cells of the osteoblast lineage, inhibits differentiation of mesenchymal stem cells (MSCs) into chondrocytes and adipocytes and controls osteoclast maturation by regulating RANKL levels in osteoblast receptors [18]. Furthermore, it controls skeletal development as well as bone homeostasis. Alterations in several Wnt pathway members have caused skeletal abnormalities [19,20,21,22]. Conversely, low levels of sclerostin or SOST gene mutations can implicate several genetic skeletal disorders with high bone mineral density (BMD), such as sclerosteosis and van Buchem disease [1,2,6]. Conversely, gene over-expression leads to osteopenia [23].

Besides the well-known influence of Wnt signaling on bone formation, its role in the immune system has also received attention [24]. The effect of the interaction between sclerostin and the immune system on osseointegration needs to be elucidated. Furthermore, implant surface topography has been shown to direct immune response and osteoclastic precursor-surface interactions. Understanding surface topography’s influence in combination with sclerostin on implant adherent cells can show how osseointegration can follow, which will help control/manipulate clinical outcomes [25].

In this scenario, researchers have sought how to control sclerostin stimulation [26]. The suppression effect can be exerted by the parathyroid hormone [27,28], mechanical loadings [29], cytokines (prostaglandin E2) [30], onco-statin M, cardiotrophin-1 and leukemia inhibitory factor [31]. Moreover, systemic administration of a monoclonal sclerostin antibody (Scl-Ab) can significantly increase new bone and its strength [32,33,34]. Scl-Ab also elevates Wnt signaling. It improves bone-implant contact (BIC) [35], increases bone mass [36,37] and enhances bone performance with aging [38]. Furthermore, it revealed an enhancement of the cortical and trabecular bone, favoring the mechanical fixation of femoral implants [39]. For alveolar bone defects, there was an increase in BIC, bone volume fraction (BVF) and bone area fill. This fact indicated an improvement in bone regeneration and implant osseointegration [40].

Furthermore, antisclerostin induced robust clinical increases for BMD. This became a promising treatment for osteoporosis [41,42,43]. Likewise, an interest in Scl-Ab application in Dentistry has emerged. The main areas involved are bone regeneration and osseointegration. A recent publication [44] also arrived at relevant results for Scl-Ab systemic contrasting with local use.

In implant dentistry, high success/survival rates were reported for dental implants [45,46,47]. The rates achieved more than 95% [48] and, in the long term (ten-years follow-up), a 96.4% survival rate [49]. Thereby, implants are a predictable and reliable treatment. They can treat around 69% of adults (aged between 35 and 44) that had lost at least one permanent tooth. They can also treat older people (more than 70 years old), whereby 26% have already lost all their permanent teeth [50].

Moreover, an estimated 100,000–300,000 implants are placed annually [51]. The expectation in the US and European markets were for around $4.2 billion worth in 2022 only [52]. Nonetheless, there are still challenges. The main question concerns the acceleration of the osseointegration process. This point can benefit patients, permitting quicker rehabilitation. Then, the implant can have surface modifications or be used with bone antiresorptive/anabolic agents [53,54], such as Scl-Ab.

With this background, the goal of this study was to review the literature on local/systemic antisclerostin administration, presenting results associated with dental/orthopedic implants’ osseointegration and bone remodeling.

## 2. Materials and Methods

This review was conducted following the Preferred Reporting Items for Systematic reviews and Meta-Analysis (PRISMA) guidelines [55]. The protocol for this review was registered on PROSPERO (CRD42021236778). The focused question was determined according to the Population, Intervention, Comparison and Outcome (PICO) strategy [56], “If a subject receives systemic or local antisclerostin, could it cause acceleration of osseointegration or improve bone remodeling?”

### 2.1. Information Sources and Search Strategy

An extensive electronic search was conducted through MEDLINE (PubMed), PubMed Central (PMC) and Web of Science databases. Specific peer-reviewed journals were also analyzed: *Biomed Research International, Cancers, Current Osteoporosis Reports, Frontiers in Bioengineering and Biotechnology, International Journal of Molecular Sciences, International Journal of Nanomedicine, Journal of Dental Research, Journal of Functional Biomaterials, Materials, Osteoporosis International* and *PloS one.* The following keywords were used: sclerostin OR antisclerostin OR sclerostin antibody OR Romosozumab OR Blosozumab AND osseointegration AND bone formation OR “newly formed bone” OR “new bone” AND dental implant OR “dental implants” OR implant, with a platform-specific search strategy combining terms and text words with Booleans. An additional manual search was performed on the references of included articles to identify relevant publications. There is no date restriction, but only English language was considered.

Two reviewers (G.V.O.F and B.A.A.C.) independently performed the electronic and manual searches. The publications obtained from the search were imported into software (EndNote 20.1) and subsequently screened.

### 2.2. Inclusion Criteria

This review was based on any experimental in vivo (animal or human) study involving Scl-Ab effectiveness analysis when administrated systemically or locally, resulting in dental implant osseointegration or bone remodeling. Case reports, case series, randomized controlled trials, clinical trials and animal studies were included. There was a language restriction (English) for selection of the studies and no limitation for the period (years). Studies were included where the subject had necessary implant treatment; studies analyzing implant osseointegration, clearly reporting the results and survival and/or failure rates, were included; if applicable, only the article with the most extended follow-up was included when involving the same patient cohort (population).

### 2.3. Exclusion Criteria

Books or chapter, posters and e-posters, editorial letters, patents, reports based on questionnaires, interviews, in vitro studies, in silico research and systematic reviews/meta-analyses were excluded. Moreover, articles presenting lack of information on osseointegration, or bone remodeling or dose/period of drug administration were also excluded.

### 2.4. Selection of Studies and Data Extraction

Duplicate studies were excluded. The remaining articles were initially screened for eligibility by title and abstract. Further examination regarding inclusion and exclusion was subsequently made through the full-text analysis. The full text of any title or abstract that did not provide enough information according to the inclusion criteria was also excluded. Any disagreement between the reviewers (G.V.O.F and B.A.A.C.) was discussed with a third author (J.C.H.F.). Cohen’s kappa test was adopted to evaluate reviewers’ agreement on title, abstract and full-text selection.

The reviewers extracted the data independently from the selected articles for further analysis using data extraction tables, which included the following parameters: year, the country in which the study was developed, type of study, species included (if applicable), sample size (initial and final), age (mean), gender, control drug (name), administration route, dosage (unit), period of treatment and implant.

## 3. Results

### 3.1. Study Selection

**Figure 1:**
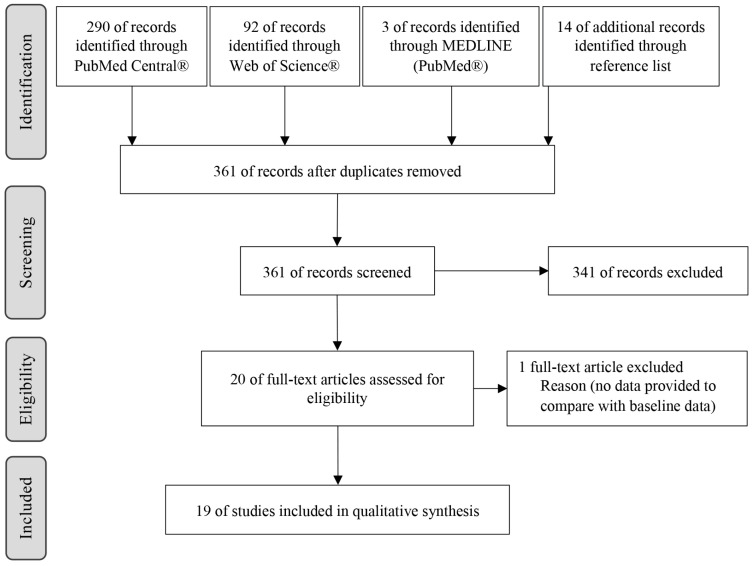
Search strategy and study selection.

A total of 385 records were identified in the databases and 14 through the manual search. After removing duplicates (24 studies), 361 records were screened. After analysis of the title and abstract, 341 studies were excluded, and the remaining 20 records were evaluated by full text. One paper was excluded due to a lack of initial information available. Finally, 19 articles were finally included in this study (Figure 1).

### 3.2. Study Characteristics and Details

Of the 19 articles selected, 16 were animal studies and three control trials [RCTs]) (Table 1). In total, 4560 humans and 1191 animals (906 Sprague-Dawley rats, 128 Wistar rats, 102 Lewis rats and 55 Cynomolgus monkeys) were initially identified and 1017 were excluded (981 humans and 36 animals) after analyzing the eligibility criteria, leaving 3579 humans and 1145 animals to be evaluated. Finally, 582 female and 522 male rats, 12 female and 29 male monkeys were enrolled and 3564 women and 15 men (Table 1).

### 3.3. Included Studies

Ominsky et al. (2011), Virk et al. (2013) and Liu et al. (2018) [58,61,63] described two independent studies in the same article (Table 1 and Table 2). The first one [58] used two different samples, females in one study and male Sprague-Dawley rats in the other. Ominsky et al. used studies on two species [63] (Sprague-Dawley mice and Cynomolgus monkeys), which underwent osteotomy in fibular midshaft.

In four studies [33,35,58,59], ovariectomy surgery was performed to induce osteopenia. In the initial sample size, 223 ovariectomized (OVX) and 153 sham-ovariectomized (Sham) rats were identified. In one of the studies [33], five rats were excluded after surgery, but the authors did not mention from which group they were excluded. In the remaining studies, 151 OVX and 81 sham rats completed (Table 1). One article [33] did not mention the age of the animals used.

For the animal studies, one article reported that no antibody was applied as a control [57]. Seven studies [33,34,39,58,64,66] utilized saline solution and six others used a vehicle as control [35,38,59,63,65]. Three studies used phosphate-buffered saline solution (PBS) [40,61]. One study had two different controls [60] using PBS in healthy animals and vehicle in animals for which an experimental periodontitis model was induced. Another study [62] reported the use of an intraarticular (ia) particle vehicle and subcutaneous (sc) antibody as control (Table 2), whereas for two human studies [41,43] placebo was used as control and [67] Alendronate in another.

#### 3.3.1. Dosages Used

Five articles reported the administration of 25 mg/kg sc of Scl-Ab III (sclerostin antibody III/murine sclerostin antibody, Amgen and UCB Pharma, Thousand Oaks, California) twice a week in rats [33,35,61,63,64]. Virk et al., 2013 [61] administered 25 mg/kg of Scl-Ab III without mentioning the administration route. Ominsky et al., 2011 [63] performed a study in monkeys, which administered 30 mg/kg sc of Scl-Ab V (Humanized sclerostin antibody, Amgen and UCB Pharma) every two weeks. Three studies [34,38,66] used two different dosages of Scl-Ab III, 5 mg/kg or 25 mg/kg sc twice a week, while two studies [40,57] did not report the type of antibody used. Korn et al. (2019) [57] administered 100 mg/kg of Scl-Ab intravenous (iv) and Yu et al. (2018) [40] referred to the administration of 25 mg/kg of Scl-Ab subcutaneously.

Liu et al. 2018 [58] reported the administration of Scl-Ab VI and the association of Scl-Ab VI with DKK1 antibody (Scl-Ab VI + DAB) in OVX rats. However, the drug dosage differed between studies. One study administered 18.2 mg/kg sc twice a week (Scl-Ab VI); 18.2 mg/kg (Scl-Ab VI) and 18.1 mg/kg sc twice a week (DAB); whereas, in the other study, 25 mg/kg sc twice a week (Scl-Ab VI) and 25 mg/kg (Scl-Ab VI) and 25 mg/kg sc was used twice a week (DAB).

One study [59] reported the administration of 25 mg/kg of Scl-Ab (Sclerostin antibody, Amgen, Thousand Oaks, California) sc twice a week, 60 µg/kg of PTH 1-34 (human Parathyroid Hormone 1–34, Bachem, Torrance, California) sc thrice a week and the association of these two drugs mentioned above (Scl-Ab + PTH 1-34) in OVX rats. Another study [60] reported a systemic administration of 25 mg/kg sc twice a week of Scl-Ab III and a local administration of 5 µL of 35.6 mg/mL of solution per site twice a week, giving a total of 15 µL per animal per treatment session, in rats submitted to experimental periodontitis model (EP rats).

Liu et al. (2012) [62] administrated 50 µL ia of polyethylene (PE) suspension once a week associated with antibody vehicle or 25 mg/kg sc of Scl-Ab III twice a week. Virdi et al. (2012) [39] used 25 mg/kg subcutaneously of Scl-Ab (murine sclerostin antibody Amgen, Thousand Oaks, California). Ominsky et al. (2010) [65] applied three different dosages, 3 mg/kg, 10 mg/kg, or 30 mg/kg sc of Scl-Ab IV (humanized sclerostin-neutralizing monoclonal antibody) once a month in monkeys.

In human studies, Saag et al. (2017) [67], during an initial period, administered 210 mg sc of Romosozumab once a month, followed by oral administration of 70 mg of Alendronate once a week. In McClung et al.’s (2014) study [41] 140 mg or 210 mg was administered once every three months, or 70 mg, 140 mg, or 210 mg once a month sc of Romosozumab, 70 mg sc of Alendronate once a week or 20 μg sc of Teriparatide once a day.

In the previous study [37] Romosozumab was administrated subcutaneously and divided into six cohorts: four female cohorts (1 mg/kg every 2 weeks, 2 mg/kg every 4 weeks, 2 mg/kg every 2 weeks, 3 mg/kg every 4 weeks, Cohorts 1, 2, 3 and 4, respectively) and two male cohorts (1 mg/kg every 2 weeks, 3 mg/kg every 4 weeks, Cohorts 5 and 6, respectively). When the last woman received the dose from cohort 2, she was followed for 6 weeks; they evaluated the safety and laboratory findings before moving to cohorts 3 and 4. Cohort 5 ran simultaneously with any ongoing cohorts and cohorts 4 and 6 started simultaneously.

#### 3.3.2. Implant Characteristics

Four studies reported implant placement [35,39,40,57]. One study used titanium implants with two types of surface treatment (titanium sandblasted thermally acid-etched surface (reference-coated implant) and zoledronate-stearate spray-coated surface (ZOL-coated implant) [57]. Another study [38] used commercially pure titanium (cp-Ti) cylindrical solid with titanium plasma-sprayed surface implant one month after the first right maxillary molar extraction. Finally, Virdi et al. (2012) and Virdi et al. (2015) used cp-Ti with dual acid-etched surface implants [35,39].

Only one study [62] used titanium screws with dual acid-etched surface. In one of the studies, Omnisky et al. (2011) [63]. used stainless steel K-wire. One other study used stainless steel screws for mechanical tests and polymethylmethacrylate (PMMA) screws for micro-CT to avoid radiographic artifacts [64]. Nevertheless, the remaining studies did not install any implant.

### 3.4. Osseointegration and Bone-Implant Contact (BIC)

Seven studies [35,39,40,57,62,63,64] described the osseointegration phenomena (Tables S1–S6) and 4 reported BIC [35,39,40,57]. Korn et al. (2019) [57] analyzed BIC by histomorphometry and μCT, reporting similar BIC values 2 weeks following Scla-Ab treatment in both groups. After 4 weeks, BIC values increased in the ZOL-coated implants and decreased in the control-coated implants. Through µCT, the authors reported the highest increase in BIC 4 weeks after administration of Scl-Ab combined with ZOL-coated implants (Table S1).

The other three studies [35,39,40] only reported qualitative information. Yu et al. (2018) [40] found a significantly greater BIC than the control group at 28 days and non-significant differences in early points. This evidence is also supported by Virdi et al. (2012) [39]. Virdi et al. (2015) [35] saw an increase over time after Scl-Ab treatment, more notable in sham rats.

#### 3.4.1. Bone Mineral Density (BMD)

Four articles studied BMD [40,57,63,64] (Table S1). Korn et al. (2019) [57] reported a significant increase in cancellous BMD with ZOL-coated implants and a decrease with control-coated implants 4 weeks after implant placement. The ZOL-coated implant associated with Scl-Ab reported almost a two-fold increase compared to the control-coated implant group. Only one study [40] noted no differences between groups (control and Scl-Ab).

Two studies [64,65] reported values in specific anatomical points. One [65] registered the percentage changes in the total hip (TH), femoral neck (FN), third distal radius (DR) and lumbar spine (LS) of the primates for both groups studied. The control group reported an increase in TH, FN, DR, and LS; otherwise, a more significant result was found in the Scl-AB group.

Agholme et al. (2011) [64] showed the results of µCT performed around all screw (AS), marrow surrounding (MS) and cortical surrounding (CS) in the implanted tibia (IT). There was an increased value for all parameters.

#### 3.4.2. Bone Area/Total Area (BA/TA) and Bone Volume Fraction (BVF)

Only one study [57] analyzed the BA/TA. It found better results for this parameter in the combination of ZOL-coated implant and Scl-Ab (Table S1).

All studies approached the BVF, also named Relative Bone Volume and Bone Volume per Total Volume (BV/TV). Korn et al. (2019) [57] referred to the increase in BV/TV associated with ZOL-coated implant and Scl-Ab administration. They reported a decrease in the coated implants after 4 weeks. Yu et al. (2018) [40] presented that the BFV was approximately 2 and 2.5-fold higher in the Scl-Ab than in the control group at 14 and 28 days, respectively (Table S1). Another study [35] reported a significant increase of BVF with Scl-Ab application in Sham rats. Liu et al. (2012) [62] reported values of 17.5 ± 5.8%, 31.2 ± 7.7% and 7.6 ± 2.5%, respectively, for the control, PE suspension with Scl-Ab and PE suspension with antibody vehicle administration. Virdi et al. (2012) [39] showed that, with Scl-Ab administration, BVF was, respectively, two and more than two-fold the value in the control group at 4 and 8 weeks. Omnisky et al. (2011) [63] reported BV/TV values of 27.5 ± 2.3% and 33.6 ± 2.1%, respectively, for the control and Scl-Ab group. Finally, Agholme et al. (2010) [64] reported BV/TV data which were higher when Scl-Ab was administered after screw placement (Table S1).

#### 3.4.3. Bone Thickness, Trabecular Thickness (Tb.Th) and Cortical Thickness (Ct.Th)

Virdi et al. (2012) [39] carried out the only study that reported bone thickness. This variable was greater in the Scl-Ab group than in the control at 8 weeks.

Two studies did not report information on Tb.Th. A higher Tb.Th was reported by Korn et al. (2019) [57] in both groups that received Scl.Ab treatment. Virdi et al. (2015) [35] referred that Tb.Th increased in sham rats treated with Scl-Ab.

Three studies reported values for Tb.Th in control and drug-tested groups (Table S3). Liu et al. (2012) [62] found greater Tb.Th results in PE suspension with the Scl-Ab group. Ominsky et al. (2011) [63] reported that the highest value was observed when applying Scl-Ab. Likewise, Agholme et al. (2010) [64] reported greater values in the drug-test group. However, their study demonstrated better results in the contralateral tibia than in the implanted tibia.

Ct.Th was only mentioned by Virdi et al. (2012) [39] and (2015) [35]. The first study reported that, using Scl-Ab, the peri-implant Ct.Th was greater at 8 weeks, and similarly in the contralateral femur, which was greater at 4 and 8 weeks. The second verified an increase over time with the application of Scl-Ab in OVX and sham rats; however, the effect was more pronounced in sham rats.

#### 3.4.4. Trabecular Number (Tb.N) and Trabecular Separation (Tb.Sp)

Yu et al. (2018) reported a greater Tb.N with Scl-Ab treatment than in the control group at 8 weeks [40]. Virdi et al. (2015) reported that little or no effect was observed in sham rats after Scl-Ab administration [35].

Agholme et al. (2010) [62] reported values of 1.31 ± 0.34 mm^−1^ (control), 2.01 ± 0.32 mm^−1^ (PE suspension + Scl-Ab) and 0.92 ± 0.18 mm^−1^ (PE suspension + antibody vehicle), whereas Liu et al. (2012) [64] found higher values for Tb.N in the control than in the Scl-Ab group [64] (Table S3).

Only Agholme et al. [64] and Liu et al. [62] analyzed Tb.Sp (Table S3). Liu et al. [62] reported a higher value (more spaces) in the PE suspension with the antibody group, followed by the control and PE suspension with the Scl-Ab group. Contrastingly, Agholme et al. [64] reported that Tb.Sp had higher values (more spaces) in the Scl-Ab group. 

More information on structural model index, mineralizing surface and mineral apposition rate, bone formation rate, eroded surface, osteoclast surface and cortical porosity is provided in the Supplementary Materials—Trabecular Bone.

### 3.5. Bone Remodeling

Thirteen studies [33,34,38,41,43,58,59,60,61,63,65,66,67], identified in Supplementary Tables S7ߝS15, were used to describe bone remodeling.

#### 3.5.1. Bone Mineral Density (BMD) and Bone Content

Eight studies reported BMD, of which three were RCT studies. Four studies reported the Bone Mineral Content (BMC). In animal studies, there was an increased BMD for the Scl-Ab group.

Wu et al. (2018) [59] reported that BMD in the tibia metaphysis (TM) increased 1.24, 1.25 and 1.35 times, respectively, in the Scl-Ab, PTH 1-34 and Scl-AB in PTH 1-34 groups, compared to the control, which saw a significant decrease after 12 weeks. Liu et al.’s (2018) [58] results showed that the administration of Scl-Ab led to increased BMC in OVX rats, and this was significantly higher when Scl-Ab was combined with DAB. Taut et al. (2013) [60] referred to a limited BMD increase with local Scl-Ab after 3 or 6 weeks. They also reported that systemic therapy demonstrated better results for BMD, increasing after 3 weeks and stabilizing at 6 weeks (plateau effect). However, no significant differences were reported comparing healthy rats with the test group.

Ominsky et al. [63] noticed a BMD increase of 11% and also for BMC when Scl-Ab was used; they reported significant dose-dependent increases in BMC after two months of administering the higher dose of Scl-Ab. Li et al. (2010) [38] showed the mean values of BMD and BMC for all groups (Table S7). All sites reported higher BMD and BMC with 5 mg/kg or 25 mg/kg Scl-Ab treatment compared to the control group, without significant differences between the treatment dosages. Another study [65] reported a non-significant increase in BMD with higher dosage of Scl-Ab. In contrast, volumetric BMD (vBMD) significantly increases with 30 mg/kg sc once a month of Scl-Ab (See Table S7).

For the human studies, Saag et al. (2017) [67] observed higher BMD increases in patients who received the Romosozumab therapy, mainly after 12 months. Then they transitioned to Alendronate therapy until 36 months, maintaining the BMD values. McClung et al. (2014) [41] reported the greatest BMD as associated with sc administration of Romosozumab (210 mg) once a month (Table S7). Padhi et al. (2014) [43] indicated that in each cohort significant BMD increases were observed, verified after Romosozumab treatment.

#### 3.5.2. Bone Area (BA)/Total Area (TA) and Bone Volume Fraction (BVF)

Both BA/TA and BV/TV only were reported in animal studies. The Relative Bone Area was reported by Virk et al. (2013) [61]. They referred to higher BA/TA in both studies after Scl-Ab administration compared to the control group. They also reported the highest percentage in continuous use, but that difference was insignificant between groups.

Eleven studies reported results for BVF. Liu et al.’s (2018) [58] reported restoration of the BVF levels for the treatment groups (Scl-Ab and Scl-Ab + DAB), exceeding both OVX and sham-saline groups. The other studies reported that BVF was 13.9% lower in the underloaded mandible for the saline solution group. In both test groups (Scl-Ab and Scl-Ab + DAB) after 15 weeks, the authors did not identify this evidence, noticing an increase in BVF compared to the control groups.

Wu et al. (2018) [59] reported a higher increase in BV/TV with the combined treatment of Scl-Ab and PTH 1-34 compared to the control group and other drugs tested. Taut et al. (2013) [60] saw a limited BVF increase with local application of the antibody, with the worst and a little better result, respectively, at 3 weeks and 6 weeks than the control. Virk et al. (2013) [61] showed significantly higher increases with continuous Scl-Ab treatment (Table S7).

The other five studies reported that the BV/TV was enhanced by Scl-Ab at 25 mg/kg twice a week, compared to control or lower dosages of Scl-Ab. McDonald et al. (2012) [33] showed a significant increase of BV/TV in OVX (with or without Scl-Ab treatment) compared to the application of a saline solution. At 2 and 3 weeks, they reported a reduced BV/TV in OVX without Scl-Ab treatment, while sham rats with the same treatment saw an increase; for the Scl-Ab group, they showed an improvement of the BV/TV for the same period (Table S7). Ominsky et al. (2011) reported higher gains for BV/TV with Scl-Ab treatment [63].

In Tian et al.’s (2011) study [34], the authors did not report differences in BV/TV between under- or normal-loaded sites (UL and NL, respectively) in the control group, but significant differences were found between administration dosages (5 or 25 mg/kg sc twice a week), with a dose-dependent increase. The higher the BV/TV, the higher the dosage. Li et al.’s (2010) study [38] also indicated a higher BV/TV and Tb.BV/TV with higher treatment dosage. Tian et al. (2010) [66] reported similar results for trabecular BV/TV in yellow marrow CVB (5th caudal vertebral body) and red marrow LVB (4th lumbar vertebral body) (Table S7).

#### 3.5.3. Bone Volume, Bone Height and Bone Area

Two studies studied Bone Volume [58,61], one study Bone Height [58] and three studied Bone Area [38,61,65]. In Liu et al.’s study [58], the authors reported a decrease of 38% in initial ridge bone volume 9 weeks after extraction of the right maxillary molars. With both treatments, Scl-Ab and Scl-Ab with DAB, they noticed a significant increase in bone volume 2 and 4 weeks after the beginning of treatment, with an increase of 42% and 81% in alveolar bone ridge volume, respectively, after 15 weeks. In the study reported by Virk et al. [61], the authors showed a greater bone volume with the continuous Scl-Ab treatment (Table S8).

Referring to bone height, Liu et al. [58] found a faster vertical resorption in the first 9 weeks post-extraction, with additional resorption over time, totaling a 0.41 mm height loss in the control group (saline solution). The combined treatment of Scl-Ab with DAB had the best result, with a full recovery of height loss in 9 weeks.

Li et al. [38] and Virk et al. [61] reported higher bone area after Scl-Ab treatment. In Virk et al. [61], better results were obtained with continuous treatment and with a higher treatment dose in the second [38]. Ominsky et al. [65] reported that, in Cynomolgus monkeys, the administration of 30 mg/kg of Scl-Ab led to the most prominent bone area increase (Table S8).

#### 3.5.4. Trabecular, Cortical, Medullary and Subperiosteal Areas

Trabecular, Medullary and Subperiosteal Areas (Tb.Ar, M.Ar and Tt.Ar, respectively) were only reported by Li et al. (2010) [38]. The authors reported a significantly higher Tb.Ar when applying Scl-Ab (5 mg/kg and 25 mg/kg), with a dose-dependent relation. Reported also, with 5 mg/kg and 25 mg/kg Scl-Ab application, was a greater Tt.Ar and a significantly lower M.Ar than the vehicle.

The cortical area (Ct.Ar) was studied by Li et al. (2010) and Ominsky et al. (2010) [38,65]. In general, both studies reported higher Ct.Ar values in the Scl-Ab group for all doses used (Table S9).

#### 3.5.5. Trabecular Thickness (Tb.Th) and Cortical Thickness (Ct.Th)

Seven studies reported Tb.Th [33,34,38,58,59,63,66] and four studies the Ct.Th [34,38,63,65]. Liu et al. (2018) [58] reported a higher increase of Tb.Th with Scl-Ab treatment in OVX rats than sham and OVX saline vehicle controls, as well as Wu et al.’s (2018) [59] and McDonald et al.’s (2012) [33] studies. However, Wu et al. [59] found a higher increase with the Scl-Ab and PTH 1-34 combined treatment. McDonald et al. [33] noticed a higher rise in Tb.Th with Scl-Ab treatment in OVX rats compared to sham rats (Table S10).

Ominsky et al. (2011) [63] reported that the Tb.Th was higher after Scl-Ab treatment and the following three studies reported higher Tb.Th values with the highest dose of Scl-Ab administered (25 mg/kg twice a week) at all sites compared to the control [34,38,66] (Table S12). Tian et al. (2010) [66] and (2011) [34] also verified this increase compared to baseline values (Table S10).

A higher increase in Ct.Th after Scl-Ab treatment was observed by Ominsky et al. [63]. In general, Li et al. [38] and Tian et al. [34] reported a greater increase in Ct.Th with higher doses of Scl-Ab (25 mg/kg twice a week). Conversely, Ominsky et al. [65] reported higher Ct.Th values with the lowest doses applied in Cynomolgus monkeys (Table S10).

#### 3.5.6. Structural Model Index (SMI)

Li et al.’s [38] study was the only one that reported SMI, which was significantly lower after Scl-Ab treatment, either with 5 mg/kg or 25 mg/kg doses (Table S11).

#### 3.5.7. Mineralizing Surface (MS) and Mineral Apposition Rate (MAR)

Three studies reported these two parameters [34,38,66]. Tian et al. [34] reported a higher increase with the higher dose of Scl-Ab (25 mg/kg dose twice a week) at all sites studied. Tian et al. [66] reported similar results administering 5 or 25 mg/kg of Scl-Ab twice a week. Li et al. [38] also showed higher increases in MS/BS with Scl-Ab treatment (25 mg/kg of Scl-Ab) than in the control group, with a dose-dependent increase (Table S11), but the same authors found a maximum increase (in the Ps.MAR at the tibial shaft) with 5 mg/kg compared to the control group.

#### 3.5.8. Bone Formation Rate (BFR)

Five studies reported the bone formation rate (BFR/BS) [34,38,58,65,66]. Liu et al.’s study [58] showed a significantly higher BFR/BS in basal and alveolar bone in both groups compared to the control. However, the combined treatment (Scl-Ab VI + DAB) showed a better effect on basal bone than the Scl-Ab group. Similar results were reported by Li et al. [38], Tian et al. [66], and Tian et al. [34] (Table S11). Ominsky et al. [65] reported a significant increase in Ec.BFR/BS and a non-significant increase in Ps.BFR/BS, with the administration of 30 mg/kg of Scl-Ab, once a month.

#### 3.5.9. Bone Formation/Resorption Biomarkers

Three biomarkers involved in bone formation were identified: bone-specific alkaline phosphatase (BSAP), osteocalcin and procollagen type 1 N-terminal Pro-peptide (P1NP). The biomarkers found for bone resorption found were serum C-telopeptide (sCTX), C-terminal telopeptides of type I collagen (CTX-1), β-isomer of C-terminal telopeptides of type I collagen (β-CTX) and tartrate-resistant acid phosphatase 5b (TRACP-5b).

Liu et al. (2018) [58] reported a higher increase of BSAP with Scl-Ab alone or Scl-Ab with DAB treatments compared to Sham and OVX saline controls. There was also a higher enhancement of osteocalcin and P1NP with the administration of 25 mg/kg of Scl-Ab twice a week than with saline solution in the intact or extracted mandible (Table S13). The same study reported a decrease of TRACP-5b biomarker with both treatments tested (Scl-Ab and Scl-Ab + DAb) compared to the control, with higher expression for the combined treatment (Table S14).

Wu et al. (2018) [59] showed an increase of osteocalcin and P1NP when administering Scl-Ab alone and Scl-Ab with PTH 1-34 at 12 weeks (Table S13). No differences were reported among all groups for the CTX-1 resorption marker (Table S14). Similarly, Virk et al. (2013) [61] had a significantly higher increase of osteocalcin and P1NP, respectively, at 6 and 12 weeks; Ominsky et al. (2011) [63] also reported more significant increases in osteocalcin and P1NP with Scl-Ab treatment and, likewise, Taut et al. (2013) [60] obtained an increase in osteocalcin and P1NP 3 weeks after the beginning of treatment. Therefore, only osteocalcin had increased after six weeks, but there were no differences in P1NP (Table S13). The authors did not report changes in TRACP-5b compared to vehicle-EP control at 6 weeks (Table S14).

Li et al. 2010 [38] referred to increases in osteocalcin biomarker levels one week after the Scl-Ab therapy, with both doses tested maintaining greater values over time. Moreover, a dose-dependent effect was found, with greater values identified for 25 mg/kg of Scl-Ab twice a week (Table S13). For the CTX-1 biomarker, the same authors did not report significant effects with Scl-Ab. Ominsky et al. [65] reported similar information in the CTX serum biomarker in Cynomolgus monkeys.

For the clinical studies [41,43,67], Saag et al. (2017) [67] found an increase in P1NP levels (after 12 months) with the administration of Romosozumab. Otherwise, after this period and with the transition to Alendronate therapy, the P1NP levels decreased (Table S13). A decrease of βCTX levels was also noticed at 12 months, coincident with the end of Romosozumab treatment and was maintained until 36 months (after transition for Alendronate). Compared to treatment made only with Alendronate (at 12 months), the decrease was greater with Romosozumab (Table S14).

In McClung et al.’s (2014) [41] study, the authors verified increased biomarker levels after 1 week of using Scl-Ab. Nevertheless, after 1 month, a decrease or lower values were reported, which varied according to doses and biomarkers (BSAP, osteocalcin, or P1NP). The teriparatide seemed to increase bone formation biomarkers over time after the third month (Table S13). In all groups that received Romosozumab, a decrease in the values of the βCTX biomarker was observed mainly in the first week. With the administration of Romosozumab monthly (210 mg/kg once every 3 months), they reported that the values remained below the baseline data after 12 months (Table S14).

The last clinical study, by Padhi et al. (2014) [43], reported increased levels for P1NP, BSAP and osteocalcin biomarker, with the administration of 2mg/kg of Romosozumab every 2 weeks in women and 3mg/kg of Romosozumab every 4 weeks in men (Table S13). They also showed decreases from baseline for sCTX with Romosozumab administration compared to placebo control (Table S14).

#### 3.5.10. Bone Strength Endpoints

##### Maximum Load

Two studies reported the maximum load (Li et al., 2010 [38] and Wu et al., 2018 [59]). Wu et al. noticed significant increases with Scl-Ab, PTH 1-34 and Scl-Ab with PTH 1-34, compared to the vehicle, but non-significant differences among them after 12 weeks of treatment. Li et al. reported a significant increase in maximum load with the administration of Scl-Ab compared to the control. A dose-dependent increase was noted, with a significantly higher result obtained with 25 mg/kg twice a week (Table S15).

##### Stiffness

Five studies reported qualitative or quantitative information for stiffness [38,59,61,65]. Wu et al. (2018) [59] reported a significant increase in stiffness with Scl-Ab, PTH 1-34 and Scl-Ab + PTH 1-34, compared to the vehicle. They also showed significant results with the administration of Scl-Ab with PTH 1-34 compared to the other tested groups after 12 weeks. One of the studies (Virk et al., 2013) [61] only observed that, after 6 weeks of treatment, they verified a significantly higher increase when contrasted with the PBS control.

Li et al. (2010) [38] referred that the stiffness was higher with a higher treatment dose (25 mg/kg of Scl-Ab twice a week). They described a higher stiffness with a dosage of 5 mg/kg twice a week compared to vehicle or Scl-Ab 25 mg/kg. Ominsky et al. (2011) [63] reported increased stiffness in both fractured and intact femurs with Scl-Ab therapy compared to the control group (Table S15).

Similar results were reported by Ominsky et al. (2010) [65], with greater stiffness with 30 mg/kg of Scl-Ab once a month compared to the control group. With lower doses, a non-significant decrease of values was reported compared to vehicle.

### 3.6. Incidents Found

Adverse events were only reported in RCT studies (Saag et al., 2017 [66]; McClung et al., 2014 [41]; Padhi et al., 2014 [43]); Saag et al.’s study [67] reported adverse events and deaths with both Alendronate or Romosozumab administration. They also noticed some serious adjudicated cardiac events, such as cardiac ischemic and cerebrovascular events, heart failure, noncoronary revascularization, or peripheral vascular ischemic event not requiring revascularization. In general, the Romosozumab group had higher relation with these events (Table 3).

McClung et al. 2014 [41] referred that the incidence of adverse events was similar between the placebo and Romosozumab group, but no serious adverse event was associated with the treatment. The pain at the injection site was greater with Romosozumab treatment compared to placebo, but no relation was mentioned regarding the dose administered.

Padhi et al. 2014 [43] reported that almost all participants that received a placebo or Romosozumab had at least one adverse event. They only reported the main adverse events described in Table 3.

## 4. Discussion

Owing to the growing number of implants currently placed and the benefits the patient could obtain with a faster functional and esthetic rehabilitation, this study reviewed the literature to verify the influence of local or systemic administration of Scl-Ab on dental/orthopedic implants osseointegration and stimulation of bone remodeling. This can help identify strategies to improve the osseointegration process and new bone formation. 

### 4.1. Osseointegration of Implants

It was verified that few articles had studied the Scl-Ab effect as a treatment for implant osseointegration and only one [40] verified that Scl-Ab can improve this phenomenon. In general, BIC was higher when the Scl-Ab treatment was performed. This fact was corroborated by Virdi et al. (2012) [39] and Yu et al. (2018) [40], who reached a greater BIC with Scl-Ab at 28 days. Otherwise, Korn et al. (2019) [57] partially agreed with this information. They reported a more significant increase when performing Scl-Ab treatment but decreased BIC after 4 weeks using sandblasted and thermally acid-etched surfaces. This controversial finding must be further investigated because, typically, the BIC is higher when implants have the surface treated.

In general, the Scl-Ab therapies improved the proprieties of implant fixation, providing a significant increase in the newly formed bone [39,62]. An augmented fixation strength was associated with Scl-Ab treatment [35], with a higher enhancement in sham rats. 

### 4.2. Bone Mineral Density (BMD)

We noted that Scl-Ab treatment positively affected BMD around the implants placed [57,64], also showing a positive systemic effect [64]. Ominsky et al. (2011) [63] also reported greater values in different sites. In contrast, Yu et al. (2018) [40] found no differences in BMD between Scl-Ab and control.

To evaluate the effect of Scl-Ab in bone remodeling, it was verified that eight studies included a positive impact on BMD, promoting its increase after administration in animal and human studies. Wu et al. (2018) [59] reported an increase in BMD with the Scl-Ab, but a higher effect was noticed with the combination of Scl-Ab and PTH 1-34. Li et al. (2010) [38] had an increase in BMD with either dose tested. Ominsky et al. (2011) [63] reported a rise of 11% in BMD.

Taut et al. (2013) [60] also reported that the systemic Scl-Ab III treatment trend increased the BMD. On the other hand, the improvement was minimal in the case of local administration. All the evidence corroborates that BMD is increased by Scl-Ab treatment. Even though Ominsky et al.’s (2010) results had a greater increase of volumetric BMD with greater doses in cynomolgus monkeys, they reported that the rise in the BMD area was not significant with the same doses.

McClung et al. (2014) [41], Padhi et al. (2014) [43], and Saag et al. (2017) [67] reported increased BMD with Romosozumab therapy. McClung et al. (2014) [41] also reported the highest growth, administrating 210 mg once a month.

It was noted that the BMC also increased even with different Scl-Ab therapies. Liu et al. (2018) [58] noted a higher effect expression with the combined treatment with Scl-Ab VI and DAB. Moreover, Li et al. (2010) [38] and Ominsky et al. (2010) [65] reported a more significant Scl-Ab effect when used in higher doses.

### 4.3. Bone Area (BA)/Total Area (TA) and Bone Volume (BV)/Total Volume (TV)

In Virk et al.’s study [61], authors reported an increase in BA/TA, with a higher result when there was continuous therapy. The BA also increased significantly with the Scl-Ab treatment, with higher effects associated with a continuous period of treatment [61] and higher doses [36,65].

Similar to the increase in BVF, some studies also reported an augmentation in BV. Liu et al. (2018) [58] recognized an increased BV using only Scl-Ab VI; otherwise, the combination of Scl-Ab VI and DAB caused an improved result in the alveolar ridge volume. Virk et al. (2013) [61] referred to higher increases with continuous treatment.

In general, we identified the increase of BVF around the implant after Scl-Ab therapy. This information is supported by Agholme et al. (2010) [64], Liu et al. (2012) [62], Virdi et al. (2012) [39], Virdi et al. (2015) [35] and Korn et al. (2019) [57]. Increases in systemic BFV were also identified (Ominsky et al., 2011) with a higher value in FN and with a higher BV/TV [64].

In general, the BV/TV increased after the Scl-Ab treatment. However, some particularities were noted in some studies. Liu et al. (2018) [58] reported increased BV/TV with Scl-Ab VI treatment alone and combined with DAB. Similar results were identified by Wu et al. (2018) [59], reporting a greater increase in the therapy with Scl-Ab III and PTH. Regarding local use, Taut et al. (2013) [60] showed some effects on the BV/TV improvement compared to systemic administration. Tian et al.’s (2011) [34] study concluded on higher increases in the higher doses after comparing 5 and 25 mg/kg twice a week in rats.

### 4.4. Cortical and Trabecular Analysis

A higher trabecular bone (Tb) and cortical bone thickness (Th) was observed after Scl-Ab therapy. There was a systemic increase in Tb.Th after Scl-Ab therapy (Agholme et al. [64], Ominsky et al. [63]).

Several studies in the literature corroborated the information on the increase in Tb.Th around implants. Korn et al.’s (2019) [57] study reported the enhancement of Tb.Th with both implant surfaces analyzed; Liu et al. (2012) [62] referred to this association with a higher value of Scl-Ab; and Agholme et al. (2010) [64] also noticed a better result. Similarly, the cortical (Ct) thickness improved around implants after Scl-Ab treatment. This fact is supported by Virdi et al. (2012 and 2015). Generally, there were better results matched with higher therapeutic doses (25 mg/kg twice a week) [34,38,63]. However, a different effect was obtained by only one study [65], with a higher increase in Ct.Th with a lower dose.

There was an increase in Tb.Th in those studies which tested the effect of Scl-Ab in OVX rats [33,58,59]. The results were higher in OVX rats, compared to the vehicles used in OVX rats and vehicle and drug in Sham rats. Other studies compared the effect of higher and lower doses of Scl-Ab [34,38,66], showing, in general, a higher increase with 25 mg/kg twice a week, with some particularity in Tian et al.’s (2011) [34], who reported a higher increase in the tibia. 

The trabecular number (Tb.N) increased with Scl-Ab therapy, but divergent results were found in the literature. Yu et al. (2018) [40] reported a greater Tb.N at 8 weeks around dental implants, Liu et al. (2012) [62] reported a higher increase after Scl-Ab application (25 mg/kg twice a week) and Wu et al. (2018) [59] reported elevated results with all treatment options studied, with higher increases observed for the combination of Scl-Ab with PTH 1-34. On the other hand, different results were reported by other authors. One mentioned that the Scl-Ab treatment had little or no effect in Tb.N [35], whereas the other noticed higher values in the control group [64]. Tian et al. (2010) [66], Tian et al. (2011) [34] and McDonald et al. (2012) [33] also showed different results.

The trabecular separation (Tb.Sp) decreased with the Scl-Ab administration, as referred by Agholme et al. (2010) [64], Li et al. (2010) [38], Tian et al. (2010) [66] and Wu et al. (2018) [59]. However, we identified different results for Tb.Sp. Liu et al. (2012) [62] reported the lowest Tb.Sp for the Scl-Ab group; Tian et al. (2011) [34] reported decreased values with the administration of 5 and 25 mg/kg of Scl-Ab.

### 4.5. Bone Formation Rate (BFR)

The BFR increased after the Scl-Ab treatment compared to the control groups. All the studies [34,38,58,66] performed in rats had significantly higher BFR results for the Scl-Ab treatment. There was also an increase in the results of two studies [35,62] observing the BFR around implants, and in one reporting the systemic increase [63].

There were particularities to each study. Liu et al. (2018) [58] reported a significantly higher increase in the basal bone with Scl-Ab with DAB, compared to the Scl-Ab group. Tian et al. (2010) [66], Li et al. (2010) [38], and Tian et al. (2011) [34] reported more significant increases in BFR/BS with higher Scl-Ab treatment doses and Ominsky et al. (2010) [65] obtained a substantial increase in BFR/BS with 30 mg/kg administration once a month of Scl-Ab.

The local rise in BFR/BS was reported by Liu et al. (2012) [62] and Virdi et al. (2015) [35]. Otherwise, Virdi et al. [35] noticed a decreasing result over time. Only Ominsky et al. (2011) [63] reported systemic effects along with BFR/BS increase over time.

In general, some studies [34,38,66] showed similar results in mineral apposition rate, with better outcomes for administering 25 mg/kg of Scl-Ab, twice a week, but Tian et al. (2010) [66] reported no differences in dose administration.

### 4.6. Bone Strength and Stiffness

Generally, the Scl-Ab treatment provided an increase in bone strength and stiffness. We identified increased resistance in maximum load in Wu et al.’s (2018) [59] and Li et al.’s (2010) [38] studies. Li et al. (2010) [38] reported that the increased strength in maximum load was related to the dose of Scl-Ab therapy administered.

Moreover, we noted that stiffness and energy to fail significantly increased with Scl-Ab treatment [38,59,61,63,65]. Wu et al. (2018) [59] referred to a greater increase in stiffness with the association of Scl-Ab with PTH 1-34. Li et al. (2010) [38] reported some contrasting information for stiffness. They referred to a higher stiffness related to higher treatment doses but reported a more significant effect in stiffness with lower doses in specific sites. However, they reported an increase with higher dose administration at energy necessary to fail. Similarly, Ominsky et al. (2010) [65] reported that increased stiffness and energy to fail were obtained with a higher dose of Scl-Ab treatment. 

The high values for stiffness were supported by Virdi et al. (2015) [35], who reported a significant increase over time, with better results in sham rats. On the other hand, Virdi et al. (2012) [39] demonstrated a considerable increase over time for the Scl-Ab group, with apparent results after eight weeks. Ominsky et al. (2011) [63] reported an increase in torsional stiffness of 48%. In contrast, Liu et al. (2012) [62] found the highest stiffness value in the control group.

### 4.7. Bone Biomarkers

An increase in the biomarkers associated with bone formation was observed after the beginning of treatment. Liu et al. (2018) [58] reported a rise in BSAP for Scl-Ab and Scl-Ab with DAB and an increase in osteocalcin and P1NP with Scl-Ab. Similar results were obtained by Ominsky et al. (2011) [63], Taut et al. (2013) [60], Virk et al. (2013) [61]. Wu et al. (2018) [59] reported greater increases in osteocalcin and P1NP with the administration of Scl-Ab and a greater increase was reported [59] using Scl-Ab with PTH 1-34.

A decrease in the biomarkers linked to bone resorption was also observed. Liu et al. (2018) [58] reported a reduction in TRACP-5b for both groups studied (Scl-Ab and Scl-Ab + DAB), with a higher effect in the latter. Contrastingly, Li et al. (2010) [38] and Wu et al. (2018) [59] did not report differences in the biomarkers between Scl-Ab and the control group. Ominsky et al. (2010) [65] and Taut et al. (2013) [60] reported that no differences were found in TRACP-5b and CTX serum, respectively.

The disagreement among studies on this topic may have many origins. Different doses and periods of Scl-Ab application can be cited, causing different biological responses, different types of animals and protocols and divergent periods of observation. This topic (bone biomarkers) must still be investigated more deeply.

### 4.8. Study Applicability

This review sought to improve the understanding of the effects of antisclerostin on bone formation/remodeling and osseointegration. The general result supports increased bone formation, promoting and accelerating peri-implant osseointegration when using Scl-Ab. This fact enables concomitant osseous stimulation and inhibition of bone resorption [68].

Therefore, more solid and robust studies must be developed, mainly for evaluating bone formation around implants, which has limited literature in support. In addition, the systemic risks must be better analyzed when the drug is administered, mainly cardiovascular damage/events [64], which may be verified in future studies.

### 4.9. Limitations of the Study

This systematic review does present some limitations. These are related to the low number of human studies identified; the high divergence in the model of the studies (animal or human models); the variance verified in the type of Scl-Ab administered and doses of administration to the treatment group; and the lack of quantitative reference or only few quantitative values for the parameters analyzed. Moreover, many articles merely reported qualitative information.

## 5. Conclusions

Within the limitations of this narrative study and carefully observing all data, due to the limited number of studies included, more studies must be carried out to better evaluate the antisclerostin action on the osseointegration of dental/orthopedic implants. Otherwise, it can accelerate and stimulate the bone remodeling and neoformation.

## Figures and Tables

**Table 1:** Main characteristics of selected articles—part I.

Author	Year	Country (Study)	Study Center	Study Type	Species	Sample Size (Initial)		Sample Size (Final)		Age (Mean)	Gender
Korn et al. [57]	2019	Switzerland	Basel-Stadt Cantonal Veterinary Office	Animal	Wistar rats	128		124		6-month-old	female
Liu et al. [58]	2018	USA	-	Animal	Sprague-Dawley rats	50	40 OVX ^a^	50	40 OVX ^a^	6-month-old	female
							10 Sham ^b^		10 Sham ^b^		
					Sprague-Dawley rats	45		45		8-month-old	male
Wu et al. [59]	2018	China	-	Animal	Sprague-Dawley rats	50	5 Sham	40 OVX		3-month-old	female
							5 OVX				
							40 OVX				
Yu et al. [40]	2018	USA	University of Michigan	Animal	Sprague-Dawley rats	60		60		8-month-old	male
Virdi et al. [35]	2015	USA	-	Animal	Sprague-Dawley rats	144	72 OVX ^a^	142	71 OVX ^a^	4.5-month-old	female
							72 Sham ^b^		71 Sham ^b^		
Taut et al. [60]	2013	USA	-	Animal	Sprague-Dawley rats	69		69		9–10-week-old	male
Virk et al. [61]	2013	USA	University of Connecticut Health Center	Animal	Lewis rats	72		72		14-week-old	male
					Lewis rats	30		30		14-week-old	male
Liu et al. [62]	2012	USA	-	Animal	Sprague-Dawley rats	36		36		-	male
McDonald et al. [33]	2012	Australia	-	Animal	Sprague-Dawley rats	132	66 Sham ^b^	127		-	female
							66 OVX ^a^				
Virdi et al. [39]	2012	USA	-	Animal	Sprague-Dawley rats	90		88		6-month-old	male
Ominsky et al. [63]	2011	Canada	Charles River Laboratories	Animal	Sprague-Dawley rats	35		32		7–7.5-month-old	male
					Cynomolgus monkeys	43		29		4–5 years old	male
Tian et al. [34]	2011	USA	University of Utah	Animal	Sprague-Dawley rats	67		67		10-month-old	female
Agholme et al. [64]	2010	Sweden	-	Animal	Sprague-Dawley rats	68		64		10-month-old	male
Li et al. [38]	2010	USA	-	Animal	Sprague-Dawley rats	28		26		16-month-old	male
Ominsky et al. [65]	2010	Canada	Charles River Laboratories	Animal	Cynomolgus monkeys	12		12		3–5 years old	female
Tian et al. [66]	2010	USA	University of Utah	Animal	Sprague-Dawley rats	32		32		10-month-old	female
Saag et al. [67]	2017	-	Multicenter international	RCT, ph.3 ^c^	Human	4093		3150		55–90 years old	women
McClung et al. [41]	2014	-	Multicenter international (28 centers)	RCT, ph.2 ^d^	Human	419		383		55–89 years old	women
Padhi et al. [43]	2014	USA	4 centers	RCT ^e^	Human	48	32 women	46	31 women	45–80 years old	Postmenopausal women & men
							16 men		15 men		

^a^ OVX—Ovarectomized rats; ^b^ Sham—Sham-ovarectomized rats; ^c^ RCT, ph 3—Phase 3, randomized, double-blind trial; ^d^ RCT, ph 2—Phase 2, randomized, placebo-controlled; ^e^ RCT—Randomized, double-blind, placebo-controlled. Green background=clinical studies.

**Table 2:** Main characteristics of selected articles—part II.

	Sample Size (Initial)		Sample Size (Final)		Control	Drug (Name)	Administration Route	Dosage (Unit)	Period of Treatment	Implant
Korn et al. (2019) [57]	128		124		non antibody applied	sclerostin antibody	intravenous	100 mg/kg once week	2 or 4 weeks	Reference-coated implant
										ZOL-coated implant
Liu et al. (2018) [58]	50	40 OVX	50	40 OVX	saline solution	Scl-Ab VI	subcutaneous	18.2 mg/kg twice week	5 weeks	not placed
						Scl-Ab VI + DAB ^d^		18.1 mg/kg + 18.1 mg/kg twice week		
		10 Sham		10 Sham		-		-		
	45		45		saline solution	Scl-Ab VI	subcutaneous	25 mg/kg twice week	15 weeks	not placed
						Scl-Ab VI + DAB ^d^		25 mg/kg + 25 mg/kg twice week		
Wu et al. (2018) [59]	50	5 Sham	-		-	-	-	-	-	-
		5 OVX	-		-	-	-	-	-	-
		40 OVX	40 OVX		vehicle	Scl-Ab ^e^	subcutaneous	25 mg/kg twice week	12 weeks	not placed
						PTH 1-34 ^f^		60 μg/kg thrice week		
						Scl-Ab ^e^ + PTH 1-34 ^f^		25 mg/kg twice week + 60 μg/kg thrice week		
Yu et al. (2018) [40]	60		60		PBS ^a^	Scl-Ab	subcutaneous	25 mg/kg	10, 14 or 28 days	cp-Ti, solid cylinder with titanium plasma-sprayed surface implant
Virdi et al. (2015) [35]	144	72 OVX	142	71 OVX	vehicle	Scl-Ab III ^g^	subcutaneous	25 mg/kg twice week	4, 8 or 12 weeks	cp-Ti with dual acid-etched surface implant
		72 Sham		71 Sham						
Taut et al. (2013) [60]	69		69		EP ^b^: vehicle healthy: PBS	EP ^b^: Scl-Ab III ^g^	subcutaneous	25 mg/kg twice week	3 or 6 weeks	not placed
							locally	15 μL of 35.6 mg/mL solution ^m^ twice week		
Virk et al. (2013) [61]	72		72		PBS ^a^	Scl-Ab III ^g^	subcutaneous	25 mg/kg twice week	0–12 weeks ^n^	not placed
									0–2 weeks ^o^	
									2–4 weeks ^p^	
	30		30		PBS ^a^	Scl-Ab III ^g^	-	25 mg/kg	12 weeks	not placed
Liu et al. (2012) [62]	36		36		particle vehicle + antibody vehicle	PE suspension ^h^ + antibody vehicle	intraarticular + subcutaneous	50 μL once week + vehicle twice week	12 weeks	titanium rods, dual acid-etched surface
						PE suspension ^h^ + Scl-Ab III ^g^		50 µL once week + 25 mg/kg twice week		
McDonald et al. (2012) [33]	132	66 Sham	127		saline solution	Scl-Ab III ^g^	subcutaneous	25 mg/kg twice week	1, 2 or 3 weeks	not placed
		66 OVX								
Virdi et al. (2012) [39]	90		88		saline solution	Scl-Ab ^i^	subcutaneous	25 mg/kg	2, 4 or 8 weeks	cp-Ti with dual acid-etched surface implant
Ominsky et al. (2011) [63]	35		32		vehicle	Scl-Ab III ^g^	subcutaneous	25 mg/kg twice week	7 weeks	not placed
	43		29		vehicle	Scl-Ab V ^j^	subcutaneous	30 mg/kg every 2 weeks	10 weeks	stainless steel K-wire
Tian et al. (2011) [34]	67		67		saline solution	Scl-Ab III ^g^	subcutaneous	5 mg/kg twice week	4 weeks	not placed
								25 mg/kg twice week		
Agholme et al. (2010) [64]	68		64		saline solution	Scl-Ab III ^g^	subcutaneous	25 mg/kg twice weeks	2 or 4 weeks	stainless steel screws (mechanical tests); PMMA screws (μCT)
Li et al. (2010) [38]	28		26		vehicle	Scl-Ab III ^g^	subcutaneous	25 mg/kg twice week	5 weeks	not placed
								5 mg/kg twice week		
Ominsky et al. (2010) [65]	12		12		vehicle	Scl-Ab IV ^k^	subcutaneous	3 mg/kg once month	29 days	not placed
								10 mg/kg once month		
								30 mg/kg once month		
Tian et al. (2010) [66]	32		32		saline solution	Scl-Ab III ^g^	subcutaneous	5 mg/kg twice week	4 weeks	not placed
								25 mg/kg twice week		
Saag et al. (2017) [67]	4093		3150		Alendronate ^c^ → alendronate ^c^	Romosozumab ^l^ → alendronate ^c^	subcutaneous → oral	210 mg once month → 70 mg once week	0-12 months ^q^ → 12–36 months ^r^	not placed
McClung et al. (2014) [41]	419		383		placebo	romosozumab	subcutaneous	140 mg every 3 months	12 months	not placed
								210 mg every 3 months		
								70 mg once month		
								140 mg once month		
								210 mg once month		
						alendronate	oral	70 mg once week		
						teriparatide	subcutaneous	20 μg once day		
Padhi et al. (2014) [43]	48	32 women	46	31 women	placebo	romosozumab	subcutaneous	1 mg/kg every 2 weeks	12 weeks	not placed
								2 mg/kg every 4 weeks		
								2 mg/kg every 2 weeks		
								3 mg/kg every 4 weeks		
		16 men		15 men		romosozumab	subcutaneous	1 mg/kg every 2 weeks		
								3 mg/kg every 4 weeks		

^a^ PBS—Phosphate-buffered saline solution; ^b^ EP—Experimental periodontitis model; ^c^ Alendronate—Alendronate, Merck; ^d^ DAB—DKK1 Antibody; ^e^ Scl-Ab—Sclerostin antibody, Amgen, Thousand Oaks, California; ^f^ PTH 1-34—human Parathyroid Hormone 1-34, Bachem, Torrance, California; ^g^ Scl-Ab III—Sclerostin antibody III (murine sclerostin antibody), Amgen and UCB Pharma, Thousand Oaks, California; ^h^ PE suspension—Polyethylene particle suspension; ^i^ Scl-Ab—Murine sclerostin antibody, Amgen, Thousand Oaks, California; ^j^ Scl-Ab V—Humanized sclerostin antibody, Amgen and UCB Pharma; ^k^ Scl-Ab IV—Humanized sclerostin-neutralizing monoclonal antibody; ^l^ Romosozumab—AMG 785/CDP7851, Amgen and UCB Pharma; ^m^ 15 μL of 35.6 mg/mL solution—5 μL of 35.6 mg/mL of solution per site twice a week, giving a total of 15 μL per animal per treatment session ^n^ 0–12 weeks—continuous group; ^o^ 0–2 weeks—early group; ^p^ 2–4 weeks—delayed group; ^q^ 0–12 months—Double blind period; ^r^ 12–36 months—Open label period. Green background = clinical studies.

**Table 3:** Incidents found.

	**Saag et al. (2017)** [67]				**McClung et al. (2014)** [41]								**Padhi et al. (2014)** [43]						
**Drug/Control**	**Double-Blind Period**		**Primary Analysis Period**		**Placebo**	**Alendronate**	**Teraparatide**	**Romosozumab**					**Placebo**	**Romosozumab**					
	**Alendronate → Alendronate**	**Romosozumab → Alendronate**	**Alendronate → Alendronate**	**Romosozumab → Alendronate**										**Women**				**Men**	
**Dosage** **(unit)**	70 mg → 70 mg once week	210 mg once month → 70 mg once week	70 mg → 70mg once week	210 mg once month → 70 mg once week	-	70 mg once week	20 μg once day	140 mg every 3 moths	210 mg every 3 months	70 mg once month	140 mg once month	210 mg once month	-	1 mg/kg every 2 weeks	2 mg/kg every 4 weeks	2 mg/kg every 2 weeks	3 mg/kg every 4 weeks	1 mg/kg every 2 weeks	3 mg/kg every 4 weeks
**Number of participants**	2014	2040	2014	2040	50	51	54	53	53	50	48	51	12	6	6	6	6	6	6
**Adverse Events**	1584 (78.6%)	1544 (75.7%)	1784 (88.6%)	1766 (86.6%)	45 (90%)	44 (86.3%)	37 (68.5%)	43 (81.1%)	46 (86.8%)	48 (96%)	42 (87.5%)	42 (87.4%)	10 (83%)	6 (100%)	6 (100%)	6 (100%)	5 (83%)	5 (83%)	5 (83%)
**Headache**	-	-	-	-	8 (16%)	4 (7.8%)	3 (5.6%)	7 (13.2%)	3 (5.7%)	4 (8.0%)	3 (6.3%)	5 (9.8%)	4 (33%)	1 (17%)	1 (17%)	1 (17%)	2 (33%)	3 (50%)	2 (33%)
**Upper respiratory tract infection**	-	-	-	-	-	-	-	-	-	-	-	-	1 (8%)	3 (50%)	1 (17%)	2 (33%)	0	2 (33%)	0
**Arthralgia**	-	-	-	-	4 (8%)	5 (9.8%)	5 (9.3%)	19 (17%)	5 (9.4%)	8 (16%)	6 (12.5%)	3 (5.9%)	2 (17%)	0	2 (33%)	0	1 (17%)	1 (17%)	1 (17%)
**Pain in Extremity**	-	-	-	-	2 (4%)	2 (3.9%)	5 (9.3%)	7 (13.2%)	3 (5.7%)	10 (20%)	5 (10.4%)	6 (11.8%)	2 (17%)	0	2 (33%)	0	1 (17%)	0	1 (17%)
**Abdominal pain**	-	-	-	-	-	-	-	-	-	-	-	-	1 (8%)	0	1 (17%)	1 (17%)	0	1 (17%)	0
**Back pain**	228 (11.3%)	186 (9.1%)	393 (19.5%)	329 (16.1%)	3 (6.0%)	5 (9.8%)	3 (5.6%)	4 (7.5%)	7 (13.2%)	5 (10%)	7 (14.6%)	3 (5.9%)	2 (17%)	3 (50%)	0	0	0	0	0
**Injection site pain**	-	-	-	-	0	0	0	2 (3.8%)	4 (7.5%)	3 (6%)	4 (8.3%)	3 (5.9%)	0	0	0	2 (33%)	0	1 (17%)	0
**Injection site reaction**	53 (2.6%)	90 (4.4%)	53 (2.6%)	90 (4.4%)	-	-	-	-	-	-	-	-	0	0	0	1 (17%)	1 (17%)	0	1 (17%)
**Lymphadenopathy**	-	-	-	-	-	-	-	-	-	-	-	-	0	1 (17%)	0	1 (17%)	1 (17%)	0	0
**Nasopharyngitis**	218 (10.8%)	213 (10.4%)	373 (18.5%)	363 (17.8%)	7 (14%)	3 (5.9%)	4 (7.4%)	10 (18.9%)	5 (9.4%)	19 (38.0%)	13 (27.1%)	8 (15.7%)	-	-	-	-	-	-	-
**Gastroenteritis**	-	-	-	-	3 (6%)	2 (3.9%)	1 (1.9%)	2 (3.8%)	5 (9.4%)	3 (6%)	4 (8.3%)	8 (15.7%)	-	-	-	-	-	-	-
**Cough**	-	-	-	-	2 (4%)	4 (7.8%)	0	3 (5.7%)	1 (1.9%)	8 (16%)	4 (8.3%)	4 (7.8%)	-	-	-	-	-	-	-
**Constipation**	-	-	-	-	2 (4%)	3 (5.9%)	2 (3.7%)	2 (3.8%)	5 (9.4%)	4 (8%)	4 (8.3%)	2 (3.9%)	-	-	-	-	-	-	-
**Bronchitis**	-	-	-	-	2 (4%)	1 (2%)	2 (3.7%)	5 (9.4%)	1 (1.9%)	5 (10%)	3 (6.3%)	2 (3.9%)	-	-	-	-	-	-	-
**Urinary tract infection**	-	-	-	-	0	4 (7.8%)	3 (5.6%)	3 (5.7%)	5 (9.4%)	0	3 (6.3%)	5 (9.8%)	-	-	-	-	-	-	-
**Fatigue**	-	-	-	-	2 (4.0%)	2 (3.9%)	0	1 (1.9%)	1 (1.9%)	5 (10%)	5 (10.4%)	2 (3.9%)	-	-	-	-	-	-	-
**Musculoskeletal pain**	-	-	-	-	2 (4.0%)	2 (3.9%)	2 (3.7%)	3 (5.7%)	3 (5.7%)	4 (8%)	2 (4.2%)	1 (2%)	-	-	-	-	-	-	-
**Adjudicated serious cardiovascular event**	38 (1.9%)	50 (2.5%)	122 (6.1%)	133 (6.5%)	-	-	-	-	-	-	-	-	-	-	-	-	-	-	-
**Cardiac ischemic event**	6 (0.3%)	16 (0.8%)	20 (1.0%)	30 (1.5%)	-	-	-	-	-	-	-	-	-	-	-	-	-	-	-
**Cerebrovascular event**	7 (0.3%)	16 (0.8%)	27 (1.3%)	45 (2.2%)	-	-	-	-	-	-	-	-	-	-	-	-	-	-	-
**Heart failure**	8 (0.4%)	4 (0.2%)	23 (1.1%)	12 (0.6%)	-	-	-	-	-	-	-	-	-	-	-	-	-	-	-
**Noncoronary revascularization**	5 (0.2%)	3 (0.1%)	10 (0.5%)	6 (0.3%)	-	-	-	-	-	-	-	-	-	-	-	-	-	-	-
**Peripheral vascular ischemic event not requiring revascularization**	2 (<0.1%)	0	5 (0.2%)	2 (<0.1%)	-	-	-	-	-	-	-	-	-	-	-	-	-	-	-
**Osteoarthritis**	146 (7.2%)	138 (6.8%)	268 (13.3%)	247 (12.2%)	-	-	-	-	-	-	-	-	-	-	-	-	-	-	-
**Hypersensitivity**	118 (5.9%)	122 (6%)	185 (9.2%)	205 (10%)	-	-	-	-	-	-	-	-	-	-	-	-	-	-	-
**Cancer**	28 (1.4%)	31 (1.5%)	85 (4.2%)	84 (4.1%)	-	-	-	-	-	-	-	-	-	-	-	-	-	-	-
**Hyperostosis**	12 (0.6%)	2 (<0.1%)	27 (1.3%)	23 (1.1%)	-	-	-	-	-	-	-	-	-	-	-	-	-	-	-
**Hypocalcemia**	1 (<0.1%)	1 (<0.1%)	1 (<0.1%)	4 (0.2%)	-	-	-	-	-	-	-	-	-	-	-	-	-	-	-
**Atypical femoral fracture**	0	0	4 (0.2%)	2 (<0.1%)	-	-	-	-	-	-	-	-	-	-	-	-	-	-	-
**Osteonecrosis of the Jaw**	0	0	1 (<0.1%)	1 (<0.1%)	-	-	-	-	-	-	-	-	-	-	-	-	-	-	-
**Serious adverse event**	278 (10.8%)	262 (12.8%)	605 (30.0%)	586 (28.7%)	7 (14%)	4 (7.8%)	5 (9.3%)	4 (7.5%)	2 (3.8%)	5 (10%)	1 (2.1%)	5 (9.8%)	-	-	-	-	-	-	-
**Fatal adverse events (Deaths)**	21 (1.0%)	30 (1.5%)	90 (4.5%)	90 (4.4%)	1 (2%)	0	0	0	0	1 (2%)	0	0	-	-	-	-	-	-	-

## Supplementary Materials

The following supporting information can be downloaded at: https://www.mdpi.com/article/10.3390/jcm12041294/s1, Table S1: Osseointegration/Bone formation parameters-Part I; Table S2: Osseointegration/Bone formation parameters–Part II.; Table S3: Osseointegration/Bone formation parameters-Part III; Table S4: Osseointegration/Bone formation parameters-Part IV; Table S5: Osseointegration/Bone formation parameters-Part V; Table S6: Implant fixation properties.; Table S7: Bone remodeling/formation parameters–Part I; Table S8: Bone remodeling/formation parameters-Part II; Table S9: Bone remodeling/formation parameters-Part III; Table S10: Bone remodeling/formation parameters-Part IV.; Table S11: Bone Remodeling–Bone Formation Parameters–part V; Table S12: Bone remodeling/formation parameters-part VI; Table S13: Bone remodeling/formation biomarkers; Table S14: Bone remodeling/bone resorption markers; Table S15: Bone strength endpoints.

## References

[1] Brunkow M.E., Gardner J.C., Van Ness J., Paeper B.W., Kovacevich B.R., Proll S., Skonier J.E., Zhao L., Sabo P.J., Fu Y. (2001). Bone dysplasia sclerosteosis results from loss of the SOST gene product, a novel cystine knot-containing protein. Am. J. Hum. Genet..

[2] Balemans W., Ebeling M., Patel N., Van Hul E., Olson P., Dioszegi M., Lacza C., Wuyts W., Van Den Ende J., Willems P. (2001). Increased bone density in sclerosteosis is due to the deficiency of a novel secreted protein (SOST). Hum. Mol. Genet..

[3] Bezooijen R., Papapoulos S., Hamdy N., Dijke P., Löwik C. (2005). Control of bone formation by osteocytes? lessons from the rare skeletal disorders sclerosteosis and van Buchem disease. BoneKEy-Osteovision.

[4] van Bezooijen R.L., Roelen B.A., Visser A., van der Wee-Pals L., de Wilt E., Karperien M., Hamersma H., Papapoulos S.E., ten Dijke P., Löwik C.W.G.M. (2004). Sclerostin is an osteocyte-expressed negative regulator of bone formation, but not a classical BMP antagonist. J. Exp. Med..

[5] Poole K.E., van Bezooijen R.L., Loveridge N., Hamersma H., Papapoulos S.E., Löwik C.W., Reeve J. (2005). Sclerostin is a delayed secreted product of osteocytes that inhibits bone formation. FASEB J..

[6] Lewiecki E.M. (2014). Role of sclerostin in bone and cartilage and its potential as a therapeutic target in bone diseases. Ther. Adv. Musculoskelet. Dis..

[7] Winkler D.G., Sutherland M.S., Ojala E., Turcott E., Geoghegan J.C., Shpektor D., Skonier J.E., Yu C., Latham J.A. (2005). Sclerostin inhibition of Wnt-3a-induced C3H10T1/2 cell differentiation is indirect and mediated by bone morphogenetic proteins. J. Biol. Chem..

[8] Krishnan V., Bryant H.U., Macdougald O.A. (2006). Regulation of bone mass by Wnt signaling. J. Clin. Investig..

[9] van Bezooijen R.L., Svensson J.P., Eefting D., Visser A., van der Horst G., Karperien M., Quax P.H.A., Vrieling H., Papapoulos S.E., ten Dijke P. (2007). Wnt but not BMP signaling is involved in the inhibitory action of sclerostin on BMP-stimulated bone formation. J. Bone Miner. Res..

[10] ten Dijke P., Krause C., de Gorter D.J., Löwik C.W., van Bezooijen R.L. (2008). Osteocyte-derived sclerostin inhibits bone formation: Its role in bone morphogenetic protein and Wnt signaling. J. Bone Jt. Surg. Am..

[11] Hill T.P., Später D., Taketo M.M., Birchmeier W., Hartmann C. (2005). Canonical Wnt/beta-catenin signaling prevents osteoblasts from differentiating into chondrocytes. Dev. Cell..

[12] Hoeppner L.H., Secreto F.J., Westendorf J.J. (2009). Wnt signaling as a therapeutic target for bone diseases. Expert. Opin. Ther. Targets.

[13] Komatsu D.E., Mary M.N., Schroeder R.J., Robling A.G., Turner C.H., Warden S.J. (2010). Modulation of Wnt signaling influences fracture repair. J. Orthop. Res..

[14] Zhang R., Oyajobi B.O., Harris S.E., Chen D., Tsao C., Deng H.W., Zhao M. (2013). Wnt/β-catenin signaling activates bone morphogenetic protein 2 expression in osteoblasts. Bone.

[15] Baron R., Kneissel M. (2013). WNT signaling in bone homeostasis and disease: From human mutations to treatments. Nat. Med..

[16] Li X., Zhang Y., Kang H., Liu W., Liu P., Zhang J., Harris S.E., Wu D. (2005). Sclerostin binds to LRP5/6 and antagonizes canonical Wnt signaling. J. Biol. Chem..

[17] Sutherland M.K., Geoghegan J.C., Yu C., Turcott E., Skonier J.E., Winkler D.G., Latham J.A. (2004). Sclerostin promotes the apoptosis of human osteoblastic cells: A novel regulation of bone formation. Bone.

[18] Khosla S., Westendorf J.J., Oursler M.J. (2008). Building bone to reverse osteoporosis and repair fractures. J. Clin. Investig..

[19] Eyaid W., Al-Qattan M.M., Al Abdulkareem I., Fetaini N., Al Balwi M. (2011). A novel homozygous missense mutation (c.610G>A, p.Gly204Ser) in the WNT7A gene causes tetra-amelia in two Saudi families. Am. J. Med. Genet. A.

[20] Johnson M.L. (2012). LRP5 and bone mass regulation: Where are we now?. Bonekey Rep..

[21] Niemann S., Zhao C., Pascu F., Stahl U., Aulepp U., Niswander L., Weber J.L., Müller U. (2004). Homozygous WNT3 mutation causes tetra-amelia in a large consanguineous family. Am. J. Hum. Genet..

[22] Parr B.A., McMahon A.P. (1995). Dorsalizing signal Wnt-7a required for normal polarity of D-V and A-P axes of mouse limb. Nature.

[23] Kramer I., Loots G.G., Studer A., Keller H., Kneissel M. (2010). Parathyroid hormone (PTH)-induced bone gain is blunted in SOST overexpressing and deficient mice. J. Bone Miner. Res..

[24] Sun M., Chen Z., Wu X., Yu Y., Wang L., Lu A., Zhang G., Li F. (2021). The Roles of Sclerostin in Immune System and the Applications of Aptamers in Immune-Related Research. Front. Immunol..

[25] Shirazi S., Ravindran S., Cooper L.F. (2022). Topography-mediated immunomodulation in osseointegration; Ally or Enemy. Biomaterials.

[26] Gooi J.H., Pompolo S., Karsdal M.A., Kulkarni N.H., Kalajzic I., McAhren S.H., Han B., Onyia J.E., Ho P.W.M., Gillespie M.T. (2010). Calcitonin impairs the anabolic effect of PTH in young rats and stimulates expression of sclerostin by osteocytes. Bone.

[27] Bellido T., Ali A.A., Gubrij I., Plotkin L.I., Fu Q., O’Brien C.A., Manolagas S.C., Jilka R.L. (2005). Chronic elevation of parathyroid hormone in mice reduces expression of sclerostin by osteocytes: A novel mechanism for hormonal control of osteoblastogenesis. Endocrinology.

[28] Bellido T., Saini V., Pajevic P.D. (2013). Effects of PTH on osteocyte function. Bone.

[29] Robling A.G., Niziolek P.J., Baldridge L.A., Condon K.W., Allen M.R., Alam I., Mantila S.M., Gluhak-Heinrich J., Bellido T.M., Harris S.E. (2008). Mechanical stimulation of bone in vivo reduces osteocyte expression of Sost/sclerostin. J. Biol. Chem..

[30] Genetos D.C., Yellowley C.E., Loots G.G. (2011). Prostaglandin E2 signals through PTGER2 to regulate sclerostin expression. PLoS ONE.

[31] Walker E.C., McGregor N.E., Poulton I.J., Solano M., Pompolo S., Fernandes T.J., Constable M.J., Nicholson G.C., Zhang J.-G., Nicola N.A. (2010). Oncostatin M promotes bone formation independently of resorption when signaling through leukemia inhibitory factor receptor in mice. J. Clin. Investig..

[32] Ke H.Z., Richards W.G., Li X., Ominsky M.S. (2012). Sclerostin and Dickkopf-1 as therapeutic targets in bone diseases. Endocr. Rev..

[33] McDonald M.M., Morse A., Mikulec K., Peacock L., Yu N., Baldock P.A., Birke O., Liu M., Ke H.Z., Little D.G. (2012). Inhibition of sclerostin by systemic treatment with sclerostin antibody enhances healing of proximal tibial defects in ovariectomized rats. J. Orthop. Res..

[34] Tian X., Jee W.S., Li X., Paszty C., Ke H.Z. (2011). Sclerostin antibody increases bone mass by stimulating bone formation and inhibiting bone resorption in a hindlimb-immobilization rat model. Bone.

[35] Virdi A.S., Irish J., Sena K., Liu M., Ke H.Z., McNulty M.A., Sumner D.R. (2015). Sclerostin antibody treatment improves implant fixation in a model of severe osteoporosis. J. Bone Jt. Surg. Am..

[36] Li X., Ominsky M.S., Warmington K.S., Morony S., Gong J., Cao J., Gao Y., Shalhoub V., Tipton B., Haldankar R. (2009). Sclerostin antibody treatment increases bone formation, bone mass, and bone strength in a rat model of postmenopausal osteoporosis. J. Bone Miner. Res..

[37] Padhi D., Jang G., Stouch B., Fang L., Posvar E. (2011). Single-dose, placebo-controlled, randomized study of AMG 785, a sclerostin monoclonal antibody. J. Bone Miner. Res..

[38] Li X., Warmington K.S., Niu Q.T., Asuncion F.J., Barrero M., Grisanti M., Dwyer D., Stouch B., Thway T.M., Stolina M. (2010). Inhibition of sclerostin by monoclonal antibody increases bone formation, bone mass, and bone strength in aged male rats. J. Bone Miner. Res..

[39] Virdi A.S., Liu M., Sena K., Maletich J., McNulty M., Ke H.Z., Sumner D.R. (2012). Sclerostin antibody increases bone volume and enhances implant fixation in a rat model. J. Bone Jt. Surg. Am..

[40] Yu S.H., Hao J., Fretwurst T., Liu M., Kostenuik P., Giannobile W.V., Jin Q. (2018). Sclerostin-Neutralizing Antibody Enhances Bone Regeneration Around Oral Implants. Tissue Eng. Part. A.

[41] McClung M.R., Grauer A., Boonen S., Bolognese M.A., Brown J.P., Diez-Perez A., Langdahl B.L., Reginster J.-Y., Zanchetta J.R., Wasserman S.M. (2014). Romosozumab in postmenopausal women with low bone mineral density. N. Engl. J. Med..

[42] McColm J., Hu L., Womack T., Tang C.C., Chiang A.Y. (2014). Single- and multiple-dose randomized studies of blosozumab, a monoclonal antibody against sclerostin, in healthy postmenopausal women. J. Bone Miner. Res..

[43] Padhi D., Allison M., Kivitz A.J., Gutierrez M.J., Stouch B., Wang C., Jang G. (2014). Multiple doses of sclerostin antibody romosozumab in healthy men and postmenopausal women with low bone mass: A randomized, double-blind, placebo-controlled study. J. Clin. Pharmacol..

[44] Yao Y., Kauffmann F., Maekawa S., Sarment L.V., Sugai J.V., Schmiedeler C.A., Doherty E.J., Holdsworth G., Kostenuik P.J., Giannobile W.V. (2020). Sclerostin antibody stimulates periodontal regeneration in large alveolar bone defects. Sci. Rep..

[45] Martins B.G.S., Fernandes J.C.H., Martins A.G., Castilho R.M., Fernandes G.V.O. (2022). Surgical and Nonsurgical Treatment Protocols for Peri-implantitis: An Overview of Systematic Reviews. Int. J. Oral Maxillofac. Implant..

[46] Borges H., Correia A.R.M., Castilho R.M., Fernandes G.V.O. (2020). Zirconia Implants and Marginal Bone Loss: A Systematic Review and Meta-Analysis of Clinical Studies. Int. J. Oral Maxillofac. Implant..

[47] Fernandes G.V.O., Costa B.M.G.N., Trindade H.F., Castilho R.M., Fernandes J.C.H. (2022). Comparative analysis between extra-short implants (≤6 mm) and 6 mm-longer implants: A meta-analysis of randomized controlled trial. Aust. Dent. J..

[48] Wittneben J.G., Buser D., Salvi G.E., Bürgin W., Hicklin S., Brägger U. (2014). Complication and failure rates with implant-supported fixed dental prostheses and single crowns: A 10-year retrospective study. Clin. Implant Dent. Relat. Res..

[49] Howe M.S., Keys W., Richards D. (2019). Long-term (10-year) dental implant survival: A systematic review and sensitivity meta-analysis. J. Dent..

[50] Barfeie A., Wilson J., Rees J. (2015). Implant surface characteristics and their effect on osseointegration. Br. Dent. J..

[51] Vohra F., Al-Rifaiy M.Q., Almas K., Javed F. (2014). Efficacy of systemic bisphosphonate delivery on osseointegration of implants under osteoporotic conditions: Lessons from animal studies. Arch. Oral Biol..

[52] Mohan S., Baylink D.J. (1991). Evidence that the inhibition of TE85 human bone cell proliferation by agents which stimulate cAMP production may in part be mediated by changes in the IGF-II regulatory system. Growth Regul..

[53] Gabet Y., Müller R., Levy J., Dimarchi R., Chorev M., Bab I., Kohavi D. (2006). Parathyroid hormone 1–34 enhances titanium implant anchorage in low-density trabecular bone: A correlative micro-computed tomographic and biomechanical analysis. Bone.

[54] Le Guéhennec L., Soueidan A., Layrolle P., Amouriq Y. (2007). Surface treatments of titanium dental implants for rapid osseointegration. Dent. Mater..

[55] Moher D., Liberati A., Tetzlaff J., Altman D.G. (2009). Preferred reporting items for systematic reviews and meta-analyses: The PRISMA statement. Ann. Intern. Med..

[56] Schardt C., Adams M.B., Owens T., Keitz S., Fontelo P. (2007). Utilization of the PICO framework to improve searching PubMed for clinical questions. BMC Med. Inform. Decis. Mak..

[57] Korn P., Kramer I., Schlottig F., Tödtman N., Eckelt U., Bürki A., Ferguson S.J., Kautz A., Schnabelrauch M., Range U. (2019). Systemic sclerostin antibody treatment increases osseointegration and biomechanical competence of zoledronic-acid-coated dental implants in a rat osteoporosis model. Eur Cell Mater..

[58] Liu M., Kurimoto P., Zhang J., Niu Q.T., Stolina M., Dechow P.C., Feng J.Q., Hesterman J., Silva M.D., Ominsky M.S. (2018). Sclerostin and DKK1 Inhibition Preserves and Augments Alveolar Bone Volume and Architecture in Rats with Alveolar Bone Loss. J. Dent. Res..

[59] Wu J., Cai X.H., Qin X.X., Liu Y.X. (2018). The effects of sclerostin antibody plus parathyroid hormone (1–34) on bone formation in ovariectomized rats. Z Gerontol. Geriatr..

[60] Taut A.D., Jin Q., Chung J.H., Galindo-Moreno P., Yi E.S., Sugai J.V., Ke H.Z., Liu M., Giannobile W.V. (2013). Sclerostin antibody stimulates bone regeneration after experimental periodontitis. J. Bone Miner. Res..

[61] Virk M.S., Alaee F., Tang H., Ominsky M.S., Ke H.Z., Lieberman J.R. (2013). Systemic administration of sclerostin antibody enhances bone repair in a critical-sized femoral defect in a rat model. J. Bone Jt. Surg. Am..

[62] Liu S., Virdi A.S., Sena K., Sumner D.R. (2012). Sclerostin antibody prevents particle-induced implant loosening by stimulating bone formation and inhibiting bone resorption in a rat model. Arthritis Rheum..

[63] Ominsky M.S., Li C., Li X., Tan H.L., Lee E., Barrero M., Asuncion F.J., Dwyer D., Han C.-Y., Vlasseros F. (2011). Inhibition of sclerostin by monoclonal antibody enhances bone healing and improves bone density and strength of nonfractured bones. J. Bone Miner. Res..

[64] Agholme F., Li X., Isaksson H., Ke H.Z., Aspenberg P. (2010). Sclerostin antibody treatment enhances metaphyseal bone healing in rats. J. Bone Miner. Res..

[65] Ominsky M.S., Vlasseros F., Jolette J., Smith S.Y., Stouch B., Doellgast G., Gong J., Gao Y., Cao J., Graham K. (2010). Two doses of sclerostin antibody in cynomolgus monkeys increases bone formation, bone mineral density, and bone strength. J. Bone Miner. Res..

[66] Tian X., Setterberg R.B., Li X., Paszty C., Ke H.Z., Jee W.S. (2010). Treatment with a sclerostin antibody increases cancellous bone formation and bone mass regardless of marrow composition in adult female rats. Bone.

[67] Saag K.G., Petersen J., Brandi M.L., Karaplis A.C., Lorentzon M., Thomas T., Maddox J., Fan M., Meisner P.D., Grauer A. (2017). Romosozumab or Alendronate for Fracture Prevention in Women with Osteoporosis. N. Engl. J. Med..

[68] Fabre S., Funck-Brentano T., Cohen-Solal M. (2020). Anti-Sclerostin Antibodies in Osteoporosis and Other Bone Diseases. J. Clin. Med..

