# Supplementary material for: Antisclerostin Effect on Osseointegration and Bone Remodeling
Source: J Clin Med. 2023 Feb 6;12(4):1294. doi: 10.3390/jcm12041294 (PMC9964545; doi:10.3390/jcm12041294)
Supplement: Supplementary file 1 [file jcm-12-01294-s001.zip › Suppl. Material - Trabecular bone.docx]

#### SUPPLEMENTARY MATERIAL – TRABECULAR BONE

#### Structural Model Index

The studies performed by Liu *et al.* 2012 and Virdi *et al.* 2012 referred the Structural Model Index (SMI). The remaining studies did not mention this parameter.

Liu *et al.* 2012 reported SMI data for each group analyzed in his study, with the lowest value identified in PE suspension plus antibody vehicle group[^61^](#_ENREF_61) (Table S4). While Virdi *et al.* 2012 just related a decrease of SMI over time with the administration Scl-Ab.[^37^](#_ENREF_37)

#### Mineralizing Surface and Mineral Apposition Rate

The Relative Mineralizing Surface (MS/BS) and the Mineral Apposition Rate (MAR) was only approached by Liu *et al.* 2012 study.[^61^](#_ENREF_61) It is related that there was a higher MS/BS with the combination of intraarticular PE suspension application and with subcutaneous administration of Scl-Ab III (17.64 ± 3.5 %). And the values of MAR were reported, such as 1.11 ± 0.16 𝜇m/day, 1.56 ± 0.26 𝜇m/day and 0.77 ± 0.16 𝜇m/day in control, PE suspension plus Scl-Ab III and PE suspension plus antibody vehicle, respectively.

#### Bone Formation Rate

The Bone Formation Rate (BFR/BS) was identified by three groups.[^33^](#_ENREF_33)^,^ [^54^](#_ENREF_54)^,^ [^61^](#_ENREF_61) Virdi *et al.* 2015 reported a higher increase of BFR/BS in both OVX and Sham rats that received the Scl-Ab III treatment.[^33^](#_ENREF_33) However, was verified a decrease of BFR/BS over time.

Ominsky *et al.* 2011 study also supported the evidence of a higher increase with Scl-Ab administration.[^54^](#_ENREF_54) Nevertheless, they reported an increase over time of Ec.BFR/BS (endocortical BFR/BS) in FD and BFR/BS in FN in the Scl-Ab and control group, respectively (Table S4).

Liu *et al.* 2012 reported a higher BFR/BS with the application of PE suspension ia plus Scl-Ab III sc (102.14 ± 34.47 𝜇m^3^/𝜇m^2^/day×100).[^61^](#_ENREF_61)

#### Eroded Surface, Osteoclast Surface, and Cortical Porosity

Three studies[^33^](#_ENREF_33)^,^ [^54^](#_ENREF_54)^,^ [^61^](#_ENREF_61) reported the Eroded Surface (ES/BS) (Table S5). It was reported by Virdi et al. 2015 that, after the Scl-Ab III administration was a decrease higher than 50% of Eroded Surface in both OVX and Sham rats.[^33^](#_ENREF_33)

Liu *et al.* 2012, referred to a 10.26 ± 2.71 %, 10.83 ± 1.92 % and 17.10 ± 3.17 % of the relative Eroded Surface, in control, PE suspension plus Scl-Ab III and PE suspension plus antibody vehicle group, respectively.[^61^](#_ENREF_61) Omnisky et al. 2011 reported a lower ES/BS in the femoral neck of the primates treated by the Scl-Ab V.[^54^](#_ENREF_54)

Ominsky *et al.* 2011 was the only study that approached Osteoclast Surface (Oc.S/BS) and Cortical Porosity.[^54^](#_ENREF_54) They reported that, in FN, the Osteoclast Surface was 0.26 ± 0.09 % and 0.33 ± 0.08 % in the Scl-Ab V and control groups, respectively. And in FD, the Cortical Porosity was 0.99 ± 0.07 % and 1.13 ± 0.10 % in the Scl-Ab V and control groups, respectively.
