# Supplementary material for: Antisclerostin Effect on Osseointegration and Bone Remodeling
Source: J Clin Med. 2023 Feb 6;12(4):1294. doi: 10.3390/jcm12041294 (PMC9964545; doi:10.3390/jcm12041294)
Supplement: Supplementary file 1 [file jcm-12-01294-s001.zip › Suppl. Table 1.docx]

Table S1. Osseointegration/Bone formation parameters - Part I.

|  | Sample Size  (Initial) | | Sample Size  (Final) | | Drug/Control | Dosage & Administration Route | Implant | BIC | | BMD | | BA/TA | | | BV/TV | | |
| --- | --- | --- | --- | --- | --- | --- | --- | --- | --- | --- | --- | --- | --- | --- | --- | --- | --- |
| Korn *et al.*  (2019) [61] | 128 | | 124 | | sclerostin antibody | 100mg/kg iv once week | reference-coated implant | **HMM** | 2 weeks: 33.2 ± 18.5 %  4 weeks: 24.1 ± 9.7 % | - | | **HMM** | 4 weeks:  10.9 ± 4.4 % | | - | | |
|  |  |  |  |  |  |  |  | **𝜇CT** | - |  | |  |  |  |  |  |  |
|  |  |  |  |  |  |  | ZOL-coated implant | **HMM** | 2 weeks: comparable to reference implant  4 weeks: 57.4 ± 15.0 % | **𝜇CT** | 4 weeks: ≈ 2 times increase, comparing to reference implant | **HMM** | 4 weeks:  32.3 ± 11.5 % | | **𝜇CT** | | 4 weeks:  31.0 ± 7.6 % |
|  |  |  |  |  |  |  |  | **𝜇CT** | 4 weeks: 60.0 ± 2.5 % |  |  |  |  |  |  |  |  |
|  |  |  |  |  | non antibody applied | - | reference-coated implant | **𝜇CT** | 4 weeks: nonsignificant decrease | - | | **HMM** | | 4 weeks:  4.5 ± 4.2 % | | - | |
|  |  |  |  |  |  |  | ZOL-coated implant | **𝜇CT** | 4 weeks: 47.8 ± 10.4 % | - | | **HMM** | | 4 weeks:  23.8 ± 8.6 % | | - | |
| Yu *et al.*  (2018) [40] | 60 | | 60 | | Scl-Ab | 25mg/kg sc | cp-Ti, solid cylinder with titanium plasma-sprayed surface implant | 10 & 14 days: no differences compared to control group  28 weeks: significantly greater than control group | | No differences between both groups | | - | | | 14 days: ≈ 2x greater  28 days: 2.5x greater | | |
|  |  |  |  |  | PBS | - |  | - | |  |  | - | | | - | | |
| Virdi *et al.*  (2015) [35] | 144 | 72 OVX | 142 | 71 OVX | Scl-Ab III | 25 mg/kg sc twice week | cp-Ti with dual acid-etched surface implant | increase over time, lower than sham group | | - | | - | | | - | | |
|  |  |  |  |  | vehicle | - |  | - | | - | | - | | | - | | |
|  |  | 72 Sham |  | 71 Sham | Scl-Ab III | 25 mg/kg sc twice week |  | increase over time, higher than OVX group | | - | | - | | | most significant increase than OVX group | | |
|  |  |  |  |  | vehicle | - |  | - | | - | | - | | | - | | |
| Liu *et al.*  (2012) [66] | 36 | | 36 | | PE suspension + Scl-Ab III | 50𝜇L ia once week + 25 mg/kg sc twice week | titanium rods with dual acid-etched surface | - | | - | | - | | | 31.2 ± 7.7 % | | |
|  |  |  |  |  | PE suspension + antibody vehicle | 50𝜇L ia once week + vehicle  sc twice week |  | - | | - | | - | | | 7.6 ± 2.5 % | | |
|  |  |  |  |  | particle vehicle + antibody vehicle | - |  | - | | - | | - | | | 17.5 ± 5.8 % | | |
| Virdi *et al.*  (2012) [39] | 90 | | 88 | | Scl-Ab | 25mg/kg sc | cp-Ti with dual acid-etched surface implant | higher at later times | | - | | - | | | 4 weeks: 2x control grp  8 weeks: more than 2x | | |
|  |  |  |  |  | saline solution | - |  | - | | - | | - | | | - | | |
| Ominsky *et al.* (2011) [59] | 43 | | 29 | | Scl-Ab V | 30mg/kg sc every 2 weeks | stainless steel K-wire | - | | TH: 14.5 ± 1.8 %  FN: 17.4 ± 1.6 %  DR: 5.6 ± 0.9 %  LS: 16.6 ± 1.2 % | | - | | | FN: 33.6 ± 2.1 % | | |
|  |  |  |  |  | vehicle | - |  | - | | TH: 9.3 ± 1.5 %  FN: 7.6 ± 2.1 %  DR: 3.3 ± 0.6 %  LS: 4.4 ± 0.5 % | | - | | | FN: 27.5 ± 2.3 % | | |
| Agholme *et al.* (2010) [63] | 68 | | 64 | | Scl-Ab III | 25mg/kg sc twice weeks | stainless steel screws (mechanical tests); PMMA screws (𝜇CT) | - | | AS: 1.17 ± 0.04 g/cm^3^  MS: 1.14 ± 0.04 g/cm^3^  CS: 1.20 ± 0.055 g/cm^3^  IT: 1.04 ± 0.01 g/cm^3^  CT: 1.05 ± 0.01 g/cm^3^ | | - | | | AS: 37 ± 7.7 %  MS: 31 ± 6.6 %  CS: 65 ± 11 %  IT: 23 ± 4.4 %  CT: 26 ± 4.7% | | |
|  |  |  |  |  | saline solution | - |  | - | | AS: 1.12 ± 0.05 g/cm^3^  MS: 1.10 ± 0.02 g/cm^3^  CS: 1.14 ± 0.065 g/cm^3^  IT: 0.96 ± 0.02 g/cm^3^  CT: 0.98 ± 0.03 g/cm^3^ | | - | | | AS: 28 ± 6.9 %  MS: 25 ± 6.1 %  CS: 51 ± 12 %  IT: 19 ± 4.0 %  CT: 23 ± 3.8% | | |

BIC – Bone-to-Implant Contact; BMD – Bone Mineral Density; BA/TA – Bone Area per Total Area; BV/TV – Bone Volume Fraction; HMM – Histomorphometry; 𝜇CT – Micro Computed Tomography; TH – Total Hip; FN – Femoral Neck; DR – Third Distal Radius; LS – Lumbar Spine; AS – Around Entire Screw; MS – Marrow Surrounding; CS – Cortical Surrounding; IT – Implanted Tibia; CT – Contralateral Tibia.
