# Supplementary material for: Antisclerostin Effect on Osseointegration and Bone Remodeling
Source: J Clin Med. 2023 Feb 6;12(4):1294. doi: 10.3390/jcm12041294 (PMC9964545; doi:10.3390/jcm12041294)
Supplement: Supplementary file 1 [file jcm-12-01294-s001.zip › Suppl. Table 10.docx]

Table S10. Bone remodeling/formation parameters - Part IV.

|  | Sample Size  (Initial) | | Sample Size  (Final) | | Drug/Control | Dosage &  Administration Route | Tb.Th | | Tb.N | | Tb.Sp | | Ct.Th | |
| --- | --- | --- | --- | --- | --- | --- | --- | --- | --- | --- | --- | --- | --- | --- |
| Liu *et al.*  (2018) [57] | 50 | 40 OVX | 50 | 40 OVX | Scl-Ab VI | 18.2mg/kg sc twice week | higher increase than both control (Sham & OVX) | | - | | - | | - | |
|  |  |  |  |  | Scl-Ab VI + DAB | 18.1mg/kg sc + 18.1mg/kg sc twice week |  |  | - | | - | | - | |
|  |  |  |  |  | saline vehicle | - | - | | - | | - | | - | |
|  |  | 10 Sham |  | 10 Sham | saline vehicle | - | - | | - | | - | | - | |
|  | 45 | | 45 | | Scl-Ab VI | 25mg/kg sc twice week | - | | - | | - | | - | |
|  |  |  |  |  | Scl-Ab VI + DAB | 25mg/kg sc + 25mg/kg sc twice week | - | | - | | - | | - | |
|  |  |  |  |  | saline vehicle | - | - | | - | | - | | - | |
| Wu *et al.*  (2018) [60] | 40 OVX | | 40 OVX | | Scl-Ab | 25mg/kg sc.twice week | 1.27 higher increase vs control | | 1.59 higher increase vs control | | 2.1 higher decrease vs control | | - | |
|  |  |  |  |  | PTH 1-34 | 60𝜇g/kg sc thrice week | 1.29 higher increase vs control | | 1.60 higher increase vs control | | 2.2 higher decrease vs control | | - | |
|  |  |  |  |  | Scl-Ab +  PTH 1-34 | 25mg/kg sc twice week + 60𝜇g/kg sc thrice week | 1.66 higher increase vs control | | 1.85 higher increase vs control | | 3.31 higher decrease vs control | | - | |
|  |  |  |  |  | vehicle | - | - | | - | | - | | - | |
| Taut *et al.*  (2013) [65] | 69 | | 69 | | EP: Scl-Ab III | 25 mg/kg sc twice week | - | | - | | - | | - | |
|  |  |  |  |  |  | 15 𝜇L of 35.6mg/mL solution locally twice week | - | | - | | - | | - | |
|  |  |  |  |  | EP: vehicle | - | - | | - | | - | | - | |
|  |  |  |  |  | healthy: PBS | - | - | | - | | - | | - | |
| Virk *et al.*  (2013) [58] | 72 | | 72 | | Scl-Ab III | 25mg/kg sc twice week | - | | - | | - | | - | |
|  |  |  |  |  | PBS | - | - | | - | | - | | - | |
|  | 30 | | 30 | | Scl-Ab III | 25mg/kg | - | | - | | - | | - | |
|  |  |  |  |  | PBS | - | - | | - | | - | | - | |
| McDonald *et al.* (2012) [33] | 132 | 66 Sham | 127 | | Scl-Ab III | 25mg/kg sc twice week | 1 week: 53.5 ± 9.39 𝜇m^2^  2 weeks: 96.1 ± 10.5 𝜇m^2^  3 weeks: 169.8 ± 40.5 𝜇m^2^ | | 1 week: 1.52 ± 0.91 N/mm  2 weeks: 3.30 ± 0.57 N/mm  3 weeks: 2.16 ± 0.46 N/mm | | - | | - | |
|  |  |  |  |  | saline solution | - | 1 week: 58.3 ± 9.15 𝜇m^2^  2 weeks: 84.6 ± 9.1 𝜇m^2^  3 weeks: 148.9 ± 64.3 𝜇m^2^ | | 1 week: 1.37 ± 0.73 N/mm  2 weeks: 3.30 ± 0.47 N/m  3 weeks: 2.02 ± 0.61 N/mm | | - | | - | |
|  |  | 66 OVX |  |  | Scl-Ab III | 25mg/kg sc twice week | 1 week: 63.0 ± 11.3 𝜇m^2^  2 weeks: 105.3 ± 18.7 𝜇m^2^  3 weeks: 199.1 ± 95.9 𝜇m^2^ | | 1 week: 2.87 ± 0.86 N/mm  2 weeks: 2.40 ± 0.53 N/mm  3 weeks: 1.09 ± 0.48 N/mm | | - | | - | |
|  |  |  |  |  | saline solution | - | 1 week: 69.8 ± 27.2 𝜇m^2^  2 weeks: 89.5 ± 12.1 𝜇m^2^  3 weeks: 168.6 ± 77.2 𝜇m^2^ | | 1 week: 2.84 ± 1.15 N/mm  2 weeks: 1.72 ± 0.50 N/mm  3 weeks: 0.88 ± 0.23 N/mm | | - | | - | |
| Ominsky *et al.*  (2011) [59] | 35 | | 32 | | Scl-Ab III | 25mg/kg sc twice week | **Intact Femur:**  DF: 97.4 ± 2.7 𝜇m | | - | | - | | **Intact Femur:**  FD: 922 ± 18 𝜇m | |
|  |  |  |  |  | vehicle | - | **Intact Femur:**  DF: 56.5 ± 1.4 𝜇m | | - | | - | | **Intact Femur:**  FD: 838 ± 19 𝜇m | |
| Tian *et al*.  (2011) [34] | 67 | | 67 | | **Baseline** | | PTM: 45.3 ± 6.4 𝜇m | | PTM: 3.1 ± 0.7 #/mm | | PTM: 297.8 ± 99.0 𝜇m | | PTM: 645 ± 33 𝜇m | |
|  |  |  |  |  | Scl-Ab III | 5mg/kg sc twice week | NL.PTM: 75.5 ± 9.9 𝜇m  UL.PTM: 57.9 ± 5.7 𝜇m | | NL.PTM: 3.2 ± 1.0 #/mm  UL.PTM: 3.4 ± 0.4 #/mm | | NL.PTM: 310.9 ± 271.9 𝜇m  UL.PTM: 245.0 ± 42.2 𝜇m | | NL.PTM: 677 ± 18 𝜇m  UL.PTM: 686 ± 37 𝜇m | |
|  |  |  |  |  |  | 25mg/kg sc twice week | NL.PTM: 93.6 ± 10.7 𝜇m  UL.PTM: 71.3 ± 7.1 𝜇m | | NL.PTM: 3.7 ± 0.3 #/mm  UL.PTM: 2.9 ± 0.7 #/mm | | NL.PTM: 180.3 ± 33.3 𝜇m  UL.PTM: 300.3 ± 156.1 𝜇m | | NL.PTM: 723 ± 43 𝜇m  UL.PTM: 723 ± 44 𝜇m | |
|  |  |  |  |  | saline solution | - | NL.PTM: 44.5 ± 2.8 𝜇m  UL.PTM: 41.9 ± 3.3 𝜇m | | NL.PTM: 3.1 ± 0.5 #/mm  UL.PTM: 3.2 ± 0.4 #/mm | | NL.PTM: 285.2 ± 65.6 𝜇m  UL.PTM: 271.6 ± 36.7 𝜇m | | NL.PTM: 658 ± 36 𝜇m  UL.PTM: 651 ± 52 𝜇m | |
| Li *et al.*  (2010) [38] | 28 | | 26 | | Scl-Ab III | 25mg/kg sc twice week | **HMM** | PT: 144.7 ± 12.4 𝜇m | **HMM** | PT: 1.31 ± 0.11 *n*/mm | **HMM** | PT: 661 ± 66 𝜇m | **HMM** | PT: 1.14 ± 0.02 mm |
|  |  |  |  |  |  |  | **𝜇CT** | LV: 138.0 ± 4.6 𝜇m  DF: 124.5 ± 7.0 𝜇m | **𝜇CT** | LV: 3.45 ± 0.15mm^-1^  DF: 2.15 ± 0.20mm^-1^ | **𝜇CT** | LV: 267 ± 31 𝜇m  DF: 512.3 ± 49.2 𝜇m | **𝜇CT** | LV: 325 ± 9 𝜇m  DF: 0.948 ± 0.021 mm |
|  |  |  |  |  |  | 5mg/kg sc twice week | **HMM** | PT: 137.8 ± 7.0 𝜇m | **HMM** | PT: 1.22 ± 0.22 *n*/mm | **HMM** | PT: 829 ± 145 𝜇m | **HMM** | PT: 1.03 ± 0.05 mm |
|  |  |  |  |  |  |  | **𝜇CT** | LV: 108.8 ± 4.6 𝜇m  DF: 109.7 ± 6.2 𝜇m | **𝜇CT** | LV: 3.37 ± 0.17 mm^-1^  DF: 2.12 ± 0.24 mm^-1^ | **𝜇CT** | LV: 307 ± 31 𝜇m  DF: 518.7 ± 42.0 𝜇m | **𝜇CT** | LV: 291 ± 9 𝜇m  DF: 0.980 ± 0.040 mm |
|  |  |  |  |  | vehicle | - | **HMM** | PT: 74.3 ± 2.8 𝜇m | **HMM** | PT: 0.96 ± 0.10 *n*/mm | **HMM** | PT: 1086 ± 133 𝜇m | **HMM** | PT: 1.02 ± 0.02 mm |
|  |  |  |  |  |  |  | **𝜇CT** | LV: 60.1 ± 1.9 𝜇m  DF: 60.6 ± 1.4 𝜇m | **𝜇CT** | LV: 3.34 ± 0.17 mm^-1^  DF: 1.27 ± 0.17 mm^-1^ | **𝜇CT** | LV: 324 ± 23 𝜇m  DF: 741.0 ± 52.9 𝜇m | **𝜇CT** | LV: 231 ± 6 𝜇m  DF: 0.803 ± 0.037 mm |
| Ominsky *et al.*  (2010) [64] | 12 | | 12 | | Scl-Ab IV | 3mg/kg sc once month | - | | - | | - | | **pQCT** | DRD: 7.3 ± 7.1 %  PTD: 13.4 ± 14.2 % |
|  |  |  |  |  |  | 10mg/kg sc once month | - | | - | | - | | **pQCT** | DRD: 1.6 ± 1.1 %  PTD: 10.8 ± 4.3 % |
|  |  |  |  |  |  | 30mg/kg sc once month | - | | - | | - | | **pQCT** | DRD: 4.3 ± 2.4 %  PTD: 10.2 ± 2.6 % |
|  |  |  |  |  | vehicle | - | **-** | | - | | - | | **pQCT** | DRD: 2.1 ± 0.9 %  PTD: 0.6 ± 3.4 % |
| Tian *et al.*  (2010) [62] | 32 | | 32 | | **Baseline** | | CVB: 50.8 ± 6.0 𝜇m  LVB: 65.1 ± 11.1 𝜇m | | CVB: 5.1 ± 0.7 #/mm  LVB: 3.9 ± 0.3 #/mm | | CVB: 149.8 ± 24.7 𝜇m  LVB: 195.4 ± 22.5 𝜇m | | - | |
|  |  |  |  |  | Scl-Ab III | 5mg/kg sc twice week | CVB: 54.5 ± 10.5 𝜇m  LVB: 91.6 ± 6.8 𝜇m | | CVB: 5.5 ± 0.7 #/mm  LVB: 3.5 ± 0.2 #/mm | | CVB: 130.5 ± 16.7 𝜇m  LVB: 197.1 ± 20.5 𝜇m | | - | |
|  |  |  |  |  |  | 25mg/kg sc twice week | CVB: 65.3 ± 7.0 𝜇m  LVB: 119.4 ± 17.7 𝜇m | | CVB: 5.7 ± 0.6 #/mm  LVB: 3.8 ± 0.4 #/mm | | CVB: 111.2 ± 21.9 𝜇m  LVB: 144.5 ± 18.7 𝜇m | | - | |
|  |  |  |  |  | saline solution | - | CVB: 45.7 ± 6.8 𝜇m  LVB: 62.2 ± 7.2 𝜇m | | CVB: 5.2 ± 0.4 #/mm  LVB: 3.9 ± 0.6 #/mm | | CVB: 147.4 ± 15.3 𝜇m  LVB: 196.2 ± 35.7 𝜇m | | - | |
| Saag *et al.*  (2017) [67] | 4093 | | 3150 | | Romosozumab → Alendronate | 210mg sc once month → 70mg po once week | - | | - | | - | | - | |
|  |  |  |  |  | Alendronate → Alendronate | 70mg po once week → 70mg po once week | - | | - | | - | | - | |
| McClung *et al.*  (2014) [41] | 419 | | 383 | | Romosozumab | 140mg sc every 3 months | - | | - | | - | | - | |
|  |  |  |  |  |  | 210mg sc every 3 months | - | | - | | - | | - | |
|  |  |  |  |  |  | 70mg sc once month | - | | - | | - | | - | |
|  |  |  |  |  |  | 140mg sc once month | - | | - | | - | | - | |
|  |  |  |  |  |  | 210mg sc once month | - | | - | | - | | - | |
|  |  |  |  |  | alendronate | 70 mg po once week | - | | - | | - | | - | |
|  |  |  |  |  | teriparatide | 20𝜇g sc once day | - | | - | | - | | - | |
|  |  |  |  |  | placebo | - | - | | - | | - | | - | |
| Padhi *et al.*  (2014) [43] | 48 | 32 women | 46 | 31 women | Romosozumab | 1mg/kg sc every 2 weeks | - | | - | | - | | - | |
|  |  |  |  |  |  | 2mg/kg sc every 4 weeks | - | | - | | - | | - | |
|  |  |  |  |  |  | 2mg/kg sc every 2 weeks | - | | - | | - | | - | |
|  |  |  |  |  |  | 3mg/kg sc every 4 weeks | - | | - | | - | | - | |
|  |  |  |  |  | placebo | - | - | | - | | - | | - | |
|  |  | 16 men |  | 15 men |  |  |  |  |  |  |  |  |  |  |
|  |  |  |  |  | Romosozumab | 1mg/kg sc every 2 weeks | - | | - | | - | | - | |
|  |  |  |  |  |  | 3mg/kg sc every 4 weeks | - | | - | | - | | - | |

Tb.Th – Trabecular Thickness; Tb.N – Trabecular Number; Tb.Sp – Trabecular Separation; Ct.Th – Cortical Thickness; DF – Distal Femur; FD – Femoral Diaphysis; PTM – Proximal Tibial Metaphysis; NL – Normal-loaded; UL – Under-loaded; HMM – Histomorphometry; 𝜇CT – Micro computed tomography; PT – Proximal Tibia; LV – 5^th^ Lumbar Vertebra; pQCT – Peripheral Quantitative Computed Tomography; DRD – Distal Radius Diaphysis; PTD – Proximal Tibial Diaphysis; CVB – Caudal Vertebral Body; LVB – Lumbar Vertebral Body.
