# Supplementary material for: Antisclerostin Effect on Osseointegration and Bone Remodeling
Source: J Clin Med. 2023 Feb 6;12(4):1294. doi: 10.3390/jcm12041294 (PMC9964545; doi:10.3390/jcm12041294)
Supplement: Supplementary file 1 [file jcm-12-01294-s001.zip › Suppl. Table 11.docx]

Table S11. Bone Remodeling – Bone Formation Parameters – part V.

|  | Sample Size  (Initial) | | | Sample Size  (Final) | | | Drug/Control | Dosage &  Administration Route | SMI | MS/BS | | MAR | | BFR/BS | |
| --- | --- | --- | --- | --- | --- | --- | --- | --- | --- | --- | --- | --- | --- | --- | --- |
| Liu *et al.*  (2018) [57] | 50 | | 40 OVX | 50 | | 40 OVX | Scl-Ab VI | 18.2mg/kg sc twice week | - | - | | - | | sig. higher in basal & alveolar bone vs control | |
|  |  |  |  |  |  |  | Scl-Ab VI + DAB | 18.1mg/kg sc + 18.1mg/kg sc twice week | - | - | | - | | sig. higher in basal & alveolar bone vs control; higher than Scl-Ab group, in basal bone | |
|  |  |  |  |  |  |  | saline vehicle | - | - | - | | - | | - | |
|  |  |  | 10 Sham |  |  | 10 Sham | saline vehicle | - | - | - | | - | | - | |
|  | 45 | | | 45 | | | Scl-Ab VI | 25mg/kg sc twice week | - | - | | - | | - | |
|  |  |  |  |  |  |  | Scl-Ab VI + DAB | 25mg/kg sc + 25mg/kg sc twice week | - | - | | - | | - | |
|  |  |  |  |  |  |  | saline vehicle | - | - | - | | - | | - | |
| Wu *et al.*  (2018) [60] | 40 OVX | | | 40 OVX | | | Scl-Ab | 25mg/kg sc twice week | - | - | | - | | - | |
|  |  |  |  |  |  |  | PTH 1-34 | 60𝜇g/kg sc thrice week | - | - | | - | | - | |
|  |  |  |  |  |  |  | Scl-Ab +  PTH 1-34 | 25mg/kg sc twice week + 60𝜇g/kg sc thrice week | - | - | | - | | - | |
|  |  |  |  |  |  |  | vehicle | - | - | - | | - | | - | |
| Taut *et al.*  (2013) [65] | 69 | | | 69 | | | EP: Scl-Ab III | 25 mg/kg sc twice week | - | - | | - | | - | |
|  |  |  |  |  |  |  |  | 15 𝜇L of 35.6mg/mL solution locally twice week | - | - | | - | | - | |
|  |  |  |  |  |  |  | EP: vehicle | - | - | - | | - | | - | |
|  |  |  |  |  |  |  | healthy: PBS | - | - | - | | - | | - | |
| Virk *et al.*  (2013) [58] | 72 | | | 72 | | | Scl-Ab III | 25mg/kg sc twice week | - | - | | - | | - | |
|  |  |  |  |  |  |  | PBS | - | - | - | | - | | - | |
|  | 30 | | | 30 | | | Scl-Ab III | 25mg/kg | - | - | | - | | - | |
|  |  |  |  |  |  |  | PBS | - | - | - | | - | | - | |
| McDonald *et al.* (2012) [33] | 132 | | 66 Sham | 127 | | | Scl-Ab III | 25mg/kg sc twice week | - | - | | - | | - | |
|  |  |  |  |  |  |  | saline solution | - | - | - | | - | | - | |
|  |  |  | 66 OVX |  |  |  | Scl-Ab III | 25mg/kg sc twice week | - | - | | - | | - | |
|  |  |  |  |  |  |  | saline solution | - | - | - | | - | | - | |
| Ominsky *et al.*  (2011) [59] | 35 | | | 32 | | | Scl-Ab III | 25mg/kg sc twice week | - | - | | - | | - | |
|  |  |  |  |  |  |  | vehicle | - | - | - | | - | | - | |
| Tian *et al*.  (2011) [34] | 67 | | | 67 | | | **Baseline** | | - | PTM: 24.6 ± 7.3 %  Ps.TS: 26.1 ± 7.8 %  Ec.TS: 17.3 ± 7.0 % | | PTM: 0,7 ± 0,1 𝜇m/day  Ps.TS: 0.5 ± 0.2 𝜇m/day  Ec.TS: 0.5 ± 0.1 𝜇m/day | | PTM: 17.9 ± 6.1 𝜇m^3^/𝜇m^2^/day×100  Ps.TS: 12.7 ± 6.5 𝜇m^3^/𝜇m^2^/day×100  Ec.TS: 9.5 ± 5.4 𝜇m^3^/𝜇m^2^/day×100 | |
|  |  |  |  |  |  |  | Scl-Ab III | 5mg/kg sc twice week | - | **NL** | PTM: 55.1 ± 3.8 %  Ps.TS: 46.3 ± 15.8 %  Ec.TS: 65.1 ± 13.9 % | **NL** | PTM: 1.0 ± 0.1 𝜇m/day  Ps.TS: 0.7 ± 0.1 𝜇m/day  Ec.TS: 1.5 ± 0.2 𝜇m/day | **NL** | PTM: 55.4 ± 8.7 𝜇m^3^/𝜇m^2^/day×100  Ps.TS: 34.9 ±17.7 𝜇m^3^/𝜇m^2^/day×100  Ec.TS: 99.5 ± 25.3 𝜇m^3^/𝜇m^2^/day×100 |
|  |  |  |  |  |  |  |  |  |  | **UL** | PTM: 44.4 ± 5.0 %  Ps.TS: 48.2 ± 13.7 %  Ec.TS: 51.0 ± 10.0 % | **UL** | PTM: 0.8 ± 0.1 𝜇m/day  Ps.TS: 1.0 ± 0.1 𝜇m/day  Ec.TS: 1.3 ± 0.3 𝜇m/day | **UL** | PTM: 37.6 ± 6.9 𝜇m^3^/𝜇m^2^/day×100  Ps.TS: 48.6 ± 18.4 𝜇m^3^/𝜇m^2^/day×100  Ec.TS: 67.8 ± 23.2 𝜇m^3^/𝜇m^2^/day×100 |
|  |  |  |  |  |  |  |  | 25mg/kg sc twice week | - | **NL** | PTM: 69.2 ± 2.6 %  Ps.TS: 85.9 ± 11.0 %  Ec.TS: 84.9 ± 12.5 % | **NL** | PTM: 1.1 ± 0.1 𝜇m/day  Ps.TS: 1.1 ± 0.2 𝜇m/day  Ec.TS: 1.7 ± 0.1 𝜇m/day | **NL** | PTM: 75.7 ± 9.6 𝜇m^3^/𝜇m^2^/day×100  Ps.TS: 95.9 ± 27.9 𝜇m^3^/𝜇m^2^/day×100  Ec.TS: 148.9 ± 27.0 𝜇m^3^/𝜇m^2^/day×100 |
|  |  |  |  |  |  |  |  |  |  | **UL** | PTM: 56.8 ± 7.2 %  Ps.TS: 72.7 ± 10.1 %  Ec.TS: 84.5 ± 9.7 % | **UL** | PTM: 1.0 ± 0.2 𝜇m/day  Ps.TS: 1.7 ± 0.2 𝜇m/day  Ec.TS: 1.7 ± 0.2 𝜇m/day | **UL** | PTM: 55.7 ± 15.1 𝜇m^3^/𝜇m^2^/day×100  Ps.TS: 120.0 ± 26.1 𝜇m^3^/𝜇m^2^/day×100  Ec.TS: 141 ± 26 𝜇m^3^/𝜇m^2^/day×100 |
|  |  |  |  |  |  |  | saline solution | - | - | **NL** | PTM: 27.6 ± 4.5 %  Ps.TS: 30.6 ± 12.6 %  Ec.TS: 25.1 ± 6.9 % | **NL** | PTM: 0,7 ± 0.1 𝜇m/day  Ps.TS: 0.5 ± 0.2 𝜇m/day  Ec.TS: 0.6 ± 0.2 𝜇m/day | **NL** | PTM: 20.1 ± 3.1 𝜇m^3^/𝜇m^2^/day×100  Ps.TS: 17.1 ± 11.2 𝜇m^3^/𝜇m^2^/day×100  Ec.TS: 16.7 ± 8.7 𝜇m^3^/𝜇m^2^/day×100 |
|  |  |  |  |  |  |  |  |  |  | **UL** | PTM: 25.7 ± 2.3 %  Ps.TS: 24.0 ± 8.8 %  Ec.TS: 19.3 ± 2.3 % | **UL** | PTM: 0,6 ± 0.1 𝜇m/day  Ps.TS: 0.9 ± 0.3 𝜇m/day  Ec.TS: 0.5 ± 0.2 𝜇m/day | **UL** | PTM: 14.1 ± 3.6 𝜇m^3^/𝜇m^2^/day×100  Ps.TS: 22.0 ± 12.7 𝜇m^3^/𝜇m^2^/day×100  Ec.TS: 10.2 ± 2.7 𝜇m^3^/𝜇m^2^/day×100 |
| Li *et al.*  (2010) [38] | 28 | | | 26 | | | Scl-Ab III | 25mg/kg sc twice week | LV: -0.99 ± 0.32 | PT: 74.7 ± 2.5 %  Ps.TS: 99.8 ± 1.0 %  Ec.TS: 84.5 ± 4.3 % | | PT: 1.59 ± 0.08 𝜇m/day  Ps.TS: 1.92 ± 0.11 𝜇m/day  Ec.TS: 1.66 ± 0.14 𝜇m/day | | PT: 1.20 ± 0.08 𝜇m^3^/𝜇m^2^/day  Ps.TS: 1.92 ± 0.12 𝜇m^3^/𝜇m^2^/day  Ec.TS: 1.43 ± 0.17 𝜇m^3^/𝜇m^2^/day | |
|  |  |  |  |  |  |  |  | 5mg/kg sc twice week | LV: -0.49 ± 0.24 | PT: 68.7 ± 2.7 %  Ps.TS: 98.1 ± 2.1 %  Ec.TS: 69.0 ± 6.9 % | | PT: 1.57 ± 0.10 𝜇m/day  Ps.TS: 2.13 ± 0.11 𝜇m/day  Ec.TS: 1.24 ± 0.05 𝜇m/day | | PT: 1.09 ± 0.10𝜇m^3^/𝜇m^2^/day  Ps.TS: 2.10 ± 0.13 𝜇m^3^/𝜇m^2^/day  Ec.TS: 0.84 ± 0.07 𝜇m^3^/𝜇m^2^/day | |
|  |  |  |  |  |  |  | vehicle | - | LV: 0.40 ± 0.14 | PT: 26.0 ± 2.2 %  Ps.TS: 20.7 ± 3.6 %  Ec.TS: 36.7 ± 8.2 % | | PT: 0.98 ± 0.02 𝜇m/day  Ps.TS: 0.79 ± 0.18 𝜇m/day  Ec.TS: 0.71 ± 0.17 𝜇m/day | | PT: 0.25 ± 0.02 𝜇m^3^/𝜇m^2^/day  Ps.TS: 0.20 ± 0.06 𝜇m^3^/𝜇m^2^/day  Ec.TS: 0.37 ± 0.11 𝜇m^3^/𝜇m^2^/day | |
| Ominsky *et al.*  (2010) [64] | 12 | | | 12 | | | Scl-Ab IV | 3mg/kg sc once month | - | - | | - | | - | |
|  |  |  |  |  |  |  |  | 10mg/kg sc once month | - | - | | - | | - | |
|  |  |  |  |  |  |  |  | 30mg/kg sc once month | - | - | | - | | sig. increase in Ec.BFR/BS & non sig. increase in Ps.BFR/BS | |
|  |  |  |  |  |  |  | vehicle | - | **-** | - | | - | | - | |
| Tian *et al.*  (2010) [62] | 32 | | | 32 | | | **Baseline** | | - | CVB: 5.3 ± 4.8 %  LVB: 25.9 ± 8.6 % | | CVB: 0.4 ± 0.1 𝜇m/day  LVB: 0.7 ± 0.1 𝜇m/day | | CVB: 1.9 ± 1.4 𝜇m^3^/𝜇m^2^/day×100  LVB: 16.9 ± 6.3 𝜇m^3^/𝜇m^2^/day×100 | |
|  |  |  |  |  |  |  | Scl-Ab III | 5mg/kg sc twice week | - | CVB: 22.2 ± 16.3 %  LVB: 59.6 ± 5.7 % | | CVB: 0.6 ± 0.1 𝜇m/day  LVB: 0.9 ± 0.0 𝜇m/day | | CVB: 12.6 ± 9.6 𝜇m^3^/𝜇m^2^/day×100  LVB: 54.2 ± 4.0 𝜇m^3^/𝜇m^2^/day×100 | |
|  |  |  |  |  |  |  |  | 25mg/kg sc twice week | - | CVB: 47.5 ± 13.2 %  LVB: 78.7 ± 4.1 % | | CVB: 0.6 ± 0.0 𝜇m/day  LVB: 1.0 ± 0.1 𝜇m/day | | CVB: 30.2 ± 8.2 𝜇m^3^/𝜇m^2^/day×100  LVB: 79.0 ± 6.6 𝜇m^3^/𝜇m^2^/day×100 | |
|  |  |  |  |  |  |  | saline solution | - | - | CVB: 7.0 ± 3.3 %  LVB: 23.7 ± 6.3 % | | CVB: 0.4 ± 0.1 𝜇m/day  LVB: 0.6 ± 0.1 𝜇m/day | | CVB: 2.9 ± 1.4 𝜇m^3^/𝜇m^2^/day×100  LVB: 14.7 ± 5.1 𝜇m^3^/𝜇m^2^/day×100 | |
| Saag *et al.*  (2017) [67] | 4093 | | | 3150 | | | Romosozumab → Alendronate | 210mg sc once month → 70mg po once week | - | - | | - | | - | |
|  |  |  |  |  |  |  | Alendronate → Alendronate | 70mg po once week → 70mg po once week | - | - | | - | | - | |
| McClung *et al.*  (2014) [41] | 419 | | | 383 | | | Romosozumab | 140mg sc every 3 months | - | - | | - | | - | |
|  |  |  |  |  |  |  |  | 210mg sc every 3 months | - | - | | - | | - | |
|  |  |  |  |  |  |  |  | 70mg sc once month | - | - | | - | | - | |
|  |  |  |  |  |  |  |  | 140mg sc once month | - | - | | - | | - | |
|  |  |  |  |  |  |  |  | 210mg sc once month | - | - | | - | | - | |
|  |  |  |  |  |  |  | Alendronate | 70 mg po once week | - | - | | - | | - | |
|  |  |  |  |  |  |  | Teriparatide | 20𝜇g sc once day | - | - | | - | | - | |
|  |  |  |  |  |  |  | placebo | - | - | - | | - | | - | |
| Padhi *et al.*  (2014) [43] | 48 | 32 women | | 46 | 31 women | | Romosozumab | 1mg/kg sc every 2 weeks | - | - | | - | | - | |
|  |  |  |  |  |  |  |  | 2mg/kg sc every 4 weeks | - | - | | - | | - | |
|  |  |  |  |  |  |  |  | 2mg/kg sc every 2 weeks | - | - | | - | | - | |
|  |  |  |  |  |  |  |  | 3mg/kg sc every 4 weeks | - | - | | - | | - | |
|  |  |  |  |  |  |  | placebo | - | - | - | | - | | - | |
|  |  | 16 men | |  | 15 men | |  |  |  |  |  |  |  |  |  |
|  |  |  |  |  |  |  | Romosozumab | 1mg/kg sc every 2 weeks | - | - | | - | | - | |
|  |  |  |  |  |  |  |  | 3mg/kg sc every 4 weeks | - | - | | - | | - | |

SMI – Structural Model Index; MS/BS – Mineralizing Surface; MAR – Mineral Apposition Rate; BFR/BS – Bone Formation Rate; PTM – Proximal Tibia Metaphysis; Ps – Periosteal; Ec – Endocortical; TS – Tibial Shaft; NL – Normal-loaded; UL – Under-loaded; pQCT – Peripheral Quantitative Computed Tomography; DRD – Distal Radius Diaphysis; PTD – Proximal Tibial Diaphysis; LV – 5^th^ Lumbar Vertebra; CVB – Caudal Vertebral Body; LVB – Lumbar Vertebral Body.
