# Supplementary material for: Antisclerostin Effect on Osseointegration and Bone Remodeling
Source: J Clin Med. 2023 Feb 6;12(4):1294. doi: 10.3390/jcm12041294 (PMC9964545; doi:10.3390/jcm12041294)
Supplement: Supplementary file 1 [file jcm-12-01294-s001.zip › Suppl. Table 12.docx]

Table S12. Bone remodeling/formation parameters - part VI.

|  | Sample Size  (Initial) | | | Sample Size  (Final) | | | Drug/Control | Dosage &  Administration Route | ES/BS | | Oc.S/BS | Oc.N/BS | | Fat Cell Volume |
| --- | --- | --- | --- | --- | --- | --- | --- | --- | --- | --- | --- | --- | --- | --- |
| Liu *et al.*  (2018) [57] | 50 | 40 OVX | | 50 | 40 OVX | | Scl-Ab VI | 18.2mg/kg sc. twice week | significantly lower than OVX-vehicle | | - | - | | - |
|  |  |  |  |  |  |  | Scl-Ab VI + DAB | 18.1mg/kg sc. + 18.1mg/kg sc. twice week |  |  | - | - | | - |
|  |  |  |  |  |  |  | saline vehicle | - | higher in alveolar and basal bone than Sham group | | - | - | | - |
|  |  | 10 Sham | |  | 10 Sham | | saline vehicle | - | - | | - | - | | - |
|  | 45 | | | 45 | | | Scl-Ab VI | 25mg/kg sc. twice week | - | | - | - | | - |
|  |  |  |  |  |  |  | Scl-Ab VI + DAB | 25mg/kg sc. +  25mg/kg sc. twice week | - | | - | - | | - |
|  |  |  |  |  |  |  | saline vehicle | - | - | | - | - | | - |
| Wu *et al.*  (2018) [60] | 40 OVX | | | 40 OVX | | | Scl-Ab | 25mg/kg sc. twice week | - | | - | - | | - |
|  |  |  |  |  |  |  | PTH 1-34 | 60𝜇g/kg sc. thrice week | - | | - | - | | - |
|  |  |  |  |  |  |  | Scl-Ab +  PTH 1-34 | 25mg/kg sc. twice week + 60𝜇g/kg sc thrice week | - | | - | - | | - |
|  |  |  |  |  |  |  | vehicle | - | - | | - | - | | - |
| Taut *et al.*  (2013) [65] | 69 | | | 69 | | | EP: Scl-Ab III | 25 mg/kg sc. twice week | - | | - | - | | - |
|  |  |  |  |  |  |  |  | 15 𝜇L of 35.6mg/mL solution locally twice week | - | | - | - | | - |
|  |  |  |  |  |  |  | EP: vehicle | - | - | | - | - | | - |
|  |  |  |  |  |  |  | healthy: PBS | - | - | | - | - | | - |
| Virk *et al.*  (2013) [58] | 72 | | | 72 | | | Scl-Ab III | 25mg/kg sc. twice week | - | | - | - | | - |
|  |  |  |  |  |  |  | PBS | - | - | | - | - | | - |
|  | 30 | | | 30 | | | Scl-Ab III | 25mg/kg | - | | - | - | | - |
|  |  |  |  |  |  |  | PBS | - | - | | - | - | | - |
| McDonald *et al.* (2012) [33] | 132 | | 66 Sham | 127 | | | Scl-Ab III | 25mg/kg sc. twice week | ^-^ | | - | **Center** | 2 weeks: 0.002 ± 0.001 N/mm  3 weeks: 0.002 ± 0.001 N/mm | - |
|  |  |  |  |  |  |  |  |  |  |  |  | **Cortical** | 2 weeks: 0.002 ± 0.001 N/mm  3 weeks: 0.002 ± 0.001 N/mm |  |
|  |  |  |  |  |  |  | saline solution | - | ^-^ | | - | **Center** | 2 weeks: 0.004 ± 0.001 N/mm  3 weeks: 0.002 ± 0.001 N/mm | - |
|  |  |  |  |  |  |  |  |  |  |  |  | **Cortical** | 2 weeks: 0.004 ± 0.001 N/mm  3 weeks: 0.002 ± 0.001 N/mm |  |
|  |  |  | 66 OVX |  |  |  | Scl-Ab III | 25mg/kg sc. twice week | ^-^ | | - | **Center** | 2 weeks: 0.002 ± 0.001 N/mm  3 weeks: 0.002 ± 0.001 N/mm | - |
|  |  |  |  |  |  |  |  |  |  |  |  | **Cortical** | 2 weeks: 0.003± 0.001 N/mm  3 weeks: 0.002 ± 0.001 N/mm |  |
|  |  |  |  |  |  |  | saline solution | - | ^-^ | | - | **Center** | 2 weeks: 0.002 ± 0.001 N/mm  3 weeks: 0.002 ± 0.001 N/mm | - |
|  |  |  |  |  |  |  |  |  |  |  |  | **Cortical** | 2 weeks: 0.003± 0.001 N/mm  3 weeks: 0.001 ± 0.001 N/mm |  |
| Ominsky *et al.*  (2011) [59] | 35 | | | 32 | | | Scl-Ab III | 25mg/kg sc. twice week | - | | - | - | |  |
|  |  |  |  |  |  |  | vehicle | - | - | | - | - | |  |
| Tian *et al*.  (2011) [34] | 67 | | | 67 | | | **Baseline** | | PTM: 3.2 ± 1.1 %  Ec.TS: 3.3 ± 1.0 % | | - | - | | - |
|  |  |  |  |  |  |  | Scl-Ab III | 5mg/kg sc. twice week | **NL** | PTM: 1.7 ± 0.6 %  Ec.TS: 0.8 ± 0.4 % | - | - | | - |
|  |  |  |  |  |  |  |  |  | **UL** | PTM: 3.2 ± 1.0 %  Ec.TS: 1.2 ± 1.2 % |  |  |  |  |
|  |  |  |  |  |  |  |  | 25mg/kg sc. twice week | **NL** | PTM: 0.8 ± 0.3 %  Ec.TS: 0.3 ± 0.2 % | - | - | | - |
|  |  |  |  |  |  |  |  |  | **UL** | PTM: 2.7 ± 0.9 %  Ec.TS: 0.5 ± 0.2 % |  |  |  |  |
|  |  |  |  |  |  |  | saline solution | - | **NL** | PTM: 3.4 ± 0.8 %  Ec.TS: 3.6 ± 1.2 % | - | - | | - |
|  |  |  |  |  |  |  |  |  | **UL** | PTM: 4.7 ± 0.8 %  Ec.TS: 4.4 ± 2.5 % |  |  |  |  |
| Li *et al.*  (2010) [38] | 28 | | | 26 | | | Scl-Ab III | 25mg/kg sc. twice week | - | | PT: 3.7 ± 0.9% | - | | - |
|  |  |  |  |  |  |  |  | 5mg/kg sc. twice week | **-** | | PT: 3.1 ± 0.8% | - | | - |
|  |  |  |  |  |  |  | vehicle | - | - | | PT: 2.5 ± 0.2% | - | | - |
| Ominsky *et al.*  (2010) [64] | 12 | | | 12 | | | Scl-Ab IV | 3mg/kg sc. once month | - | | - | - | | - |
|  |  |  |  |  |  |  |  | 10mg/kg sc. Once month | - | | - | - | | - |
|  |  |  |  |  |  |  |  | 30mg/kg sc. once month | - | | - | - | | - |
|  |  |  |  |  |  |  | vehicle | - | **-** | | - | - | | - |
| Tian *et al.*  (2010) [62] | 32 | | | 32 | | | **Baseline** | | CVB: 1.3 ± 0.5 %  LVB: 3.6 ± 0.7 % | | - | - | | CVB: ∼ 100 ± 0 %  LVB: 3.8 ± 2.2 % |
|  |  |  |  |  |  |  | Scl-Ab III | 5mg/kg sc. twice week | CVB: 1.2 ± 0.4 %  LVB: 1.7 ± 0.3 % | | - | - | | CVB: ∼ 100 ± 0 %  LVB: 4.4 ± 1.8 % |
|  |  |  |  |  |  |  |  | 25mg/kg sc. twice week | CVB: 1.0 ± 0.3 %  LVB: 0.7 ± 0.2 % | | - | - | | CVB: ∼ 100 ± 0 %  LVB: 3.1 ± 1.5 % |
|  |  |  |  |  |  |  | saline solution | - | CVB: 1.4 ± 0.3 %  LVB: 4.1 ± 0.8 % | | - | - | | CVB: ∼ 100 ± 0 %  LVB: 5.5 ± 3.6 % |
| Saag *et al.*  (2017) [67] | 4093 | | | 3150 | | | Romosozumab → alendronate | 210mg sc. once month → 70mg po. once week | - | | - | - | | - |
|  |  |  |  |  |  |  | alendronate → alendronate | 70mg po. once week → 70mg po. once week | - | | - | - | | - |
| McClung *et al.*  (2014) [41] | 419 | | | 383 | | | Romosozumab | 140mg sc. every 3 months | - | | - | - | | - |
|  |  |  |  |  |  |  |  | 210mg sc. every 3 months | - | | - | - | | - |
|  |  |  |  |  |  |  |  | 70mg sc. once month | - | | - | - | | - |
|  |  |  |  |  |  |  |  | 140mg sc. once month | - | | - | - | | - |
|  |  |  |  |  |  |  |  | 210mg sc. once month | - | | - | - | | - |
|  |  |  |  |  |  |  | alendronate | 70 mg po. once week | - | | - | - | | - |
|  |  |  |  |  |  |  | teriparatide | 20𝜇g sc. once day | - | | - | - | | - |
|  |  |  |  |  |  |  | placebo | - | - | | - | - | | - |
| Padhi *et al.*  (2014) [43] | 48 | | 32 women | 46 | | 31 women | romosozumab | 1mg/kg sc. every 2 weeks | - | | - | - | | - |
|  |  |  |  |  |  |  |  | 2mg/kg sc. every 4 weeks | - | | - | - | | - |
|  |  |  |  |  |  |  |  | 2mg/kg sc. every 2 weeks | - | | - | - | | - |
|  |  |  |  |  |  |  |  | 3mg/kg sc. every 4 weeks | - | | - | - | | - |
|  |  |  |  |  |  |  | placebo | - | - | | - | - | | - |
|  |  |  | 16 men |  |  | 15 men |  |  |  |  |  |  |  |  |
|  |  |  |  |  |  |  | romosozumab | 1mg/kg sc. every 2 weeks | - | | - | - | | - |
|  |  |  |  |  |  |  |  | 3mg/kg sc. every 4 weeks | - | | - | - | | - |

ES/BS – Eroded Surface; Oc.S/BS – Osteoclast Surface; Oc.N/BS – Number of TRAP-positive Cells per Bone Surface; PTM – Proximal Tibia Metaphysis; Ec – Endocortical; PT – Proximal Tibia; TS – Tibial Shaft; NL – Normal-loaded; UL – Under-loaded; CVB – Caudal Vertebral Body; LVB – Lumbar Vertebral Body.
