# Supplementary material for: Antisclerostin Effect on Osseointegration and Bone Remodeling
Source: J Clin Med. 2023 Feb 6;12(4):1294. doi: 10.3390/jcm12041294 (PMC9964545; doi:10.3390/jcm12041294)
Supplement: Supplementary file 1 [file jcm-12-01294-s001.zip › Suppl. Table 13.docx]

Table S13. Bone remodeling/formation biomarkers.

|  | Sample Size  (Initial) | | Sample Size  (Final) | | Drug/Control | Dosage &  Administration Route | BSAP | | | Osteocalcin | | | P1NP | | | |
| --- | --- | --- | --- | --- | --- | --- | --- | --- | --- | --- | --- | --- | --- | --- | --- | --- |
| Liu *et al.*  (2018) [57] | 50 | 40 OVX | 50 | 40 OVX | Scl-Ab VI | 18.2mg/kg sc twice week | higher increase than both control (Sham & OVX) | | | - | | | - | | | |
|  |  |  |  |  | Scl-Ab VI + DAB | 18.1mg/kg sc + 18.1mg/kg sc twice week | higher increase than both control (Sham & OVX) | | | - | | | - | | | |
|  |  |  |  |  | saline vehicle | - | - | | | - | | | - | | | |
|  |  | 10 Sham |  | 10 Sham | saline vehicle | - | - | | | - | | | - | | | |
|  | 45 | | 45 | | Scl-Ab VI | 25mg/kg sc twice week | - | | | 98.6 ± 8.0 ng/mL | | | 91.4 ± 8.4 ng/mL | | | |
|  |  |  |  |  | Scl-Ab VI + DAB | 25mg/kg sc + 25mg/kg sc twice week | - | | | 91.4 ± 8.4 ng/mL | | | 29.94 ± 2.30 ng/mL | | | |
|  |  |  |  |  | saline vehicle | - | - | | | Intact: 79.5 ± 2.1 ng/mL  Extracted: 75.1 ± 5.3 ng/mL | | | Intact: 25.15 ± 0.84 ng/mL  Extracted: 23.76 ± 1.63 ng/mL | | | |
| Wu *et al.*  (2018) [60] | 40 OVX | | 40 OVX | | Scl-Ab | 25mg/kg sc twice week |  | | | **12 wks** | higher increase vs vehicle  no difference vs PTH | | **12 wks** | | higher increase vs vehicle  no difference vs PTH | |
|  |  |  |  |  | PTH 1-34 | 60𝜇g/kg sc thrice week |  | | | **12 wks** | higher increase vs vehicle  no difference vs Scl-Ab | | **12 wks** | | higher increase vs vehicle  no difference vs Scl-Ab | |
|  |  |  |  |  | Scl-Ab +  PTH 1-34 | 25mg/kg sc twice week + 60𝜇g/kg sc thrice week |  | | | **12 wks** | sig. higher increase vs all groups | | **12 wks** | | sig. higher increase vs all groups | |
|  |  |  |  |  | vehicle | - | - | | | - | | | - | | | |
| Taut *et al.*  (2013) [65] | 69 | | 69 | | EP: Scl-Ab III | 25 mg/kg sc twice week | - | | | **3 wks** | sig. higher increase compared to vehicle EP and PSB healthy | | **3 wks** | | increase compared to vehicle EP and PSB healthy | |
|  |  |  |  |  |  |  |  |  |  | **6 wks** | maintenance of higher values compared to PBS healthy group | | **6 wks** | | no statistical differences between vehicle EP and PSB healthy | |
|  |  |  |  |  |  | 15 𝜇L of 35.6mg/mL solution locally twice week | - | | | - | | | - | | | |
|  |  |  |  |  | EP: vehicle | - | - | | | - | | | - | | | |
|  |  |  |  |  | healthy: PBS | - | - | | | - | | | - | | | |
| Virk *et al.*  (2013) [58] | 72 | | 72 | | Scl-Ab III | 25mg/kg sc twice week | - | | | - | | | - | | | |
|  |  |  |  |  | PBS | - | - | | | - | | | - | | | |
|  | 30 | | 30 | | Scl-Ab III | 25mg/kg | - | | | **6 wk** | significantly greater than control | | **12 wk** | | significantly greater than control | |
|  |  |  |  |  | PBS | - | - | | | - | | | - | | | |
| McDonald *et al.* (2012) [33] | 132 | 66 Sham | 127 | | Scl-Ab III | 25mg/kg sc twice week | - | | | - | | | - | | | |
|  |  |  |  |  | saline solution | - | - | | | - | | | - | | | |
|  |  | 66 OVX |  |  | Scl-Ab III | 25mg/kg sc twice week | - | | | - | | | - | | | |
|  |  |  |  |  | saline solution | - | - | | | - | | | - | | | |
| Ominsky *et al.*  (2011) [59] | 35 | | 32 | | Scl-Ab III | 25mg/kg sc twice week | - | | | 90.0 ± 4.6 ng/mL | | | 16.0 ± 4.0 ng/mL | | | |
|  |  |  |  |  | vehicle | - | - | | | 79.1 ± 2.1 ng/mL | | | 13.2 ± 0.8 ng/mL | | | |
| Tian *et al*.  (2011) [34] | 67 | | 67 | | Scl-Ab III | 5mg/kg sc twice week | - | | | - | | | - | | | |
|  |  |  |  |  |  | 25mg/kg sc twice week | - | | | - | | | - | | | |
|  |  |  |  |  | saline solution | - | - | | | - | | | - | | | |
| Li *et al.*  (2010) [38] | 28 | | 26 | | Scl-Ab III | 25mg/kg sc twice week | - | | | Baseline: 33.4 ± 2.0 ng/mL  Week1: 92.3 ± 8.6 ng/mL  Week3: 66.6 ± 5.0 ng/mL  Week5: 62.8 ± 4.1 ng/mL | | | - | | | |
|  |  |  |  |  |  | 5mg/kg sc twice week | **-** | | | Baseline: 29.5 ± 3.1 ng/mL  Week1: 72.4 ± 8.3 ng/mL  Week3: 51.3 ± 3.1 ng/mL  Week5: 46.7 ± 2.4 ng/mL | | | - | | | |
|  |  |  |  |  | vehicle | - | - | | | Baseline: 32.4 ± 1.9 ng/mL  Week1: 35.1 ± 2.6 ng/mL  Week3: 33.1 ± 1.6 ng/mL  Week5: 34.5 ± 2.3 ng/mL | | | - | | | |
| Ominsky *et al.*  (2010) [64] | 12 | | 12 | | Scl-Ab IV | 3mg/kg sc once month | - | | | - | | | - | | | |
|  |  |  |  |  |  | 10mg/kg sc once month | - | | | - | | | - | | | |
|  |  |  |  |  |  | 30mg/kg sc once month | - | | | - | | | - | | | |
|  |  |  |  |  | vehicle | - | **-** | | | - | | | - | | | |
| Tian *et al.*  (2010) [62] | 32 | | 32 | | Scl-Ab III | 5mg/kg sc twice week | - | | | - | | | - | | | |
|  |  |  |  |  |  | 25mg/kg sc twice week | - | | | - | | | - | | | |
|  |  |  |  |  | saline solution | - | - | | | - | | | - | | | |
| Saag *et al.*  (2017) [67] | 4093 | | 3150 | | Romosozumab → Alendronate | 210mg sc once month → 70mg po once week | - | | | - | | | **12 mo** | | levels increased vs. control | |
|  |  |  |  |  |  |  |  |  |  |  |  |  | **36 mo** | | levels decreased and maintained below baseline | |
|  |  |  |  |  | Alendronate → Alendronate | 70mg po once week → 70mg po once week | - | | | - | | | levels decreased since the 1^st^ month, remaining below baseline at 36 months | | | |
| McClung *et al.*  (2014) [41] | 419 | | 383 | | Romosozumab | 140mg sc every 3 months | - | | | - | | | **Baseline** | | | 49 (38, 67) 𝜇g/L |
|  |  |  |  |  |  |  | **%Change** | **1 wk** | 15.6 (8.2, 21.8) % | **%Change** | **1 wk** | 8.1 (−2.0, 20.4) % | **%Change** | **1 wk** | | 51.2 (37.6, 87.4) % |
|  |  |  |  |  |  |  |  | **1 mo** | 33.1 (18.7, 51.2) % |  | **1 mo** | 64.1 (38.5, 88.2) % |  | **1 mo** | | 61.6 (23.8, 104.7) % |
|  |  |  |  |  |  |  |  | **2 mo** | −9.7 (−20.2, 7.3) % |  | **2 mo** | 7.1 (−5.7, 32.5) % |  | **2 mo** | | −13.8 (−23.9, 1.0) % |
|  |  |  |  |  |  |  |  | **3 mo** | −18.7 (−30.1, −3.8) % |  | **3 mo** | −6.4 (−19.9, 19.6) % |  | **3 mo** | | −17.4 (−22.5, −4.5) % |
|  |  |  |  |  |  |  |  | **6 mo** | −20.9 (−27.4, −1.5) % |  | **6 mo** | −9.7 (−30.7, 2.3) % |  | **6 mo** | | −21.4 (−34.7, −11.5) % |
|  |  |  |  |  |  |  |  | **9 mo** | −14.1 (−27.4, 6.0) % |  | **9 mo** | −29.2 (−40.1, −17.3) % |  | **9 mo** | | −25.4 (−32.6, −15.9) % |
|  |  |  |  |  |  |  |  | **12 mo** | −14.2 (−26.8, 3.3) % |  | **12 mo** | −26.8 (−36.2, −11.7) % |  | **12 mo** | | −24.4 (−38.8, −4.9) % |
|  |  |  |  |  |  | 210mg sc every 3 months | - | | | - | | | **Baseline** | | | 49 (40, 62) 𝜇g/L |
|  |  |  |  |  |  |  | **%Change** | **1 wk** | 17.5 (10.7, 25.1) % | **%Change** | **1 wk** | 13.0 (2.5, 22.2) % | **%Change** | **1 wk** | | 77.5 (43.2, 98.3) % |
|  |  |  |  |  |  |  |  | **1 mo** | 46.5 (21.5, 72.1) % |  | **1 mo** | 84.3 (54.1, 102.2) % |  | **1 mo** | | 74.8 (46.5, 114.0) % |
|  |  |  |  |  |  |  |  | **2 mo** | −5.2 (−16.8, 10.2) % |  | **2 mo** | 20.0 (−0.3, 34.4) % |  | **2 mo** | | −22.1 (−34.4, -9.1) % |
|  |  |  |  |  |  |  |  | **3 mo** | −19.0 (−25.8, −7.0) % |  | **3 mo** | −5.0 (−17.6, 18.8) % |  | **3 mo** | | −18.1 (−31.7, −4.1) % |
|  |  |  |  |  |  |  |  | **6 mo** | −22.0 (−29.3, −5.5) % |  | **6 mo** | −21.8 (−41.0, −15.5) % |  | **6 mo** | | −25.7 (−36.0, −8.2) % |
|  |  |  |  |  |  |  |  | **9 mo** | −18.4 (−27.7, −6.3) % |  | **9 mo** | −24.8 (−41.4, 2.4) % |  | **9 mo** | | −24.7 (−41.6, −14.1) % |
|  |  |  |  |  |  |  |  | **12 mo** | −10.1 (−26.2, 3.4) % |  | **12 mo** | −22.2 (−36.6, −8.1) % |  | **12 mo** | | −28.5 (−40.0, −5.0) % |
|  |  |  |  |  |  | 70mg sc once month | - | | | - | | | **Baseline** | | | 50 (36, 61) 𝜇g/L |
|  |  |  |  |  |  |  | **%Change** | **1 wk** | 5.5 (0.9, 15.9) % | **%Change** | **1 wk** | 1.5 (−8.8, 10.7) % | **%Change** | **1 wk** | | 31.5 (19.4, 55.6) % |
|  |  |  |  |  |  |  |  | **1 mo** | 10.2 (0.8, 27.7) % |  | **1 mo** | 28.9 (5.2, 46.3) % |  | **1 mo** | | 22.0 (7.1, 40.7) % |
|  |  |  |  |  |  |  |  | **2 mo** | −0.2 (−11.6, 31.0) % |  | **2 mo** | 11.1 (−0.3, 34.4) % |  | **2 mo** | | −0.3 (−17.0, 12.4) % |
|  |  |  |  |  |  |  |  | **3 mo** | −5.7 (−22.0, 9.4) % |  | **3 mo** | 0.9 (−16.0, 22.6) % |  | **3 mo** | | −8.2 (−20.1, 13.4) % |
|  |  |  |  |  |  |  |  | **6 mo** | −9.9 (−21.3, 8.2) % |  | **6 mo** | −8.6 (−33.1, 21.1) % |  | **6 mo** | | −18.5 (−32.6, −4.2) % |
|  |  |  |  |  |  |  |  | **9 mo** | −3.1 (−23.0, 13.6) % |  | **9 mo** | −24.2 (−39.1, −2.3) % |  | **9 mo** | | −25.5 (−43.1, −8.1) % |
|  |  |  |  |  |  |  |  | **12 mo** | −6.9 (−20.2, 14.0) % |  | **12 mo** | −29.2 (−40.4, −8.0) % |  | **12 mo** | | −26.5 (−43.9, −7.2) % |
|  |  |  |  |  |  | 140mg sc once month | - | | | - | | | **Baseline** | | | 48 (38, 56) 𝜇g/L |
|  |  |  |  |  |  |  | **%Change** | **1 wk** | 14.0 (7.5, 21.0) % | **%Change** | **1 wk** | 7.4 (−4.6, 19.6 ) % | **%Change** | **1 wk** | | 56.6 (42.2, 79.5) % |
|  |  |  |  |  |  |  |  | **1 mo** | 36.6 (14.0, 51.2) % |  | **1 mo** | 58 (37.0, 82.4) % |  | **1 mo** | | 68.6 (23.1, 96.4) % |
|  |  |  |  |  |  |  |  | **2 mo** | 15.7 (−1.1, 31.8) % |  | **2 mo** | 35.8 (17.5, 53.8) % |  | **2 mo** | | 9.7 (−13.6, 34.8) % |
|  |  |  |  |  |  |  |  | **3 mo** | 4.0 (−12.8, 22.3) % |  | **3 mo** | 19.3 (−0.5, 41.0) % |  | **3 mo** | | 2.3 (−18.3, 27.8) % |
|  |  |  |  |  |  |  |  | **6 mo** | −7.9 (−16.6, 11.9) % |  | **6 mo** | −5.3 (−27.7, 24.0) % |  | **6 mo** | | −16.4 (−30.2, 8.0) % |
|  |  |  |  |  |  |  |  | **9 mo** | −8.3 (−19.0, 11.0) % |  | **9 mo** | −28.4 (−44.0, −13.9) % |  | **9 mo** | | −26.2 (−37.4, −5.1) % |
|  |  |  |  |  |  |  |  | **12 mo** | −4.6 (−18.5, 11.1) % |  | **12 mo** | −32.9 (−44.3, −17.6) % |  | **12 mo** | | −32.3 (−43.4, −14.3) % |
|  |  |  |  |  |  | 210mg sc once month | - | | | - | | | **Baseline** | | | 53 (42, 64) 𝜇g/L |
|  |  |  |  |  |  |  | **%Change** | **1 wk** | 17.9 (7.8, 27.1) % | **%Change** | **1 wk** | 4.3 (−0.2, 17.1) % | **%Change** | **1 wk** | | 82.7 (64.7, 101.1) % |
|  |  |  |  |  |  |  |  | **1 mo** | 51.8 (40.8, 82.1) % |  | **1 mo** | 78.3 (54.1, 107.0) % |  | **1 mo** | | 91.2 (56.8, 126.7) % |
|  |  |  |  |  |  |  |  | **2 mo** | 36.7 (12.9, 52.6) % |  | **2 mo** | 58.2 (29.1, 99.5) % |  | **2 mo** | | 36.4 (8.8, 73.8) % |
|  |  |  |  |  |  |  |  | **3 mo** | 26.6 (8.1, 47.7) % |  | **3 mo** | 45.8 (19.3, 88.6) % |  | **3 mo** | | 31.8 (−3.8, 63.7) % |
|  |  |  |  |  |  |  |  | **6 mo** | 18.2 (−0.4, 35.7) % |  | **6 mo** | 13.5 (−13.7, 37.1) % |  | **6 mo** | | 6.3 (−18.9, 32.3) % |
|  |  |  |  |  |  |  |  | **9 mo** | 6.8 (−9.1, 22.0) % |  | **9 mo** | 1.1 (−25.6, 19.0) % |  | **9 mo** | | −10.4 (−31.4, 12.9) % |
|  |  |  |  |  |  |  |  | **12 mo** | 2.5 (−7.6, 21.1) % |  | **12 mo** | −15.5 (−24.4, −0.8) % |  | **12 mo** | | −19.8 (−38.0, −1.5) % |
|  |  |  |  |  | alendronate | 70 mg po once week | - | | | - | | | **Baseline** | | | 49 (40, 58) 𝜇g/L |
|  |  |  |  |  |  |  | **%Change** | **3 mo** | −33.0 (−41.8, −20.5) % | **%Change** | **3 mo** | −29.7 (−42.1, −13.6) % | **%Change** | **3 mo** | | −48.3 (−63.7, −34.8) % |
|  |  |  |  |  |  |  |  | **6 mo** | −34.4 (−47.9, −24.3) % |  | **6 mo** | −41.0 (−53.6, −29.2) % |  | **6 mo** | | −59.2 (−70.0, −41.3) % |
|  |  |  |  |  |  |  |  | **9 mo** | −29.3 (−41.9, −20.4) % |  | **9 mo** | −49.9 (−62.8, −39.3) % |  | **9 mo** | | −59.3 (−71.8, −45.8) % |
|  |  |  |  |  |  |  |  | **12 mo** | −31.4 (−43.0, −22.7) % |  | **12 mo** | −49.5 (−59.9, −41.7) % |  | **12 mo** | | −64.2 (−70.3, −44.3) % |
|  |  |  |  |  | teriparatide | 20𝜇g sc once day | - | | | - | | | **Baseline** | | | 49 (42, 67) 𝜇g/L |
|  |  |  |  |  |  |  | **%Change** | **3 mo** | 21.7 (−4.9, 55.5) % | **%Change** | **3 mo** | 95.5 (38.4, 177.4) % | **%Change** | **3 mo** | | 88.8 (43.8, 146.0) % |
|  |  |  |  |  |  |  |  | **6 mo** | 26.2 (2.7, 70.9) % |  | **6 mo** | 87.6 (27.8, 232.8) % |  | **6 mo** | | 120.5 (51.5, 245.3) % |
|  |  |  |  |  |  |  |  | **9 mo** | 34.8 (5.0, 88.3) % |  | **9 mo** | 69.7 (39.8, 204.4) % |  | **9 mo** | | 111.3 (48.4, 233.7) % |
|  |  |  |  |  |  |  |  | **12 mo** | 43.0 (13.5, 79.5) % |  | **12 mo** | 76.6 (20.1, 193.6) % |  | **12 mo** | | 84.2 (48.4, 191.7) % |
|  |  |  |  |  | placebo | - | - | | | - | | | **Baseline** | | | 48 (38, 59) 𝜇g/L |
|  |  |  |  |  |  |  | **%Change** | **1 wk** | 0.7 (−4.9, 7.3) % | **%Change** | **1 wk** | −5.1 (−9.5, 5.4) % | **%Change** | **1 wk** | | −2.2 (−8.4, 5.1) % |
|  |  |  |  |  |  |  |  | **1 mo** | −0.5 (−7.6, 10.1) % |  | **1 mo** | −3.7 (−11.5, 5.7) % |  | **1 mo** | | −4.1 (−12.2, 7.8) % |
|  |  |  |  |  |  |  |  | **2 mo** | −3.4 (−13.2, 8.0) % |  | **2 mo** | −2.5 (−11.2, 16.8) % |  | **2 mo** | | −3.5 (−13.7, 9.5) % |
|  |  |  |  |  |  |  |  | **3 mo** | −0.0 (−18.3, 11.1) % |  | **3 mo** | 3.0 (−11.6, 22.7) % |  | **3 mo** | | −6.9 (−17.2, 8.9) % |
|  |  |  |  |  |  |  |  | **6 mo** | −2.9 (−16.2, 7.9) % |  | **6 mo** | −5.9 (−27.9, 15.0) % |  | **6 mo** | | −12.5 (−21.3, 7.1) % |
|  |  |  |  |  |  |  |  | **9 mo** | 3.9 (−9.9, 16.9) % |  | **9 mo** | −10.8 (−28.3, 20.0) % |  | **9 mo** | | −11.3 (−20.0, 5.1) % |
|  |  |  |  |  |  |  |  | **12 mo** | 11.7 (−2.3, 27.0) % |  | **12 mo** | −12.9 (−26.6, 8.1) % |  | **12 mo** | | −8.5 (−22.3, 15.0) % |
| Padhi *et al.*  (2014) [43] | 48 | 32 women | 46 | 31 women | Romosozumab | 1mg/kg sc every 2 weeks | Baseline 18.00 ± 7.59 𝜇g/L  increase similar as P1NP, but data not reported | | | Baseline: 34.59 ± 24.46 𝜇g/L  increase similar as P1NP, but data not reported | | | Baseline: 66.83 ± 23.54 ng/mL  Max. mean increase: 83 ± 22 % | | | |
|  |  |  |  |  |  | 2mg/kg sc every 4 weeks | Baseline 14.04 ± 3.29 𝜇g/L  increase similar as P1NP, but data not reported | | | Baseline: 25.26 ± 9.94 𝜇g/L  increase similar as P1NP, but data not reported | | | Baseline: 65.00 ± 18.25 ng/mL  Max. mean increase: 66 ± 15 % | | | |
|  |  |  |  |  |  | 2mg/kg sc every 2 weeks | Baseline 15.21 ± 1.71 𝜇g/L  increase similar as P1NP, but data not reported | | | Baseline: 22.73 ± 9.18 𝜇g/L  increase similar as P1NP, but data not reported | | | Baseline: 61.42 ± 14.38 ng/mL  Max. mean increase: 140 ± 18 % | | | |
|  |  |  |  |  |  | 3mg/kg sc every 4 weeks | Baseline 16.40 ± 6.75 𝜇g/L  increase similar as P1NP, but data not reported | | | Baseline: 20.96 ± 8.58 𝜇g/L  increase similar as P1NP, but data not reported | | | Baseline: 59.00 ± 24.45 ng/L  Max. mean increase: 129 ± 21 % | | | |
|  |  |  |  |  | placebo | - | Baseline 14.88 ± 5.02 𝜇g/L  decease similar as P1NP, but data not reported | | | Baseline: 23.09 ± 8.93 𝜇g/L  decrease similar as P1NP, but data not reported | | | Baseline: 54.75 ± 22.77 ng/mL  Max. mean increase: 13 ± 6.8 % | | | |
|  |  | 16 men |  | 15 men |  |  |  |  |  |  |  |  |  |  |  |  |
|  |  |  |  |  | Romosozumab | 1mg/kg sc every 2 weeks | Baseline 12.83 ± 2.10 𝜇g/L  increase similar as P1NP, but data not reported | | | Baseline: 19.92 ± 0.59 𝜇g/L  increase similar as P1NP, but data not reported | | | Baseline: 39.33 ± 8.11 ng/mL  Max. mean increase: 106 ± 15 % | | | |
|  |  |  |  |  |  | 3mg/kg sc every 4 weeks | Baseline 12.68 ± 1.52 𝜇g/L  increase similar as P1NP, but data not reported | | | Baseline: 20.11 ± 5.90 𝜇g/L  increase similar as P1NP, but data not reported | | | Baseline: 40.08 ± 7.63 ng/mL  Max. mean increase: 147 ± 33 % | | | |

BSAP -Bone Specific Alkaline Phosphatase ; P1NP – Procollagen Type 1 N-terminal Propeptide; wk – week; mo – month (s); %Change – Percent change from Baseline.
