# Supplementary material for: Antisclerostin Effect on Osseointegration and Bone Remodeling
Source: J Clin Med. 2023 Feb 6;12(4):1294. doi: 10.3390/jcm12041294 (PMC9964545; doi:10.3390/jcm12041294)
Supplement: Supplementary file 1 [file jcm-12-01294-s001.zip › Suppl. Table 14.docx]

Table S14. Bone remodeling/bone resorption markers.

|  | Sample Size  (Initial) | | Sample Size  (Final) | | Drug/Control | Dosage &  Administration Route | CTX | | | | TRACP-5b | |
| --- | --- | --- | --- | --- | --- | --- | --- | --- | --- | --- | --- | --- |
| Liu *et al.*  (2018) [57] | 50 | 40 OVX | 50 | 40 OVX | Scl-Ab VI | 18.2mg/kg sc twice week | - | | | |  | |
|  |  |  |  |  | Scl-Ab VI + DAB | 18.1mg/kg sc + 18.1mg/kg sc twice week | - | | | |  | |
|  |  |  |  |  | saline vehicle | - | - | | | |  | |
|  |  | 10 Sham |  | 10 Sham | saline vehicle | - | - | | | |  | |
|  | 45 | | 45 | | Scl-Ab VI | 25mg/kg sc twice week | - | | | | 3.52 ± 0.28 U/L | |
|  |  |  |  |  | Scl-Ab VI + DAB | 25mg/kg sc + 25mg/kg sc twice week | - | | | | 2.5 ± 0.17 U/L | |
|  |  |  |  |  | saline vehicle | - | - | | | | Intact: 3.92 ± 0.24 U/L  Extracted: 3.72 ± 0.22 U/L | |
| Wu *et al.*  (2018) [60] | 40 OVX | | 40 OVX | | Scl-Ab | 25mg/kg sc twice week | no significant differences in CTX-1 between all groups. | | | |  | |
|  |  |  |  |  | PTH 1-34 | 60𝜇g/kg sc thrice week |  |  |  |  |  | |
|  |  |  |  |  | Scl-Ab + PTH 1-34 | 25mg/kg sc twice week + 60𝜇g/kg sc thrice week |  |  |  |  |  | |
|  |  |  |  |  | vehicle | - |  |  |  |  |  | |
| Taut *et al.*  (2013) [65] | 69 | | 69 | | EP: Scl-Ab III | 25 mg/kg sc twice week | - | | | | **6 weeks** | no changes vs EP vehicle |
|  |  |  |  |  |  | 15 𝜇L of 35.6mg/mL solution locally twice week | - | | | |  | |
|  |  |  |  |  | EP: vehicle | - | - | | | |  | |
|  |  |  |  |  | healthy: PBS | - | - | | | |  | |
| Virk *et al.*  (2013) [58] | 72 | | 72 | | Scl-Ab III | 25mg/kg sc twice week | - | | | |  | |
|  |  |  |  |  | PBS | - | - | | | |  | |
|  | 30 | | 30 | | Scl-Ab III | 25mg/kg | - | | | | no significant differences between both groups at any time. | |
|  |  |  |  |  | PBS | - | - | | | |  |  |
| McDonald *et al.* (2012) [33] | 132 | 66 Sham | 127 | | Scl-Ab III | 25mg/kg sc twice week | - | | | | - | |
|  |  |  |  |  | saline solution | - | - | | | | - | |
|  |  | 66 OVX |  |  | Scl-Ab III | 25mg/kg sc twice week | - | | | | - | |
|  |  |  |  |  | saline solution | - | - | | | | - | |
| Ominsky *et al.*  (2011) [59] | 35 | | 32 | | Scl-Ab III | 25mg/kg sc twice week | - | | | | - | |
|  |  |  |  |  | vehicle | - | - | | | | - | |
| Tian *et al*.  (2011) [34] | 67 | | 67 | | Scl-Ab III | 5mg/kg sc twice week | - | | | | - | |
|  |  |  |  |  |  | 25mg/kg sc twice week | - | | | | - | |
|  |  |  |  |  | saline solution | - | - | | | | - | |
| Li *et al.*  (2010) [38] | 28 | | 26 | | Scl-Ab III | 25mg/kg sc twice week | no significant effects in CTX-1 | | | | - | |
|  |  |  |  |  |  | 5mg/kg sc twice week |  |  |  |  | - | |
|  |  |  |  |  | vehicle | - | - | | | | - | |
| Ominsky *et al.*  (2010) [64] | 12 | | 12 | | Scl-Ab IV | 3mg/kg sc once month | no significant effects | | | | - | |
|  |  |  |  |  |  | 10mg/kg sc once month |  |  |  |  | - | |
|  |  |  |  |  |  | 30mg/kg sc once month |  |  |  |  | - | |
|  |  |  |  |  | vehicle | - | - | | | | - | |
| Tian *et al.*  (2010) [62] | 32 | | 32 | | Scl-Ab III | 5mg/kg sc twice week | - | | | | - | |
|  |  |  |  |  |  | 25mg/kg sc twice week | - | | | | - | |
|  |  |  |  |  | saline solution | - | - | | | | - | |
| Saag *et al.*  (2017) [67] | 4093 | | 3150 | | Romosozumab → alendronate | 210mg sc once month → 70mg po once week | **12 mo** | | βCTX levels decreased vs control | | - | |
|  |  |  |  |  |  |  | **36 mo** | | βCTX levels decreased and were maintained below baseline | |  |  |
|  |  |  |  |  | alendronate → alendronate | 70mg po once week → 70mg po once week | βCTX levels decreased since the 1^st^ month, remaining below baseline at 36 months | | | |  | |
| McClung *et al.*  (2014) [41] | 419 | | 383 | | Romosozumab | 140mg sc every 3 months | **βCTX** | **Baseline** | | 525 (358, 714) ng/L |  | |
|  |  |  |  |  |  |  |  | **%Change** | **1 wk** | −34.5 (−45.0, −27.6) % |  | |
|  |  |  |  |  |  |  |  |  | **1 mo** | −22.7 (−40.1, 0.7) % |  | |
|  |  |  |  |  |  |  |  |  | **2 mo** | −8.4 (−24.9, 5.8) % |  | |
|  |  |  |  |  |  |  |  |  | **3 mo** | −5.3 (−27.0, 5.4) % |  | |
|  |  |  |  |  |  |  |  |  | **6 mo** | −13.1 (−25.3, 2.7) % |  | |
|  |  |  |  |  |  |  |  |  | **9 mo** | −1.0 (−22.6, 18.8) % |  | |
|  |  |  |  |  |  |  |  |  | **12 mo** | 6.2 (−9.8, 32.8) % |  | |
|  |  |  |  |  |  | 210mg sc every 3 months | **βCTX** | **Baseline** | | 478 (362, 695) ng/L |  | |
|  |  |  |  |  |  |  |  | **%Change** | **1 wk** | −42.0 (−53.3, −27.4) % |  | |
|  |  |  |  |  |  |  |  |  | **1 mo** | −33.6 (−45.0, −21.0) % |  | |
|  |  |  |  |  |  |  |  |  | **2 mo** | −10.4 (−27.6, 12.5) % |  | |
|  |  |  |  |  |  |  |  |  | **3 mo** | −11.5 (−28.9, 6.2) % |  | |
|  |  |  |  |  |  |  |  |  | **6 mo** | −12.6 (−27.1, 18.1) % |  | |
|  |  |  |  |  |  |  |  |  | **9 mo** | −2.3 (−29.9, 12.1) % |  | |
|  |  |  |  |  |  |  |  |  | **12 mo** | −7.1 (−16.9, 18.8) % |  | |
|  |  |  |  |  |  | 70mg sc once month | **βCTX** | **Baseline** | | 486 (374, 627) ng/L |  | |
|  |  |  |  |  |  |  |  | **%Change** | **1 wk** | −33.7 (−42.2, −22.4) % |  | |
|  |  |  |  |  |  |  |  |  | **1 mo** | −22.3 (−31.8, −5.7) % |  | |
|  |  |  |  |  |  |  |  |  | **2 mo** | −14.5 (−28.7, 4.1) % |  | |
|  |  |  |  |  |  |  |  |  | **3 mo** | −17.1 (−29.7, −5.7) % |  | |
|  |  |  |  |  |  |  |  |  | **6 mo** | −10.6 (−34.5, 13.4) % |  | |
|  |  |  |  |  |  |  |  |  | **9 mo** | −17.7 (−33.1, 18.7) % |  | |
|  |  |  |  |  |  |  |  |  | **12 mo** | −18.7 (−37.9, 3.7) % |  | |
|  |  |  |  |  |  | 140mg sc once month | **βCTX** | **Baseline** | | 532 (363, 622) ng/L |  | |
|  |  |  |  |  |  |  |  | **%Change** | **1 wk** | −36.8 (−46.2, −29.2) % |  | |
|  |  |  |  |  |  |  |  |  | **1 mo** | −35.9 (−44.8, −16.3) % |  | |
|  |  |  |  |  |  |  |  |  | **2 mo** | −26.9 (−37.7, −0.0) % |  | |
|  |  |  |  |  |  |  |  |  | **3 mo** | −27.4 (−36.8, −13.3) % |  | |
|  |  |  |  |  |  |  |  |  | **6 mo** | −24.5 (−46.6, 4.1) % |  | |
|  |  |  |  |  |  |  |  |  | **9 mo** | −29.2 (−48.1, −1.5) % |  | |
|  |  |  |  |  |  |  |  |  | **12 mo** | −29.3 (−55.1, −14.5) % |  | |
|  |  |  |  |  |  | 210mg sc once month | **βCTX** | **Baseline** | | 519 (405, 642) ng/L |  | |
|  |  |  |  |  |  |  |  | **%Change** | **1 wk** | −41.4 (−52.5, −32.1) % |  | |
|  |  |  |  |  |  |  |  |  | **1 mo** | −29.5 (−40.6, −12.6) % |  | |
|  |  |  |  |  |  |  |  |  | **2 mo** | −4.9 (−28.7, 15.0) % |  | |
|  |  |  |  |  |  |  |  |  | **3 mo** | 1.2 (−21.7, 19.9) % |  | |
|  |  |  |  |  |  |  |  |  | **6 mo** | −9.6 (−26.8, 11.0) % |  | |
|  |  |  |  |  |  |  |  |  | **9 mo** | −20.4 (−31.9, −1.1) % |  | |
|  |  |  |  |  |  |  |  |  | **12 mo** | −26.3 (−42.1, −8.8) % |  | |
|  |  |  |  |  | alendronate | 70 mg po once week | **βCTX** | **Baseline** | | 494 (373, 614) ng/L |  | |
|  |  |  |  |  |  |  |  | **%Change** | **3 mo** | −65.8 (−84.0, −51.5) % |  | |
|  |  |  |  |  |  |  |  |  | **6 mo** | −65.0 (−76.7, −45.9) % |  | |
|  |  |  |  |  |  |  |  |  | **9 mo** | −66.8 (−75.0, −47.9) % |  | |
|  |  |  |  |  |  |  |  |  | **12 mo** | −65.5 (−82.3, −49.7) % |  | |
|  |  |  |  |  | teriparatide | 20𝜇g sc once day | **βCTX** | **Baseline** | | 506 (410, 690) ng/L |  | |
|  |  |  |  |  |  |  |  | **%Change** | **3 mo** | 58.6 (23.8, 135.2) % |  | |
|  |  |  |  |  |  |  |  |  | **6 mo** | 80.4 (39.7, 167.8) % |  | |
|  |  |  |  |  |  |  |  |  | **9 mo** | 79.7 (15.3, 169.3) % |  | |
|  |  |  |  |  |  |  |  |  | **12 mo** | 79.7 (15.7, 140.5) % |  | |
|  |  |  |  |  | placebo | - | **βCTX** | **Baseline** | | 481 (373, 673) ng/L |  | |
|  |  |  |  |  |  |  |  | **%Change** | **1 wk** | −0.6 (−13.7, 14.4) % |  | |
|  |  |  |  |  |  |  |  |  | **1 mo** | −3.1 (−12.8, 8.8) % |  | |
|  |  |  |  |  |  |  |  |  | **2 mo** | 2.2 (−15.7, 13.6) % |  | |
|  |  |  |  |  |  |  |  |  | **3 mo** | −3.5 (−19.3, 18.0) % |  | |
|  |  |  |  |  |  |  |  |  | **6 mo** | 0.3 (−12.8, 24.4) % |  | |
|  |  |  |  |  |  |  |  |  | **9 mo** | 1.0 (−17.0, 32.5) % |  | |
|  |  |  |  |  |  |  |  |  | **12 mo** | 4.6 (−13.3, 41.5) % |  | |
| Padhi *et al.*  (2014) [43] | 48 | 32 women | 46 | 31 women | Romosozumab | 1mg/kg sc every 2 weeks | **sCTX** | Baseline: 5771.58 ± 2304.36 pmol/L | | |  | |
|  |  |  |  |  |  |  |  | Max. mean decrease: 15 ± 11 % | | |  | |
|  |  |  |  |  |  | 2mg/kg sc every 4 weeks |  | Baseline: 4516.42 ± 1154.62 pmol/L | | |  | |
|  |  |  |  |  |  |  |  | Max. mean decrease: 35 ± 8.7 % | | |  | |
|  |  |  |  |  |  | 2mg/kg sc every 2 weeks |  | Baseline: 4808.58 ± 616.58 pmol/L | | |  | |
|  |  |  |  |  |  |  |  | Max. mean decrease: 38 ± 2.2 % | | |  | |
|  |  |  |  |  |  | 3mg/kg sc every 4 weeks |  | Baseline: 4733.00 ± 1686.35 pmol/L | | |  | |
|  |  |  |  |  |  |  |  | Max. mean decrease: 37 ± 5.4 % | | |  | |
|  |  |  |  |  | placebo | - |  | Baseline: 5651.00 ± 1686.53 pmol/L | | |  | |
|  |  | 16 men |  | 15 men |  |  |  | Max. mean decrease: 13 ± 6.8 % | | |  | |
|  |  |  |  |  | Romosozumab | 1mg/kg sc every 2 weeks |  | Baseline: 3451.00 ± 638.09 pmol/L | | |  | |
|  |  |  |  |  |  |  |  | Max. mean decrease: 42 ± 4.1 % | | |  | |
|  |  |  |  |  |  | 3mg/kg sc every 4 weeks |  | Baseline: 4027.08 ± 1413.24 pmol/L | | |  | |
|  |  |  |  |  |  |  |  | Max. mean decrease: 50 ± 4.8 % | | |  | |

CTX/ sCTX– serum C-Telopeptide ; CTX-1–; βCTX - ;wk – week; mo – month (s); %Change – Percent change from Baseline.
