# Supplementary material for: Antisclerostin Effect on Osseointegration and Bone Remodeling
Source: J Clin Med. 2023 Feb 6;12(4):1294. doi: 10.3390/jcm12041294 (PMC9964545; doi:10.3390/jcm12041294)
Supplement: Supplementary file 1 [file jcm-12-01294-s001.zip › Suppl. Table 15.docx]

Table S15. Bone strength endpoints.

|  | Sample Size  (Initial) | | | Sample Size  (Final) | | | Drug/Control | | | Dosage &  Administration Route | Maximum Load | | | Stiffness | | | Energy to Failure | | Peak Load |
| --- | --- | --- | --- | --- | --- | --- | --- | --- | --- | --- | --- | --- | --- | --- | --- | --- | --- | --- | --- |
| Liu *et al.*  (2018) [57] | 50 | | 40 OVX | 50 | | 40 OVX | Scl-Ab VI | | | 18.2mg/kg sc twice week | - | | | - | | | - | | - |
|  |  |  |  |  |  |  | Scl-Ab VI + DAB | | | 18.1mg/kg sc + 18.1mg/kg sc twice week | - | | | - | | | - | | - |
|  |  |  |  |  |  |  | saline vehicle | | | - | - | | | - | | | - | | - |
|  |  |  | 10 Sham |  |  | 10 Sham | saline vehicle | | | - | - | | | - | | | - | | - |
|  | 45 | | | 45 | | | Scl-Ab VI | | | 25mg/kg sc twice week | - | | | - | | | - | | - |
|  |  |  |  |  |  |  | Scl-Ab VI + DAB | | | 25mg/kg sc + 25mg/kg sc twice week | - | | | - | | | - | | - |
|  |  |  |  |  |  |  | saline vehicle | | | - | - | | | - | | | - | | - |
| Wu *et al.*  (2018) [60] | 40 OVX | | | 40 OVX | | | Scl-Ab | | | 25mg/kg sc twice week | **12 wks** | higher increase vs vehicle  no difference vs PTH | **12 wks** | | higher increase vs vehicle  no difference vs PTH | **12 wks** | | higher increase vs vehicle  no difference vs PTH | - |
|  |  |  |  |  |  |  | PTH 1-34 | | | 60𝜇g/kg sc thrice week | **12 wks** | sig. increase vs vehicle;  no difference vs Scl-Ab | **12 wks** | | significant increase vs vehicle  no difference vs Scl-Ab | **12 wks** | | higher increase vs vehicle  no difference vs Scl-Ab | - |
|  |  |  |  |  |  |  | Scl-Ab +  PTH 1-34 | | | 25mg/kg sc twice week + 60𝜇g/kg sc thrice week | **12 wks** | sig. increase vs vehicle  no difference vs other 2 groups | **12 wks** | | significant increase vs all groups | **12 wks** | | significant increase vs all groups | - |
|  |  |  |  |  |  |  | vehicle | | | - | - | | | - | | | - | | - |
| Taut *et al.*  (2013) [65] | 69 | | | 69 | | | EP: Scl-Ab III | | | 25 mg/kg sc twice week | - | | | - | | | - | | - |
|  |  |  |  |  |  |  |  |  |  | 15 𝜇L of 35.6mg/mL solution locally twice week | - | | | - | | | - | | - |
|  |  |  |  |  |  |  | EP: vehicle | | | - | - | | | - | | | - | | - |
|  |  |  |  |  |  |  | healthy: PBS | | | - | - | | | - | | | - | | - |
| Virk *et al.*  (2013) [58] | 72 | | | 72 | | | Scl-Ab III | | | 25mg/kg sc twice week | - | | | - | | | - | | - |
|  |  |  |  |  |  |  | PBS | | | - | - | | | - | | | - | | - |
|  | 30 | | | 30 | | | Scl-Ab III | | | 25mg/kg | - | | | 6 weeks: significantly greater than control | | | 12 weeks: significantly greater than control | | - |
|  |  |  |  |  |  |  | PBS | | | - | - | | | - | | | - | | - |
| McDonald *et al.* (2012) [33] | 132 | | 66 Sham | 127 | | | Scl-Ab III | | 25mg/kg sc twice week | | - | | | - | | | - | | - |
|  |  |  |  |  |  |  | saline solution | | - | | - | | | - | | | - | | - |
|  |  |  | 66 OVX |  |  |  | Scl-Ab III | | 25mg/kg sc twice week | | - | | | - | | | - | | - |
|  |  |  |  |  |  |  | saline solution | | - | | - | | | - | | | - | | - |
| Ominsky *et al.*  (2011) [59] | 35 | | | 32 | | | Scl-Ab III | | 25mg/kg sc twice week | | - | | | **Fractured Femur:** 48% increase in torsional stiffness compared to vehicle | | | - | | **Intact Femur:**  FD: 223 ± 10 N |
|  |  |  |  |  |  |  |  |  |  |  |  |  |  | **Intact Femur:** FD: 637 ± 37 N/mm | | |  |  |  |
|  |  |  |  |  |  |  | vehicle | | - | | - | | | FD: 570 ± 22 N/mm | | | - | | FD: 191 ± 8 N |
| Tian *et al*.  (2011) [34] | 67 | | | 67 | | | Scl-Ab III | | 5mg/kg sc twice week | | - | | | - | | | - | | - |
|  |  |  |  |  |  |  |  |  | 25mg/kg sc twice week | | - | | | - | | | - | | - |
|  |  |  |  |  |  |  | saline solution | | - | | - | | | - | | | - | | - |
| Li *et al.*  (2010) [38] | 28 | | | 26 | | | Scl-Ab III | | 25mg/kg sc twice week | | LV: 693 ± 37 N  FD: 249 ± 13 N  FN: 247 ± 12 N | | | LV: 4623 ± 549 N/mm  FD: 781 ± 53 N/mm  FN: 689 ± 56 N/mm | | | LV: 82.6 ± 10.0 mJ  FD: 172 ± 22 mJ  FN: 68.6 ± 9.5 mJ | | - |
|  |  |  |  |  |  |  |  |  | 5mg/kg sc twice week | | LV: 467 ± 42 N  FD: 254 ± 13 N  FN: 241 ± 12 N | | | LV: 3292 ± 379 N/mm  FD: 770 ± 61 N/mm  FN: 805 ± 70 N/mm | | | LV: 59.9 ± 5.7 mJ  FD: 148 ± 16 mJ  FN: 35.7 ± 5.8 mJ | | - |
|  |  |  |  |  |  |  | vehicle | | - | | LV: 349 ± 28 N  FD: 190 ± 12 N  FN: 201 ± 8 N | | | LV: 2710 ± 299 N/mm  FD: 680 ± 35 N/mm  FN: 611 ± 20 N/mm | | | LV: 38.9 ± 4.9 mJ  FD: 139 ± 13 mJ  FN: 46.2 ± 5.6 mJ | | - |
| Ominsky *et al.*  (2010) [64] | 12 | | | 12 | | | Scl-Ab IV | | 3mg/kg sc once month | | - | | | FD: 838 ± 106 N/mm | | | FD: 2523 N×mm | | FD: 917 ± 121 N |
|  |  |  |  |  |  |  |  |  | 10mg/kg sc once month | | - | | | FD: 873 ± 84 N/mm | | | FD: 3190 ± 743 N×mm | | FD: 1005 ± 81 N |
|  |  |  |  |  |  |  |  |  | 30mg/kg sc once month | | - | | | FD: 1040 ± 192 N/mm | | | FD: 4994 ± 904 N×mm | | FD: 1285 ± 241 N |
|  |  |  |  |  |  |  | vehicle | | - | | **-** | | | FD: 888 ± 106 N/mm | | | FD: 3600 ± 282 N×mm | | FD: 1008 ± 102 N |
| Tian *et al.*  (2010) [62] | 32 | | | 32 | | | Scl-Ab III | | 5mg/kg sc twice week | | - | | | - | | | - | | - |
|  |  |  |  |  |  |  |  |  | 25mg/kg sc twice week | | - | | | - | | | - | | - |
|  |  |  |  |  |  |  | saline solution | | - | | - | | | - | | | - | | - |
| Saag *et al.*  (2017) [67] | 4093 | | | 3150 | | | Romosozumab → Alendronate | | | 210mg sc once month → 70mg po once week | - | | | - | | | - | | - |
|  |  |  |  |  |  |  | Alendronate → Alendronate | | | 70mg po once week → 70mg po once week | - | | | - | | | - | | - |
| McClung *et al.*  (2014) [41] | 419 | | | 383 | | | Romosozumab | | | 140mg sc every 3 months | - | | | - | | | - | | - |
|  |  |  |  |  |  |  |  |  |  | 210mg sc every 3 months | - | | | - | | | - | | - |
|  |  |  |  |  |  |  |  |  |  | 70mg sc once month | - | | | - | | | - | | - |
|  |  |  |  |  |  |  |  |  |  | 140mg sc once month | - | | | - | | | - | | - |
|  |  |  |  |  |  |  |  |  |  | 210mg sc once month | - | | | - | | | - | | - |
|  |  |  |  |  |  |  | Alendronate | | | 70 mg po once week | - | | | - | | | - | | - |
|  |  |  |  |  |  |  | Teriparatide | | | 20𝜇g sc once day | - | | | - | | | - | | - |
|  |  |  |  |  |  |  | placebo | | | - | - | | | - | | | - | | - |
| Padhi *et al.*  (2014) [43] | 48 | 32 women | | 46 | 31 women | | | Romosozumab | | 1mg/kg sc every 2 weeks | - | | | - | | | - | | - |
|  |  |  |  |  |  |  |  |  |  | 2mg/kg sc every 4 weeks | - | | | - | | | - | | - |
|  |  |  |  |  |  |  |  |  |  | 2mg/kg sc every 2 weeks | - | | | - | | | - | | - |
|  |  |  |  |  |  |  |  |  |  | 3mg/kg sc every 4 weeks | - | | | - | | | - | | - |
|  |  |  |  |  |  |  |  | placebo | | - | -  - | | | -  - | | | -  - | | -  - |
|  |  | 16 men | |  | 15 men | | |  |  |  |  |  |  |  |  |  |  |  |  |
|  |  |  |  |  |  |  |  | Romosozumab | | 1mg/kg sc every 2 weeks | - | | | - | | | - | | - |
|  |  |  |  |  |  |  |  |  |  | 3mg/kg sc every 4 weeks | - | | | - | | | - | | - |

FD – Femoral Diaphysis; LV - 5^th^ Lumbar Vertebra; FN – Femoral Neck.
