# Supplementary material for: Antisclerostin Effect on Osseointegration and Bone Remodeling
Source: J Clin Med. 2023 Feb 6;12(4):1294. doi: 10.3390/jcm12041294 (PMC9964545; doi:10.3390/jcm12041294)
Supplement: Supplementary file 1 [file jcm-12-01294-s001.zip › Suppl. Table 2.docx]

Table S2. Osseointegration/Bone formation parameters – Part II.

|  | Sample Size  (Initial) | | Sample Size  (Final) | | Drug/Control | Dosage & Administration Route | Implant | Ct.Ar | M.Ar | Tt.Ar | Bone Fill |
| --- | --- | --- | --- | --- | --- | --- | --- | --- | --- | --- | --- |
| Korn *et al.*  (2019) [61] | 128 | | 124 | | sclerostin antibody | 100mg/kg iv once week | reference-coated implant | - | - | - | - |
|  |  |  |  |  |  |  | ZOL-coated implant | - | - | - | - |
|  |  |  |  |  | non antibody applied | - | reference-coated implant | - | - | - | - |
|  |  |  |  |  |  |  | ZOL-coated implant | - | - | - | - |
| Yu *et al.*  (2018) [40] | 60 | | 60 | | Scl-Ab | 25mg/kg sc | cp-Ti, solid cylinder implants with titanium plasma-sprayed surface | - | - | - | 28 days: significantly greater than control |
|  |  |  |  |  | PBS | - |  | - | - | - | - |
| Virdi *et al.*  (2015) [35] | 144 | 72 OVX | 142 | 71 OVX | Scl-Ab III | 25 mg/kg sc twice week | cp-Ti, dual acid-etched surface | - | - | - | - |
|  |  |  |  |  | vehicle | - |  | - | - | - | - |
|  |  | 72 Sham |  | 71 Sham | Scl-Ab III | 25 mg/kg sc twice week |  | - | - | - | - |
|  |  |  |  |  | vehicle | - |  | - | - | - | - |
| Liu *et al.*  (2012) [66] | 36 | | 36 | | PE suspension + Scl-Ab III | 50𝜇L ia once week + 25 mg/kg sc twice week | titanium rods, dual acid-etched surface | - | - | - | - |
|  |  |  |  |  | PE suspension + antibody vehicle | 50𝜇L ia once week + vehicle  sc twice week |  | - | - | - | - |
|  |  |  |  |  | particle vehicle + antibody vehicle | - |  | - | - | - | - |
| Virdi *et al.*  (2012) [39] | 90 | | 88 | | Scl-Ab | 25mg/kg sc | cp-Ti, dual acid-etched surface | 4 & 8 weeks: significantly greater | No detectable differences | 8 weeks: greater | - |
|  |  |  |  |  | saline solution | - |  | - |  | - | - |
| Ominsky *et al.* (2011) [59] | 43 | | 29 | | Scl-Ab V | 30mg/kg sc every 2 weeks | stainless steel K-wire | FD: 56.0 ± 6.7 mm^2^ | - | - | - |
|  |  |  |  |  | vehicle | - |  | FD: 54.7 ± 2.0 mm^2^ | - | - | - |
| Agholme *et al.* (2010) [63] | 68 | | 64 | | Scl-Ab III | 25mg/kg sc twice week | stainless steel screws (mechanical tests); PMMA (𝜇CT) | - | - | - | - |
|  |  |  |  |  | saline solution | - |  | - | - | - | - |

Tt.Ar – Total cross-sectional Area/Subperiosteal Area; Ct.Ar – Cortical Area; M.Ar – Medullary Area; FD – Femoral Diaphysis.
