# Supplementary material for: Antisclerostin Effect on Osseointegration and Bone Remodeling
Source: J Clin Med. 2023 Feb 6;12(4):1294. doi: 10.3390/jcm12041294 (PMC9964545; doi:10.3390/jcm12041294)
Supplement: Supplementary file 1 [file jcm-12-01294-s001.zip › Suppl. Table 3.docx]

Table S3. Osseointegration/Bone formation parameters - Part III.

|  | Sample Size  (Initial) | | Sample Size  (Final) | | Drug/Control | Dosage & Administration Route | Implant | Bone Thickness | Tb.Th | | Tb.N | Tb.Sp | Ct.Th |
| --- | --- | --- | --- | --- | --- | --- | --- | --- | --- | --- | --- | --- | --- |
| Korn *et al.*  (2019) [61] | 128 | | 124 | | sclerostin antibody | 100mg/kg iv once week | reference-coated implant | - | µCT | higher than control group | - | - | - |
|  |  |  |  |  |  |  | ZOL-coated implant | - |  |  | - | - | - |
|  |  |  |  |  | non antibody applied | - | reference-coated implant | - | - | | - | - | - |
|  |  |  |  |  |  |  | ZOL-coated implant | - | - | | - | - | - |
| Yu *et al.*  (2018) [40] | 60 | | 60 | | Scl-Ab | 25mg/kg sc | cp-Ti, solid cylinder implants with titanium plasma-sprayed surface | - | - | | 8 weeks: greater than control group | - | - |
|  |  |  |  |  | PBS | - |  | - | - | | - | - | - |
| Virdi *et al.*  (2015) [35] | 144 | 72 OVX | 142 | 71 OVX | Scl-Ab III | 25 mg/kg sc twice week | cp-Ti, dual acid-etched surface | - | - | | - | - | increase over time |
|  |  |  |  |  | vehicle | - |  | - | - | | - | - | - |
|  |  | 72 Sham |  | 71 Sham | Scl-Ab III | 25 mg/kg sc twice week |  | - | increase | | little or no effect | - | increase over time, more significant than OVX group |
|  |  |  |  |  | vehicle | - |  | - | - | | - | - | - |
| Liu *et al.*  (2012) [66] | 36 | | 36 | | PE suspension + Scl-Ab III | 50𝜇L ia once week + 25 mg/kg sc twice week | titanium rods, dual acid-etched surface | - | 192 ± 26 𝜇m | | 2.01 ± 0.32 mm^-1^ | 502 ± 93 𝜇m | - |
|  |  |  |  |  | PE suspension + antibody vehicle | 50𝜇L ia once week + vehicle  sc twice week |  | - | 137 ± 19 𝜇m | | 0.92 ± 0.18 mm^-1^ | 1182 ± 216 𝜇m | - |
|  |  |  |  |  | particle vehicle + antibody vehicle | - |  | - | 142 ± 20 𝜇m | | 1.31 ± 0.34 mm^-1^ | 869 ± 216 𝜇m | - |
| Virdi *et al.*  (2012) [39] | 90 | | 88 | | Scl-Ab | 25mg/kg sc | cp-Ti, dual acid-etched surface | 8 weeks: greater than control group | - | | - | - | 8 weeks: greater |
|  |  |  |  |  | saline solution | - |  | - | - | | - | - | - |
| Ominsky *et al.* (2011) [59] | 43 | | 29 | | Scl-Ab V | 30mg/kg sc every 2 weeks | stainless steel K-wire | - | FN: 194 ± 6 𝜇m | | - | - | - |
|  |  |  |  |  | vehicle | - |  | - | FN: 152 ± 12 𝜇m | | - | - | - |
| Agholme *et al.* (2010) [63] | 68 | | 64 | | Scl-Ab III | 25mg/kg sc twice weeks | stainless steel screws (mechanical tests); PMMA (𝜇CT) | - | IT: 117 ± 5.7𝜇m  CT: 121 ± 3.8𝜇m | | IT: 1.9 ± 0.34 𝜇m^-1^  CT: 2.2 ± 0.36 𝜇m^-1^ | IT: 304 ± 54𝜇m  CT: 277 ± 56𝜇m | - |
|  |  |  |  |  | saline solution | - |  | - | IT: 92 ± 4.1𝜇m  CT: 93 ± 3.1𝜇m | | IT: 2.1 ± 0.48 𝜇m^-1^  CT: 2.4 ± 0.40 𝜇m^-1^ | IT: 273 ± 48𝜇m  CT: 244 ± 42𝜇m | - |

Tb.Th – Trabecular Thickness; Tb.N – Trabecular Number; Tb.Sp – Trabecular Separation; Ct.Th – Cortical Thickness; FN – Femoral Neck; IT – Implanted Tibia; CT – Contralateral Tibia.
