# Supplementary material for: Antisclerostin Effect on Osseointegration and Bone Remodeling
Source: J Clin Med. 2023 Feb 6;12(4):1294. doi: 10.3390/jcm12041294 (PMC9964545; doi:10.3390/jcm12041294)
Supplement: Supplementary file 1 [file jcm-12-01294-s001.zip › Suppl. Table 4.docx]

Table S4. Osseointegration/Bone formation parameters - Part IV.

|  | Sample Size  (Initial) | | Sample Size  (Final) | | Drug/Control | Dosage & Administration Route | Implant | SMI | MS/BS | MAR | BFR/BS | |
| --- | --- | --- | --- | --- | --- | --- | --- | --- | --- | --- | --- | --- |
| Korn *et al.*  (2019) [61] | 128 | | 124 | | sclerostin antibody | 100mg/kg iv once week | reference-coated implant | - | - | - | - | |
|  |  |  |  |  |  |  | ZOL-coated implant | - | - | - | - | |
|  |  |  |  |  | non-antibody applied | - | reference-coated implant | - | - | - | - | |
|  |  |  |  |  |  |  | ZOL-coated implant | - | - | - | - | |
| Yu *et al.*  (2018) [40] | 60 | | 60 | | Scl-Ab | 25mg/kg sc | cp-Ti, solid cylinder implants with titanium plasma-sprayed surface | - | - | - | - | |
|  |  |  |  |  | PBS | - |  | - | - | - | - | |
| Virdi *et al.*  (2015) [35] | 144 | 72 OVX | 142 | 71 OVX | Scl-Ab III | 25 mg/kg sc twice week | cp-Ti, dual acid-etched surface | - | - | - | 4 weeks: 4.6-fold increase vs control  increase attenuated overtime | |
|  |  |  |  |  | vehicle | - |  | - | - | - | - | |
|  |  | 72 Sham |  | 71 Sham | Scl-Ab III | 25 mg/kg sc twice week |  | - | - | - | 4 weeks: 7-fold increase vs control  increase attenuated overtime | |
|  |  |  |  |  | vehicle | - |  | - | - | - | - | |
| Liu *et al.*  (2012) [66] | 36 | | 36 | | PE suspension + Scl-Ab III | 50𝜇L ia once week + 25 mg/kg sc twice week | titanium rods, dual acid-etched surface | 1.09 ± 0.46 | 17.64 ± 3.25 % | 1.56 ± 0.26 𝜇m/day | 102.14 ± 34.47 𝜇m^3^/𝜇m^2^/day×100 | |
|  |  |  |  |  | PE suspension + antibody vehicle | 50𝜇L ia once week + vehicle sc twice week |  | 2.18 ± 0.60 | 9.83 ± 4.78 % | 0.77 ± 0.16 𝜇m/day | 29.69 ± 19.77 𝜇m^3^/𝜇m^2^/day×100 | |
|  |  |  |  |  | particle vehicle + antibody vehicle | - |  | 1.55 ± 0.43 | 12.04 ± 2.12 % | 1.11 ± 0.16 𝜇m/day | 49.53 ± 15.18 𝜇m^3^/𝜇m^2^/day×100 | |
| Virdi *et al.*  (2012) [39] | 90 | | 88 | | Scl-Ab | 25mg/kg sc | cp-Ti, dual acid-etched surface | decrease over time | - | - | - | |
|  |  |  |  |  | saline solution | - |  | - | - | - | - | |
| Ominsky *et al.* (2011) [59] | 43 | | 29 | | Scl-Ab V | 30mg/kg sc every 2 weeks | stainless steel K-wire | - | - | - | **2-3.5 wks** | FN: 157.6 ± 20.1 𝜇m^3^/𝜇m^2^/yr  Ps.FD: 187 ± 36 𝜇m^3^/𝜇m^2^/yr  Ec.FD: 238 ± 42 𝜇m^3^/𝜇m^2^/yr |
|  |  |  |  |  |  |  |  |  |  |  | **8-9.5 wks** | FN: 100.4 ± 17.9 𝜇m^3^/𝜇m^2^/yr  Ps.FD: 15.0 ± 5.2 𝜇m^3^/𝜇m^2^/yr  Ec.FD: 270 ± 37 𝜇m^3^/𝜇m^2^/yr |
|  |  |  |  |  | vehicle | - |  | - | - | - | **2-3.5 wks** | FN: 44.8 ± 8.0 𝜇m^3^/𝜇m^2^/yr  Ps.FD: 79.3 ± 15.8 𝜇m^3^/𝜇m^2^/yr  Ec.FD: 50.5 ± 14.8 𝜇m^3^/𝜇m^2^/yr |
|  |  |  |  |  |  |  |  |  |  |  | **8-9.5 wks** | FN: 62.4 ± 12.1 𝜇m^3^/𝜇m^2^/yr  Ps.FD: 6.4 ± 3.4 𝜇m^3^/𝜇m^2^/yr  Ec.FD: 35.5 ± 10.6 𝜇m^3^/𝜇m^2^/yr |
| Agholme *et al.* (2010) [63] | 68 | | 64 | | Scl-Ab III | 25mg/kg sc twice weeks | stainless steel screws (mechanical tests); PMMA (𝜇CT) | - | - | - | - | |
|  |  |  |  |  | saline solution | - |  | - | - | - | - | |

SMI – Structural Model Index; MS/BS – Mineralizing Surface; MAR – Mineral Apposition Rate; BFR/BS – Bone Formation Rate; FN – Femoral Neck; FD – Femoral Diaphysis; Ps – Periosteal; Ec – Endocortical.
