# Supplementary material for: Antisclerostin Effect on Osseointegration and Bone Remodeling
Source: J Clin Med. 2023 Feb 6;12(4):1294. doi: 10.3390/jcm12041294 (PMC9964545; doi:10.3390/jcm12041294)
Supplement: Supplementary file 1 [file jcm-12-01294-s001.zip › Suppl. Table 5.docx]

Table S5. Osseointegration/Bone formation parameters - Part V.

|  | Sample Size  (Initial) | | Sample Size  (Final) | | Drug/Control | Dosage & Administration Route | Implant | ES/BS | Oc.S/BS | Cortical Porosity |
| --- | --- | --- | --- | --- | --- | --- | --- | --- | --- | --- |
| Korn *et al.*  (2019) [61] | 128 | | 124 | | sclerostin antibody | 100mg/kg iv once week | reference-coated implant | - | - | - |
|  |  |  |  |  |  |  |  |  |  |  |
|  |  |  |  |  |  |  | ZOL-coated implant | - | - | - |
|  |  |  |  |  |  |  |  |  |  |  |
|  |  |  |  |  | non antibody applied | - | reference-coated implant | - | - | - |
|  |  |  |  |  |  |  | ZOL-coated implant | - | - | - |
| Yu *et al.*  (2018) [40] | 60 | | 60 | | Scl-Ab | 25mg/kg sc | cp-Ti, solid cylinder implants with titanium plasma-sprayed surface | - | - | - |
|  |  |  |  |  | PBS | - |  | - | - | - |
| Virdi *et al.*  (2015) [35] | 144 | 72 OVX | 142 | 71 OVX | Scl-Ab III | 25 mg/kg sc twice week | cp-Ti, dual acid-etched surface | decrease greater than 50% | - | - |
|  |  |  |  |  | vehicle | - |  | - | - | - |
|  |  | 72 Sham |  | 71 Sham | Scl-Ab III | 25 mg/kg sc twice week |  | decrease greater than 50% | - | - |
|  |  |  |  |  | vehicle | - |  | - | - | - |
| Liu *et al.*  (2012) [66] | 36 | | 36 | | PE suspension + Scl-Ab III | 50𝜇L ia once week + 25 mg/kg sc twice week | titanium rods, dual acid-etched surface | 17.10 ± 3.17 % | - | - |
|  |  |  |  |  | PE suspension + antibody vehicle | 50𝜇L ia once week + vehicle sc twice week |  | 10.83 ± 1.92 % | - | - |
|  |  |  |  |  | particle vehicle + antibody vehicle | - |  | 10.26 ± 2.71 % | - | - |
| Virdi *et al.*  (2012) [39] | 90 | | 88 | | Scl-Ab | 25mg/kg sc | cp-Ti, dual acid-etched surface | - | - | - |
|  |  |  |  |  | saline solution | - |  | - | - | - |
| Ominsky *et al.* (2011) [59] | 43 | | 29 | | Scl-Ab V | 30mg/kg sc every 2 weeks | stainless steel K-wire | FN: 0.86 ± 0.19 % | FN: 0.26 ± 0.09 % | FD: 0.99 ± 0.07 % |
|  |  |  |  |  | vehicle | - |  | FN: 1.95 ± 0.33 % | FN: 0.33 ± 0.08 % | FD: 1.13 ± 0.10 % |
| Agholme *et al.* (2010) [63] | 68 | | 64 | | Scl-Ab III | 25mg/kg sc twice weeks | stainless steel screws (mechanical tests); PMMA (𝜇CT) | - | - | - |
|  |  |  |  |  | saline solution | - |  | - | - | - |

ES/BS – Eroded Surface; Oc.S/BS – Osteoclast Surface; FN – Femoral Neck; FD - Femoral Diaphysis.
