# Supplementary material for: Antisclerostin Effect on Osseointegration and Bone Remodeling
Source: J Clin Med. 2023 Feb 6;12(4):1294. doi: 10.3390/jcm12041294 (PMC9964545; doi:10.3390/jcm12041294)
Supplement: Supplementary file 1 [file jcm-12-01294-s001.zip › Suppl. Table 6.docx]

Table S6. Implant fixation properties.

|  | Sample Size  (Initial) | | Sample Size  (Final) | | Drug/Control | Dosage & Administration Route | Implant | Fixation Strength | | Stiffness | | Energy | |
| --- | --- | --- | --- | --- | --- | --- | --- | --- | --- | --- | --- | --- | --- |
| Korn *et al.*  (2019) [61] | 128 | | 124 | | sclerostin antibody | 100mg/kg iv once week | reference-coated implant | - | | - | | - | |
|  |  |  |  |  |  |  | ZOL-coated implant | - | | - | | - | |
|  |  |  |  |  | non antibody applied | - | reference-coated implant | - | | - | | - | |
|  |  |  |  |  |  |  | ZOL-coated implant | - | | - | | - | |
| Yu *et al.*  (2018) [40] | 60 | | 60 | | Scl-Ab | 25mg/kg sc | cp-Ti, solid cylinder with Ti plasma-sprayed surface implant | - | | - | | - | |
|  |  |  |  |  | PBS | - |  | - | | - | | - | |
| Virdi *et al.*  (2015) [35] | 144 | 72 OVX | 142 | 71 OVX | Scl-Ab III | 25 mg/kg sc twice week | cp-Ti, dual acid-etched surface | increase over time | | - | | - | |
|  |  |  |  |  | vehicle | - |  | - | | - | | - | |
|  |  | 72 Sham |  | 71 Sham | Scl-Ab III | 25 mg/kg sc twice week |  | increase over time  greater than OVX group  2x higher than control group | | sig. increase over time, with better results than OVX | | greater than OVX group  8 & 12 wks: significant increase | |
|  |  |  |  |  | vehicle | - |  | - | | - | | - | |
| Liu *et al.*  (2012) [66] | 36 | | 36 | | PE suspension + Scl-Ab III | 50𝜇L ia once week + 25 mg/kg sc twice week | titanium rods, dual acid-etched surface | 2.00 ± 0.29 N/mm^2^ | | 186 ± 114 N/mm | | 348 ± 156 Nmm | |
|  |  |  |  |  | PE suspension + antibody vehicle | 50𝜇L ia once week + vehicle sc twice week |  | 0.79 ± 0.40 N/mm^2^ | | 127 ± 89 N/mm | | 104 ± 67 Nmm | |
|  |  |  |  |  | particle vehicle + antibody vehicle | - |  | 1.32 ± 0.45 N/mm^2^ | | 221 ± 127 N/mm | | 154 ± 81 Nmm | |
| Virdi *et al.*  (2012) [39] | 90 | | 88 | | Scl-Ab | 25mg/kg sc | cp-Ti, dual acid-etched surface | 4 wks: 1,9 times higher  8 wks: 2,2 times higher | | sig. increase over time, but not overall group effect.  8 wks: drug effect apparent | | 4 & 8 wks: sig. increase with similar pattern as fixation strength | |
|  |  |  |  |  |  |  |  | **Univariate correlation with** | | **Univariate correlation with** | | **Univariate correlation with** | |
|  |  |  |  |  |  |  |  | BV/TV: 0.596  SMI: -0.678  Tb.Th: 0.719  Tb.Sp: 0.078  Tb.N: -0.121 | Ct.Ar: 0.671  Ct.Th: 0.666  Tt.Ar: 0.502  M.Ar: -0.255 | BV/TV: 0.436  SMI: -0.519  Tb.Th: 0.517  Tb.Sp: 0.053  Tb.N: -0.120 | Ct.Ar: 0.428  Ct.Th: 0.485  Tt.Ar: 0.205  M.Ar: -0.358 | BV/TV: 0.577  SMI: -0.662  Tb.Th: 0.717  Tb.Sp: 0.069  Tb.N: -0.094 | Ct.Ar: 0.636  Ct.Th: 0.595  Tt.Ar: 0.538  M.Ar: -0.135 |
|  |  |  |  |  | saline solution | - |  | **Univariate correlation with** | | **Univariate correlation with** | | **Univariate correlation with** | |
|  |  |  |  |  |  |  |  | BV/TV: -0.016  SMI: -0.187  Tb.Th: 0.019  Tb.Sp: 0.124  Tb.N: -0.148 | Ct.Ar: 0.052  Ct.Th: 0.111  Tt.Ar: -0.193  M.Ar: -0.260 | BV/TV: 0.115  SMI: -0.540  Tb.Th: 0.017  Tb.Sp: 0.062  Tb.N: -0.031 | Ct.Ar: -0.222  Ct.Th: -0.094  Tt.Ar: -0.266  M.Ar: -0.255 | BV/TV: -0.015  SMI: -0.027  Tb.Th: 0.082  Tb.Sp: 0.065  Tb.N: -0.129 | Ct.Ar: 0.047  Ct.Th: 0.180  Tt.Ar: -0.102  M.Ar: -0.204 |
| Ominsky *et al.* (2011) [59] | 43 | | 29 | | Scl-Ab V | 30mg/kg sc every 2 weeks | stainless steel K-wire | - | | increase of 48% in torsional stiffness | | - | |
|  |  |  |  |  | vehicle | - |  | - | | - | | - | |
| Agholme *et al.* (2010) [63] | 68 | | 64 | | Scl-Ab III | 25mg/kg sc twice week | stainless steel screws (mechanical tests); PMMA (𝜇CT) | - | | - | | - | |
|  |  |  |  |  | saline solution | - |  | - | | - | | - | |

BV/TV – Bone Volume per Total Volume; SMI – Structural Model Index; Tb.Th – Trabecular Thickness; Tb.Sp – Trabecular Separation; Tb.N. – Trabecular Number; Ct.Ar – Cortical Area; Ct.Th – Cortical Thickness; Tt.Ar - Total cross-sectional Area/Subperiosteal Area; M.Ar – Medullary Area.
