# Supplementary material for: Antisclerostin Effect on Osseointegration and Bone Remodeling
Source: J Clin Med. 2023 Feb 6;12(4):1294. doi: 10.3390/jcm12041294 (PMC9964545; doi:10.3390/jcm12041294)
Supplement: Supplementary file 1 [file jcm-12-01294-s001.zip › Suppl. Table 7.docx]

Table S7. Bone remodeling/formation parameters – Part I.

|  | Sample Size  (Initial) | | Sample Size  (Final) | | Drug/Control | Dosage &  Administration Route | BMD | | | | | | BMC | | | BA/TA | BV/TV | | | | |
| --- | --- | --- | --- | --- | --- | --- | --- | --- | --- | --- | --- | --- | --- | --- | --- | --- | --- | --- | --- | --- | --- |
| Liu *et al.*  (2018) [57] | 50 | 40 OVX | 50 | 40 OVX | Scl-Ab VI | 18.2mg/kg sc twice week | - | | | | | | Vertebral & Leg: increase vs control group | | | - | levels restored and exceeded both control groups | | | | |
|  |  |  |  |  | Scl-Ab VI + DAB | 18.1mg/kg sc + 18.1mg/kg sc twice week | - | | | | | | Vertebral & Leg: sig. increase vs control and Scl-Ab groups | | | - |  |  |  |  |  |
|  |  |  |  |  | saline vehicle | - | - | | | | | | - | | | - | - | | | | |
|  |  | 10 Sham |  | 10 Sham | saline vehicle | - | - | | | | | | - | | | - | - | | | | |
|  | 45 | | 45 | | Scl-Ab VI | 25mg/kg sc twice week | - | | | | | | - | | | - | **15 weeks** | higher in loaded and underloaded sites vs control  no sig. differences between loaded & underloaded sites | | | |
|  |  |  |  |  | Scl-Ab VI + DAB | 25mg/kg sc + 25mg/kg sc twice week | - | | | | | | - | | | - |  |  |  |  |  |
|  |  |  |  |  | saline vehicle | - | - | | | | | | - | | | - | 13.9% lower in underloaded mandible vs loaded mandible | | | | |
| Wu *et al.*  (2018) [60] | 50 | 5 Sham | 50 | 5 Sham | **Baseline** | | TM: 231 ± 30.14 mg/cm^3^ | | | | | | - | | | - | - | | | | |
|  |  | 5 OVX |  | 5 OVX | **Baseline** | | TM: 165 ± 27.65 mg/cm^3^ | | | | | | - | | | - | - | | | | |
|  |  | 40 OVX |  | 40 OVX | Scl-Ab | 25mg/kg sc twice week | 1.24x higher increase vs control | | | | | | - | | | - | 1.75x higher increase vs control | | | | |
|  |  |  |  |  | PTH 1-34 | 60𝜇g/kg sc thrice week | 1.25x higher increase vs control | | | | | | - | | | - | 1.77x higher increase vs control | | | | |
|  |  |  |  |  | Scl-Ab +  PTH 1-34 | 25mg/kg sc twice week + 60𝜇g/kg sc thrice week | 1.35x higher increase vs control | | | | | | - | | | - | 2.31x higher increase vs control | | | | |
|  |  |  |  |  | vehicle | - | 12 weeks: sig. decrease | | | | | | - | | | - | - | | | | |
| Taut *et al.*  (2013) [65] | 69 | | 69 | | EP: Scl-Ab III | 25 mg/kg sc twice week | **3 wks** | | | higher increase vs vehicle | | | - | | | - | **3 wks** | | higher than vehicle group | |  |
|  |  |  |  |  |  |  | **6 wks** | | | sig. higher increase vs veh  no differences vs healthy | | |  |  |  |  | **6 wks** | | sig. higher vs vehicle and no sig. difference vs healthy | |  |
|  |  |  |  |  |  | 15 𝜇L of 35.6mg/mL solution locally twice week | 3 & 6 weeks: limited increase | | | | | | - | | | - | limited increase | | | | |
|  |  |  |  |  |  |  | **3 wks** | | lower than vehicle | | | |  |  |  |  | **3 wks** | | lower increase than veh | |  |
|  |  |  |  |  |  |  | **6 wks** | | little higher than vehicle | | | |  |  |  |  | **6 wks** | | little higher increase vs veh | |  |
|  |  |  |  |  | EP: vehicle | - | 3 wks: increase, stabilizing at 6 wks | | | | | | - | | | - | 3 wks: increased, stabilizing 6 wks | | | | |
|  |  |  |  |  | healthy: PBS | - | 6 weeks: sig. greater vs veh | | | | | | - | | | - | significantly higher vs vehi | | | | |
| Virk *et al.*  (2013) [58] | 72 | | 72 | | Scl-Ab III | 25mg/kg sc twice week | - | | | | | | - | | | 12 weeks: 44.4 ± 9.1 %  2 weeks: 33.5 ± 13.5 %  2-4 weeks: 40.4 ± 15 % | 12 weeks: 60 ± 17 %  2 weeks: 44.4 ± 20 %  2-4 weeks: 49.6 ± 20.4 % | | | | |
|  |  |  |  |  | PBS | - | - | | | | | | - | | | 37.3 ± 10.2 % | 39.3 ± 15.3 % | | | | |
|  | 30 | | 30 | | Scl-Ab III | 25mg/kg | - | | | | | | - | | | 28.2 ± 10.9 % | 37.4 ± 0.1 % | | | | |
|  |  |  |  |  | PBS | - | - | | | | | | - | | | 15.2 ± 9.1 % | 19.2 ± 9.5 % | | | | |
| McDonald *et al.* (2012) [33] | 132 | 66 Sham | 127 | | Scl-Ab III | 25mg/kg sc twice week | - | | | | | | - | | | - | 1 week: 8.5 ± 6.3 %  2 weeks: 32.0 ± 7.9 %  3 weeks: 35.8 ± 7.9 % | | | | |
|  |  |  |  |  | saline solution | - | - | | | | | | - | | | - | 1 week: 8.3 ± 4.9 %  2 weeks: 28.0 ± 5.5 %  3 weeks: 28.9 ± 11.0 % | | | | |
|  |  | 66 OVX |  |  | Scl-Ab III | 25mg/kg sc twice week | - | | | | | | - | | | - | 1 week: 18.6 ± 8.6 %  2 weeks: 25.3 ± 7.4 %  3 weeks: 18.9 ± 9.2 % | | | | |
|  |  |  |  |  | saline solution | - | - | | | | | | - | | | - | 1 week: 21.3 ± 14.3 %  2 weeks: 15.5 ± 5.2 %  3 weeks: 13.7 ± 3.4 % | | | | |
| Ominsky *et al.*  (2011) [59] | 35 | | 32 | | Scl-Ab III | 25mg/kg sc twice week | 11 % increase compared to vehicle, in fractured femur | | | | | | **Fractured Femur:** 19% increase compared to vehicle | | | - | **Fractured Femur:** 41 % greater compared to vehicle | | | | |
|  |  |  |  |  |  |  |  |  |  |  |  |  | **Intact Femur:**  FN: 3.68 ± 0.14 mg/mm  Tb.DF: 4.26 ± 0.31 mg/mm  Ct.FD: 10.05 ± 0.29 mg/mm | | |  | **Intact Femur:**  Tb.DF: 23.1 ± 2.0 % | | | | |
|  |  |  |  |  | vehicle | - | - | | | | | | **Intact Femur:**  FN: 3.23 ± 0.08 mg/mm  Tb.DF: 3.07 ± 0.15 mg/mm  Ct.FD: 9.10 ± 0.23 mg/mm | | | - | **Intact Femur:**  Tb.DF: 16.4 ± 3.8 % | | | | |
| Tian *et al*.  (2011) [34] | 67 | | 67 | | - | - | - | | | | | | - | | | - | **Baseline** | | | PTM: 14.3 ± 4.8 % | |
|  |  |  |  |  | Scl-Ab III | 5mg/kg sc twice week | - | | | | | | - | | | - | NL.PTM: 24.6 ± 9.2 %  UL.PTM: 19.4 ± 3.3 % | | | | |
|  |  |  |  |  |  | 25mg/kg sc twice week | - | | | | | | - | | | - | NL.PTM: 34.7 ± 6.5 %  UL.PTM: 21.2 ± 6.3 % | | | | |
|  |  |  |  |  | saline solution | - | - | | | | | | - | | | - | NL.PTM: 13.9 ± 2.7 %  UL.PTM: 13.4 ± 1.1 % | | | | |
| Li *et al.*  (2010) [38] | 28 | | 26 | | Scl-Ab III | 25mg/kg sc twice week | LV: 678 ± 16 mg/mL  FD: 1360 ± 8 mg/mL  FN: 1090 ± 31 mg/mL  Tb.LV: 561 ± 22 mg/mL  Tb.DF: 428.1 ± 21.9 mg/mL  Ct.LV: 674 ± 10 mg/mL | | | | | | LV: 9.07 ± 0.26 mg/mm  FD: 15.87 ± 1.22 mg/mm  FN: 5.88 ± 0.33 mg/mm  Tb.LV: 4.02 ± 0.16 mg/mm  Tb.DF: 5.63 ± 0.36 mg/mL  Ct.LV: 5.1 ± 0.1 mg/mm | | | - | Tb.LV: 43.9 ± 2.8 %  Tb.DF: 24.5 ± 2.7 %  FN: 88.5 ± 3.5 %  PT: 19.0 ± 2.1 % | | | | |
|  |  |  |  |  |  | 5mg/kg sc twice week | LV: 626 ± 21 mg/mL  FD: 1363 ± 9 mg/mL  FN: 1064 ± 12 mg/mL  Tb.LV: 499 ± 26 mg/mL  Tb.DF: 411.5 ± 22.3 mg/mL  Ct.LV: 633 ± 16 mg/mL | | | | | | LV: 8.15 ± 0.22 mg/mm  FD: 15.85 ± 0.63 mg/mm  FN: 4.90 ± 0.32 mg/mm  Tb.LV: 3.55 ± 0.13 mg/mm  Tb.DF: 4.89 ± 0.35 mg/mL  Ct.LV: 4.6 ± 0.1 mg/mm | | | - | Tb.LV: 36.1 ± 3.1 %  Tb.DF: 22.5 ± 2.8 %  FN: 87.6 ± 2.0 %  PT: 17.0 ± 3.3 % | | | | |
|  |  |  |  |  | vehicle | - | LV: 500 ± 14 mg/mL  FD: 1343 ± 10 mg/mL  FN: 975 ± 21 mg/mL  Tb.LV: 375 ± 16 mg/mL  Tb.DF: 290.2 ± 10.8 mg/mL  Ct.LV: 508 ± 13 mg/mL | | | | | | LV: 6.22 ± 0.24 mg/mm  FD: 13.00 ± 0.48 mg/mm  FN: 4.83 ± 0.23 mg/mm  Tb.LV: 2.81 ± 0.13 mg/mm  Tb.DF: 3.79 ± 0.22 mg/mL  Ct.LV: 3.4 ± 0.1 mg/mm | | | - | Tb.LV: 22.4 ± 1.6 %  Tb.DF: 9.5 ± 1.3 %  FN: 74.8 ± 2.3 %  PT: 7.1 ± 0.8 % | | | | |
| Ominsky *et al.*  (2010) [64] | 12 | | 12 | | Scl-Ab IV | 3mg/kg sc once month | **%Change** | **DXA** | | | WB: 4.4 ±5.4 %  LS: 9.8 ± 1.4 %  FN: 10.2 ± 10.9 %  UDR: 8.5 ± 0.9 % | | **%Change** | **DXA** | WB: 5.8 ± 6.2 %  LS: 15.0 ± 0.3 %  FN: 17.3 ± 11.6 %  UDR: 9.7 ± 5.5 % | - | - | | | | |
|  |  |  |  |  |  |  |  | **pQCT** | | | DRM: 1.8 ± 2.4 %  PTM: 3.2 ± 4.5 %  Tb.DRM: 13.1 ± 26.0 %  Tb.PTM: 8.4 ± 18.7 %  Ct.DRD: 0.2 ± 1.2 %  Ct.PTD: -1.2 ± 3.3 % | |  | **pQCT** | DRM: 4.1 ± 5.1 %  PTM: 9.4 ± 15.0 %  Ct.DRD: 7.3 ± 7.2 %  Ct.PTD: 9.7 ± 9.6 % |  |  | | | | |
|  |  |  |  |  |  | 10mg/kg sc once month | **%Change** | **DXA** | | | WB: 10.8 ± 3.2 %  LS: 4.2 ± 3.8 %  FN: 11.5 ± 5.8 %  UDR: 6.2 ± 5.6 % | | **%Change** | **DXA** | WB: 19.2 ± 6.7 %  LS: 8.1 ± 6.6 %  FN: 10.5 ± 2.8 %  UDR: 11.3 ± 8.3 % | - | - | | | | |
|  |  |  |  |  |  |  |  | **pQCT** | | | DRM: 8.5 ± 2.2 %  PTM: 10.9 ± 3.7 %  Tb.DRM: 21.7 ± 6.8 %  Tb.PTM: 21.1 ± 6.7 %  Ct.DRD: -1.6 ± 1.4 %  Ct.PTD: -1.1 ± 0.9 % | |  | **pQCT** | DRM: 7.7 ± 1.7 %  PTM: 17.4 ± 5.9 %  Ct.DRD: 3.3 ± 1.9 %  Ct.PTD: 11.0 ± 3.8 % |  |  |  |  |  |  |
|  |  |  |  |  |  | 30mg/kg sc once month | **%Change** | **DXA** | | | WB: 9.4 ± 2.8 %  LS: 11.1 ± 3.0 %  FN: 19.5 ± 3.4 %  UDR: 15.1 ± 1.0 % | | **%Change** | **DXA** | WB: 24.0 ± 2.2 %  LS: 16.5 ± 6.2 %  FN: 35.2 ± 7.2 %  UDR: 19.8 ± 4.4 % | - | - | | | | |
|  |  |  |  |  |  |  |  | **pQCT** | | | DRM: 14.2 ± 3.4 %  PTM: 18.8 ± 4.7 %  Tb.DRM: 34.3 ± 14.4 %  Tb.PTM: 34.9 ± 8.2 %  Ct.DRD: -0.9 ± 1.6 %  Ct.PTM: 1.0 ± 1.0 % | |  | **pQCT** | DRM: 19.8 ± 7.2 %  PTM: 27.3 ± 6.2 %  Ct.DRD: 8.8 ± 2.5 %  Ct.PTM: 13.6 ± 2.8 % |  |  |  |  |  |  |
|  |  |  |  |  | vehicle | - | **%Change** | **DXA** | | | WB: 1.6 ± 3.0 %  LS: 1.7 ± 1.8 %  FN: 4.6 ± 1.8 %  UDR: 2.7 ± 4.0 % | | **%Change** | **DXA** | WB: 6.4 ± 3.0 %  LS: 2.8 ± 2.7 %  FN: 5.4 ± 5.1 %  UDR: 5.3 ± 3.7 % | - | - | | | | |
|  |  |  |  |  |  |  |  | **pQCT** | | | DRM: 2.6 ± 2.0 %  PTM: 2.9 ± 3.5 %  Tb.DRM: -3.2 ± 4.0 %  Tb.PTM: -1.7 ± 4.9 %  Ct.DRD: -0.3 ± 0.5 %  Ct.PTD: 1.3 ± 0.7 % | |  | **pQCT** | DRM: 0.8 ± 1.6 %  PTM: -1.0 ± 3.2 %  Ct.DRD: 2.4 ± 0.7 %  Ct.PTD: 2.5 ± 3.2 % |  |  |  |  |  |  |
| Tian *et al.*  (2010) [62] | 32 | | 32 | | - | - | - | | | | | | - | | | - | **Baseline** | | | CVB: 25.7 ± 4.1 %  LVB: 25.1 ± 4.1 % | |
|  |  |  |  |  | Scl-Ab III | 5mg/kg sc twice week | - | | | | | | - | | | - | CVB: 29.4 ± 4.1 %  LVB: 31.9 ± 7.9 % | | | | |
|  |  |  |  |  |  | 25mg/kg sc twice week | - | | | | | | - | | | - | CVB: 37.5 ± 6.5 %  LVB: 45.2 ± 4.6 % | | | | |
|  |  |  |  |  | saline solution | - | - | | | | | | - | | | - | CVB: 23.8 ± 3.5 %  LVB: 24.4 ± 3.7 % | | | | |
| Saag *et al.*  (2017) [67] | 4093 | | 3150 | | Romosozumab → Alendronate | 210mg sc once month → 70mg po once week | **T-score**  **Baseline** | | | | | LS: -2.94 ± 1.25  TH: -2.78 ± 0.68  FN: -2.89 ± 0.49 | - | | | - | - | | | | |
|  |  |  |  |  |  |  | **%Change** | **12 mo** | | | | LS: 13.7%; TH: 6.2%; FN: 4.9% |  |  |  |  |  |  |  |  |  |
|  |  |  |  |  |  |  |  | **24 mo** | | | | LS: 15.2%; TH: 7.1%; FN: 5.9% |  |  |  |  |  |  |  |  |  |
|  |  |  |  |  |  |  |  | **36 mo** | | | | LS: 14.9%; TH: 7.0%; FN: 5.9% |  |  |  |  |  |  |  |  |  |
|  |  |  |  |  | Alendronate → Alendronate | 70mg po once week → 70mg po once week | **T-score**  **Baseline** | | | | | LS: -2.99 ± 1.24  TH: -2.18 ± 0.67  FN: -2.90 ± 0.50 | - | | | - | - | | | | |
|  |  |  |  |  |  |  | **%Change** | **12 mo** | | | | LS: 5.0%; TH: 2.8%; FN: 1.7% |  |  |  |  |  |  |  |  |  |
|  |  |  |  |  |  |  |  | **24 mo** | | | | LS: 7.1%; TH: 3.4%; FN: 3.6% |  |  |  |  |  |  |  |  |  |
|  |  |  |  |  |  |  |  | **36 mo** | | | | LS: 8.5%; TH: 3.6%; FN: 2.7% |  |  |  |  |  |  |  |  |  |
| McClung *et al.*  (2014) [41] | 419 | | 383 | | Romosozumab | 140mg sc every 3 months | **T-score**  **Baseline** | | | | | LS: -2.44 ± 0.70  TH: -1.58 ± 0.51  FN: -2.00 ± 0.54  DR: -2.24 ± 1.06 | - | | | - | - | | | | |
|  |  |  |  |  |  |  | **%Change** | **3 mo** | | | | LS: 2.4%; TH: 0.3%; FN: 0.4% |  |  |  |  |  |  |  |  |  |
|  |  |  |  |  |  |  |  | **6 mo** | | | | LS: 4.2%; TH: 0.9%; FN: 0.4% |  |  |  |  |  |  |  |  |  |
|  |  |  |  |  |  |  |  | **12 mo** | | | | LS: 5.4%; TH: 1.3%; FN: 1.8%; DR: -1.1% |  |  |  |  |  |  |  |  |  |
|  |  |  |  |  |  | 210mg sc every 3 months | **T-score**  **Baseline** | | | | | LS: -2.21 ± 0.69  TH: 1.65 ± 0.63  FN: -2.02 ± 0.57  DR: -1.98 ± 1.04 | - | | | - | - | | | | |
|  |  |  |  |  |  |  | **%Change** | **3 mo** | | | | LS: 3.1%; TH: 0.8%; FN: 0.9 % |  | | |  |  | | | |  |
|  |  |  |  |  |  |  |  | **6 mo** | | | | LS: 4.4%; TH: 1.1%; FN: 0.9% |  |  |  |  |  |  |  |  |  |
|  |  |  |  |  |  |  |  | **12 mo** | | | | LS: 5.5%; TH: 1.9%; FN: 1.4%; DR: -0.4 % |  |  |  |  |  |  |  |  |  |
|  |  |  |  |  |  | 70mg sc once month | **T-score**  **Baseline** | | | | | LS: -2.35 ± 0.79  TH: 1.69 ± 0.67  FN: -2.06 ± 0.55  DR: -1.78 ± 1.14 | - | | | - | - | | | |  |
|  |  |  |  |  |  |  | **%Change** | **3 mo** | | | | LS: 1.9%; TH: 0.4%; FN: -0.4% |  |  |  |  |  |  |  |  |  |
|  |  |  |  |  |  |  |  | **6 mo** | | | | LS: 4.1%; TH: 0.5%; FN: 0.2% |  |  |  |  |  |  |  |  |  |
|  |  |  |  |  |  |  |  | **12 mo** | | | | LS: 5.4%; TH: 1.3%; FN: 0.6%; DR: -1.8% |  |  |  |  |  |  |  |  |  |
|  |  |  |  |  |  | 140mg sc once month | **T-score**  **Baseline** | | | | | LS: -2.27 ± 0.77  TH: -1.67 ± 0.65  FN: -2.03 ± 0.58  DR: -2.11 ± 1.12 | - | | | - | - | | | |  |
|  |  |  |  |  |  |  | **%Change** | **3 mo** | | | | LS: 4.5%; TH: 1.0%; FN: 1.3% |  |  |  |  |  |  |  |  |  |
|  |  |  |  |  |  |  |  | **6 mo** | | | | LS: 7.1%; TH: 2.2%; FN: 2.1% |  |  |  |  |  |  |  |  |  |
|  |  |  |  |  |  |  |  | **12 mo** | | | | LS: 9.1%; TH: 3.4%; FN: 4.2%; DR: -1.0% |  |  |  |  |  |  |  |  |  |
|  |  |  |  |  |  | 210mg sc once month | **T-score**  **Baseline** | | | | | LS: -2.33 ± 0.57  TH: -1.45 ± 0.65  FN: -1.87 ± 0.58  DR: -2.03 ± 0.99 | - | | | - | - | | | |  |
|  |  |  |  |  |  |  | **%Change** | **3 mo** | | | | LS: 4.5%; TH: 1.1%; FN: 0.8% |  |  |  |  |  |  |  |  |  |
|  |  |  |  |  |  |  |  | **6 mo** | | | | LS: 8.2%; TH: 2.9%; FN: 1.9% |  |  |  |  |  |  |  |  |  |
|  |  |  |  |  |  |  |  | **12 mo** | | | | LS: 11.3%; TH: 4.1%; FN: 3.7%; DR: -1.2% |  |  |  |  |  |  |  |  |  |
|  |  |  |  |  | Alendronate | 70 mg po once week | **T-score**  **Baseline** | | | | | LS: -2.08 ± 0.69  TH: -1.55 ± 0.68  FN: -1.91 ± 0.61  DR: -2.08 ± 0.99 | - | | | - | - | | | |  |
|  |  |  |  |  |  |  | **%Change** | **3 mo** | | | | LS: 1.8%; TH: 0.6%; FN: 0.4% |  |  |  |  |  |  |  |  |  |
|  |  |  |  |  |  |  |  | **6 mo** | | | | LS: 2.6%; TH: 0.9%; FN: 0.5% |  |  |  |  |  |  |  |  |  |
|  |  |  |  |  |  |  |  | **12 mo** | | | | LS: 4.1%; TH: 1.9%; FN: 1.2%; DR: -0.3% |  |  |  |  |  |  |  |  |  |
|  |  |  |  |  | Teriparatide | 20𝜇g sc once day | **T-score**  **Baseline** | | | | | LS: -2.29 ± 0.57  TH: -1.32 ± 0.78  FN: -1.79 ± 0.67  DR: -2.05 ± 1.21 | - | | | - | - | | | |  |
|  |  |  |  |  |  |  | **%Change** | **3 mo** | | | | LS: 2.8%; TH: 0.7%; FN: 1.1% |  |  |  |  |  |  |  |  |  |
|  |  |  |  |  |  |  |  | **6 mo** | | | | LS: 4.8%; TH: 0.5%; FN: 0.5% |  |  |  |  |  |  |  |  |  |
|  |  |  |  |  |  |  |  | **12 mo** | | | | LS: 7.1%; TH: 1.3%; FN: 1.1%; DR: -1.7% |  |  |  |  |  |  |  |  |  |
|  |  |  |  |  | placebo | - | **T-score**  **Baseline** | | | | | LS: -2.29 ± 0.66  TH: -1.35 ± 0.65  FN: -1.76 ± 0.56  DR: -1.85 ± 1.04 | - | | | - | - | | | |  |
|  |  |  |  |  |  |  | **%Change** | **3 mo** | | | | LS: 0.5%; TH: -0.4%; FN: -0.2% |  |  |  |  |  |  |  |  |  |
|  |  |  |  |  |  |  |  | **6 mo** | | | | LS: 0.3%; TH: -0.6%; FN: -0.4% |  |  |  |  |  |  |  |  |  |
|  |  |  |  |  |  |  |  | **12 mo** | | | | LS: -0.1%; TH: -0.7%; FN: -1.1%; DR: -0.9% |  |  |  |  |  |  |  |  |  |
| Padhi *et al.*  (2014) [43] | 48 | 32 women | 46 | 31 women | Romosozumab | 1mg/kg sc every 2 weeks | **T-score**  **Baseline** | | | | | LS: -1.22 ± 0.93  TH: -0.88 ± 0.67  FN: -1.33 ± 0.41  DR: -0.93 ± 0.62 | - | | | - | - | | | |  |
|  |  |  |  |  |  | 2mg/kg sc every 4 weeks | **T-score**  **Baseline** | | | | | LS: -1.24 ± 0.46  TH: -0.90 ± 0.68  FN: -1.72 ± 0.37  DR: -0.55 ± 1.25 | - | | | - | - | | | |  |
|  |  |  |  |  |  | 2mg/kg sc every 2 weeks | **T-score**  **Baseline** | | | | | LS: -1.27 ± 0.29  TH: -1.17 ± 0.56  FN: -1.58 ± 0.64  DR: -1.37 ± 1.43 | - | | | - | - | | | |  |
|  |  |  |  |  |  | 3mg/kg sc every 4 weeks | **T-score**  **Baseline** | | | | | LS: -1.58 ± 0.47  TH: -0.72 ± 0.54  FN: -1.10 ± 0.64  DR: -0.83 ± 0.48 | - | | | - | - | | | |  |
|  |  |  |  |  | placebo | - | **T-score**  **Baseline** | | | | | LS: -1.29 ± 0.67  TH: -1.12 ± 0.85  FN: -1.57 ± 0.79  DR: -1.18 ± 1.14 | - | | | - | - | | | |  |
|  |  | 16 men |  | 15 men |  |  |  |  |  |  |  |  |  |  |  |  |  |  |  |  |  |
|  |  |  |  |  | Romosozumab | 1mg/kg sc every 2 weeks | **T-score**  **Baseline** | | | | | LS: -1.15 ± 0.76  TH: -0.90 ± 0.85  FN: -1.42 ± 0.89  DR: -0.23 ± 0.93 | - | | | - | - | | | |  |
|  |  |  |  |  |  | 3mg/kg sc every 4 weeks | **T-score**  **Baseline** | | | | | LS: -0.75 ± 1.16  TH: -0.55 ± 0.67  FN: -0.97 ± 0.63  DR: -0.08 ± 0.76 | - | | | - | - | | | |  |

BMD – Bone Mineral Density; BMC – Bone Mineral Content BA/TA – Bone Area per Total Area; BV/TV – Bone Volume Fraction; TM – Tibia Metaphysis; FN – Femoral Neck; Tb – Trabecular; Ct – Cortical; DF – Distal Femur; FD – Femoral Diaphysis; PTM – Proximal Tibia Metaphysis; NL – Normal-loaded; UL – Under-loaded; LV – 5^th^ Lumbar Vertebra; PT – Proximal Tibia; %Change – Percent change from Baseline; DXA – Dual energy X-ray Absorptiometry; pQCT – Peripheral Quantitative Computed Tomography; WB – Whole Body; LS – Lumbar Spine; UDR – Ultra-distal Radius; DRM – Distal Radius Metaphysis; DRD – Distal Radius Diaphysis; PTM – Proximal Tibial Metaphysis; CVB – 5^th^ Caudal Vertebral Body; LVB – 4^th^ Lumbar Vertebral Body; TH – Total Hip; DR – Third Distal Radius; mo – months.
