# Supplementary material for: Antisclerostin Effect on Osseointegration and Bone Remodeling
Source: J Clin Med. 2023 Feb 6;12(4):1294. doi: 10.3390/jcm12041294 (PMC9964545; doi:10.3390/jcm12041294)
Supplement: Supplementary file 1 [file jcm-12-01294-s001.zip › Suppl. Table 8.docx]

Table S8. Bone remodeling/formation parameters - Part II.

|  | Sample Size  (Initial) | | Sample Size  (Final) | | | Drug/Control | Dosage &  Administration Route | Bone Volume | | Bone Height | | | Bone Area | | |
| --- | --- | --- | --- | --- | --- | --- | --- | --- | --- | --- | --- | --- | --- | --- | --- |
| Liu *et al.*  (2018) [57] | 50 | 40 OVX | 50 | 40 OVX | | Scl-Ab VI | 18.2mg/kg sc twice week | - | | - | | | - | | |
|  |  |  |  |  |  | Scl-Ab VI + DAB | 18.1mg/kg sc + 18.1mg/kg sc twice week | - | | - | | | - | | |
|  |  |  |  |  |  | saline vehicle | - | - | | - | | | - | | |
|  |  | 10 Sham |  | 10 Sham | | saline vehicle | - | - | | - | | | - | | |
|  | 45 | | 45 | | | - | - | 9 w post extraction: decrease of 38% | | 9 w post extraction: fast vertical resorption | | |  | | |
|  |  |  |  |  |  | Scl-Ab VI | 25mg/kg sc twice week | **2 & 4 wks** | significant increase  further increases over time | **2 & 4 wks** | | significant increase, with further increases over time | - | | |
|  |  |  |  |  |  |  |  | **15 wks** | 42% higher alveolar bone ridge volume than control | **15 wks** | | recovery of ≈2/3 of total loss of bone height |  |  |  |
|  |  |  |  |  |  | Scl-Ab VI + DAB | 25mg/kg + 25mg/kg sc twice week | **2 & 4 wks** | significant increase  further increases over time | **2 & 4 wks** | | significant increase, with further increases over time | - | | |
|  |  |  |  |  |  |  |  | **15 wks** | 81% higher alveolar bone ridge volume than control | **9 wks** | | full recovery of bone height loss |  |  |  |
|  |  |  |  |  |  | saline vehicle | - | decrease over time | | **15 wks** | | resorption over time  total height loss = 0.41mm | - | | |
| Wu *et al.*  (2018) [60] | 40 OVX | |  | | | Scl-Ab | 25mg/kg sc twice week | - | | - | | | - | | |
|  |  |  |  |  |  | PTH 1-34 | 60𝜇g/kg sc thrice week | - | | - | | | - | | |
|  |  |  |  |  |  | Scl-Ab + PTH 1-34 | 25mg/kg sc twice week +  60𝜇g/kg sc thrice week | - | | - | | | - | | |
|  |  |  |  |  |  | vehicle | - | - | | - | | | - | | |
| Taut *et al.*  (2013) [65] | 69 | | 69 | | | EP: Scl-Ab III | 25 mg/kg sc twice week | - | | - | | | - | | |
|  |  |  |  |  |  |  | 15 𝜇L of 35.6mg/mL solution locally twice week | - | | - | | | - | | |
|  |  |  |  |  |  | EP: vehicle | - | - | | - | | | - | | |
|  |  |  |  |  |  | healthy: PBS | - | - | | - | | | - | | |
| Virk *et al.*  (2013) [58] | 72 | | 72 | | | Scl-Ab III | 25mg/kg sc twice week | 12 weeks: 29.7 ± 11.2 mm^3^  2 weeks: 22.7 ± 14.8 mm^3^  2-4 weeks: 25.7 ± 16.5 mm^3^ | | - | | | 0-12 weeks: 46.8 ± 16.2 mm^2^  0-2 weeks: 31.4 ± 20.1 mm^2^  2-4 weeks: 36 ± 17.4 mm^2^ | | |
|  |  |  |  |  |  | PBS | - | 18.3 ± 8.6 mm^3^ | | - | | | 30.3 ± 8.8 mm^2^ | | |
|  | 30 | | 30 | | | Scl-Ab III | 25mg/kg | 17.6 ± 7.4 mm^3^ | | - | | | 38.6 ± 23.8 mm^2^ | | |
|  |  |  |  |  |  | PBS | - | 8.5 ± 3.3 mm^3^ | | - | | | 13.1 ± 9.6 mm^2^ | | |
| McDonald *et al.* (2012) [33] | 132 | 66 Sham | 127 | | | Scl-Ab III | 25mg/kg sc twice week | - | | - | | | - | | |
|  |  |  |  |  |  | saline solution | - | - | | - | | | - | | |
|  |  | 66 OVX |  |  |  | Scl-Ab III | 25mg/kg sc twice week | - | | - | | | - | | |
|  |  |  |  |  |  | saline solution | - | - | | - | | | - | | |
| Ominsky *et al.*  (2011) [59] | 35 | | 32 | | | Scl-Ab III | 25mg/kg sc twice week | - | |  | | | - | | |
|  |  |  |  |  |  | vehicle | - | - | |  | | | - | | |
| Tian *et al*.  (2011) [34] | 67 | | 67 | | | Scl-Ab III | 5mg/kg sc twice week | - | | - | | | - | | |
|  |  |  |  |  |  |  | 25mg/kg sc twice week | - | | - | | | - | | |
|  |  |  |  |  |  | saline solution | - | - | | - | | | - | | |
| Li *et al.*  (2010) [38] | 28 | | 26 | | | Scl-Ab III | 25mg/kg sc twice week | - | | - | | | LV: 7.90 ± 0.30 mm^2^  FN: 4.73 ± 0.21 mm^2^ | | |
|  |  |  |  |  |  |  | 5mg/kg sc twice week | - | | - | | | LV: 6.84 ± 0.23 mm^2^  FN: 4.01 ± 0.18 mm^2^ | | |
|  |  |  |  |  |  | vehicle | - | - | | - | | | LV: 4.75 ± 0.20 mm^2^  FN: 3.69 ± 0.15 mm^2^ | | |
| Ominsky *et al.*  (2010) [64] | 12 | | 12 | | | Scl-Ab IV | 3mg/kg sc once month | - | | - | | | **pQCT** | | DRM: 2.2 ± 2.6 %  PTM: 5.7 ± 9.9 % |
|  |  |  |  |  |  |  | 10mg/kg sc once month | - | | - | | | **pQCT** | | DRM: -0.7 ± 2.1 %  PTM: 5.8 ± 2.6 % |
|  |  |  |  |  |  |  | 30mg/kg sc once month | - | | - | | | **pQCT** | | DRM: 4.7 ± 3.2 %  PTM: 7.0 ± 2.4 % |
|  |  |  |  |  |  | vehicle | - | **-** | | - | | | **pQCT** | | DRM: -1.7 ± 1.3 %  PTM: -3.6 ± 2.1 % |
| Tian *et al.*  (2010) [62] | 32 | | 32 | | | Scl-Ab III | 5mg/kg sc twice week | - | | - | | | - | | |
|  |  |  |  |  |  |  | 25mg/kg sc twice week | - | | - | | | - | | |
|  |  |  |  |  |  | saline solution | - | - | | - | | | - | | |
| Saag *et al.*  (2017) [67] | 4093 | | 3150 | | | Romosozumab → Alendronate | 210mg sc once month → 70mg po once week | - | | - | | | - | | |
|  |  |  |  |  |  | Alendronate → Alendronate | 70mg po once week → 70mg po once week | - | | - | | | - | | |
| McClung *et al.*  (2014) [41] | 419 | | 383 | | | Romosozumab | 140mg sc every 3 moths | - | | - | | | - | | |
|  |  |  |  |  |  |  | 210mg sc every 3 months | - | | - | | | - | | |
|  |  |  |  |  |  |  | 70mg sc once month | - | | - | | | - | | |
|  |  |  |  |  |  |  | 140mg sc once month | - | | - | | | - | | |
|  |  |  |  |  |  |  | 210mg sc once month | - | | - | | | - | | |
|  |  |  |  |  |  | Alendronate | 70 mg po once week | - | | - | | | - | | |
|  |  |  |  |  |  | Teriparatide | 20𝜇g sc once day | - | | - | | | - | | |
|  |  |  |  |  |  | placebo | - | - | | - | | | - | | |
| Padhi *et al.*  (2014) [43] | 48 | 32 women | 46 | | 31 women | Romosozumab | 1mg/kg sc every 2 weeks | - | | | - | | | - | |
|  |  |  |  |  |  |  | 2mg/kg sc every 4 weeks | - | | | - | | | - | |
|  |  |  |  |  |  |  | 2mg/kg sc every 2 weeks | - | | | - | | | - | |
|  |  |  |  |  |  |  | 3mg/kg sc every 4 weeks | - | | | - | | | - | |
|  |  |  |  |  |  | placebo | - | - | | | - | | | - | |
|  |  | 16 men |  |  | 15 men |  |  |  |  |  |  |  |  |  |  |
|  |  |  |  |  |  | Romosozumab | 1mg/kg sc every 2 weeks | - | | | - | | | - | |
|  |  |  |  |  |  |  | 3mg/kg sc every 4 weeks | - | | | - | | | - | |

Tb – Trabecular; LV – 5^th^ Lumbar Vertebra; DF – Distal Femur; FN – Femoral Neck; pQCT – Peripheral Quantitative Computed Tomography; DRM – Distal Radius Metaphysis; PTM – Proximal Tibial Metaphysis.
