# Supplementary material for: Antisclerostin Effect on Osseointegration and Bone Remodeling
Source: J Clin Med. 2023 Feb 6;12(4):1294. doi: 10.3390/jcm12041294 (PMC9964545; doi:10.3390/jcm12041294)
Supplement: Supplementary file 1 [file jcm-12-01294-s001.zip › Suppl. Table 9.docx]

Table S9. Bone remodeling/formation parameters - Part III.

|  | Sample Size  (Initial) | | | Sample Size  (Final) | | Drug/Control | Dosage &  Administration Route | Tb.Ar | Ct.Ar | | M.Ar | Tt.Ar | Ct.Ar/Tt.Ar |
| --- | --- | --- | --- | --- | --- | --- | --- | --- | --- | --- | --- | --- | --- |
| Liu *et al.*  (2018) [57] | 50 | 40 OVX | | 50 | 40 OVX | Scl-Ab VI | 18.2mg/kg sc twice week | - | - | | - | - | - |
|  |  |  |  |  |  | Scl-Ab VI + DAB | 18.1mg/kg sc + 18.1mg/kg sc twice week | - | - | | - | - | - |
|  |  |  |  |  |  | saline vehicle | - | - | - | | - | - | - |
|  |  | 10 Sham | |  | 10 Sham | saline vehicle | - | - | - | | - | - | - |
|  | 45 | | | 45 | | Scl-Ab VI | 25mg/kg sc twice week | - | - | | - | - | - |
|  |  |  |  |  |  | Scl-Ab VI + DAB | 25mg/kg sc + 25mg/kg sc twice week | - | - | | - | - | - |
|  |  |  |  |  |  | saline vehicle | - | - | - | | - | - | - |
| Wu *et al.*  (2018) [60] | 40 OVX | | | 40 OVX | | Scl-Ab | 25mg/kg sc twice week | - | - | | - | - | - |
|  |  |  |  |  |  | PTH 1-34 | 60𝜇g/kg sc thrice week | - | - | | - | - | - |
|  |  |  |  |  |  | Scl-Ab +  PTH 1-34 | 25mg/kg sc twice week + 60𝜇g/kg sc thrice week | - | - | | - | - | - |
|  |  |  |  |  |  | vehicle | - | - | - | | - | - | - |
| Taut *et al.*  (2013) [65] | 69 | | | 69 | | EP: Scl-Ab III | 25 mg/kg sc twice week | - | - | | - | - | - |
|  |  |  |  |  |  |  | 15 𝜇L of 35.6mg/mL solution locally twice week | - | - | | - | - | - |
|  |  |  |  |  |  | EP: vehicle | - | - | - | | - | - | - |
|  |  |  |  |  |  | healthy: PBS | - | - | - | | - | - | - |
| Virk *et al.*  (2013) [58] | 72 | | | 72 | | Scl-Ab III | 25mg/kg sc twice week | - | - | | - | - | - |
|  |  |  |  |  |  | PBS | - | - | - | | - | - | - |
|  | 30 | | | 30 | | Scl-Ab III | 25mg/kg | - | - | | - | - | - |
|  |  |  |  |  |  | PBS | - | - | - | | - | - | - |
| McDonald *et al.* (2012) [33] | 132 | | 66 Sham | 127 | | Scl-Ab III | 25mg/kg sc twice week | - | - | | - | - | - |
|  |  |  |  |  |  | saline solution | - | - | - | | - | - | - |
|  |  |  | 66 OVX |  |  | Scl-Ab III | 25mg/kg sc twice week | - | - | | - | - | - |
|  |  |  |  |  |  | saline solution | - | - | - | | - | - | - |
| Ominsky *et al.*  (2011) [59] | 35 | | | 32 | | Scl-Ab III | 25mg/kg sc twice week | - | - | | - | - | - |
|  |  |  |  |  |  | vehicle | - | - | - | | - | - | - |
| Tian *et al*.  (2011) [34] | 67 | | | 67 | | Scl-Ab III | 5mg/kg sc. twice week | - | - | | - | - | - |
|  |  |  |  |  |  |  | 25mg/kg sc. twice week | - | - | | - | - | - |
|  |  |  |  |  |  | saline solution | - | - | - | | - | - | - |
| Li *et al.*  (2010) [38] | 28 | | | 26 | | Scl-Ab III | 25mg/kg sc. twice week | LV: 3.15 ± 0.20 mm^2^ | **HMM** | TS 6.80 ± 0.24 mm^2^ | TS: 0.92 ± 0.04 mm^2^ | TS: 7.72 ± 0.26 mm^2^ | TS: 83.2 ± 1.1 % |
|  |  |  |  |  |  |  |  |  | **𝜇CT** | LV: 4.75 ± 0.12 mm^2^  FD: 11.67 ± 0.32 mm^2^ |  |  |  |
|  |  |  |  |  |  |  | 5mg/kg sc. twice week | LV: 2.55 ± 0.14 mm^2^ | **HMM** | TS 5.79 ± 0.48 mm^2^ | TS: 0.97 ± 0.09 mm^2^ | TS: 6.76 ± 0.51 mm^2^ | TS: 88.0 ± 0.5 % |
|  |  |  |  |  |  |  |  |  | **𝜇CT** | LV: 4.29 ± 0.12 mm^2^  FD: 11.62 ± 0.43 mm^2^ |  |  |  |
|  |  |  |  |  |  | vehicle | - | LV: 1.67 ± 0.11 mm^2^ | **HMM** | TS 6.17 ± 0.13 mm^2^ | TS: 1.25 ± 0.09 mm^2^ | TS: 7.43 ± 0.15 mm^2^ | TS: 85.3 ± 1.3 % |
|  |  |  |  |  |  |  |  |  | **𝜇CT** | LV: 3.08 ± 0.11 mm^2^  FD: 9.67 ± 0.32 mm^2^ |  |  |  |
| Ominsky *et al.*  (2010) [64] | 12 | | | 12 | | Scl-Ab IV | 3mg/kg sc. once month | - | **pQCT** | DRD: 7.2 ± 8.5 %  PTD: 11.5 ± 13.5% | - | - | - |
|  |  |  |  |  |  |  | 10mg/kg sc. Once month | - | **pQCT** | DRD: 5.0 ± 3.3 %  PTD: 12.1 ± 3.0 % | - | - | - |
|  |  |  |  |  |  |  | 30mg/kg sc. once month | - | **pQCT** | DRD: 10.0 ± 4.2 %  PTD: 12.6 ± 3.7 % | - | - | - |
|  |  |  |  |  |  | vehicle | - | **-** | **pQCT** | DRD: 2.8 ± 1.2 %  PTD: 1.2 ± 3.2 % | - | - | - |
| Tian *et al.*  (2010) [62] | 32 | | | 32 | | Scl-Ab III | 5mg/kg sc. twice week | - | - | | - | - | - |
|  |  |  |  |  |  |  | 25mg/kg sc. twice week | - | - | | - | - | - |
|  |  |  |  |  |  | saline solution | - | - | - | | - | - | - |
| Saag *et al.*  (2017) [67] | 4093 | | | 3150 | | Romosozumab → Alendronate | 210mg sc. once month → 70mg po. once week | - | - | | - | - | - |
|  |  |  |  |  |  | Alendronate → Alendronate | 70mg po. once week → 70mg po. once week | - | - | | - | - | - |
| McClung *et al.*  (2014) [41] | 419 | | | 383 | | Romosozumab | 140mg sc. every 3 months | - | - | | - | - | - |
|  |  |  |  |  |  |  | 210mg sc. every 3 months | - | - | | - | - | - |
|  |  |  |  |  |  |  | 70mg sc. once month | - | - | | - | - | - |
|  |  |  |  |  |  |  | 140mg sc. once month | - | - | | - | - | - |
|  |  |  |  |  |  |  | 210mg sc. once month | - | - | | - | - | - |
|  |  |  |  |  |  | Alendronate | 70 mg po. once week | - | - | | - | - | - |
|  |  |  |  |  |  | Teriparatide | 20𝜇g sc. once day | - | - | | - | - | - |
|  |  |  |  |  |  | placebo | - | - | - | | - | - | - |
| Padhi *et al.*  (2014) [43] | 48 | 32 women | | 46 | 31 women | romosozumab | 1mg/kg sc. every 2 weeks | - | - | | - | - | - |
|  |  |  |  |  |  |  | 2mg/kg sc. every 4 weeks | - | - | | - | - | - |
|  |  |  |  |  |  |  | 2mg/kg sc. every 2 weeks | - | - | | - | - | - |
|  |  |  |  |  |  |  | 3mg/kg sc. every 4 weeks | - | - | | - | - | - |
|  |  |  |  |  |  | placebo | - | - | - | | - | - | - |
|  |  | 16 men | |  | 15 men |  |  |  |  |  |  |  |  |
|  |  |  |  |  |  | romosozumab | 1mg/kg sc. every 2 weeks | - | - | | - | - | - |
|  |  |  |  |  |  |  | 3mg/kg sc. every 4 weeks | - | - | | - | - | - |
|  |  |  | |  |  |  |  |  |  | |  |  |  |

Tb.Ar Trabecular Area; Ct.Ar – Cortical Area; M.Ar – Medullary Area; Tt.Ar - Total cross-sectional Area/Subperiosteal Area; Ct.Ar/Tt.Ar – Cortical Area per Total Cross-sectional Area; LV – 5^th^ Lumbar Vertebra; HMM – Histomorphometry; 𝜇CT – Micro computed tomography; TS – Tibial Shaft; FD – Femoral Diaphysis; pQCT – Peripheral Quantitative Computed Tomography; pQCT – Peripheral Quantitative Computed Tomography; DRD – Distal Radius Diaphysis; PTD – Proximal Tibial Diaphysis.
